# Synthesis and Cytotoxic Activity of Combretastatin A-4 and 2,3-Diphenyl-2*H*-indazole Hybrids

**DOI:** 10.3390/ph14080815

**Published:** 2021-08-19

**Authors:** Jaime Pérez-Villanueva, Félix Matadamas-Martínez, Lilián Yépez-Mulia, Vadim Pérez-Koldenkova, Martha Leyte-Lugo, Karen Rodríguez-Villar, Francisco Cortés-Benítez, Ana Perla Macías-Jiménez, Ignacio González-Sánchez, Ariana Romero-Velásquez, Juan Francisco Palacios-Espinosa, Olivia Soria-Arteche

**Affiliations:** 1 Departamento de Sistemas Biológicos, División de Ciencias Biológicas y de la Salud, Universidad Autónoma Metropolitana-Xochimilco (UAM-X), Ciudad de México 04960, Mexico; felixmatadamas@yahoo.com.mx (F.M.-M.); jcortesb@correo.xoc.uam.mx (F.C.-B.); anmacp61@gmail.com (A.P.M.-J.); jpalacios@correo.xoc.uam.mx (J.F.P.-E.); soriao@correo.xoc.uam.mx (O.S.-A.); 2 Maestría y Doctorado en Ciencias Farmacéuticas, División de Ciencias Biológicas y de la Salud, Universidad Autónoma Metropolitana-Xochimilco (UAM-X), Ciudad de México 04960, Mexico; 3 Unidad de Investigación Médica en Enfermedades Infecciosas y Parasitarias, UMAE Hospital de Pediatría, Centro Médico Siglo XXI, Instituto Mexicano del Seguro Social, Ciudad de México 06720, Mexico; 4 Laboratorio Nacional de Microscopía Avanzada, Centro Médico Nacional Siglo XXI, Instituto Mexicano del Seguro Social, Ciudad de México 06720, Mexico; vadim.perez@imss.gob.mx; 5 Catedrático CONACYT Comisionado a Departamento de Sistemas Biológicos, División de Ciencias Biológicas y de la Salud, Universidad Autónoma Metropolitana-Xochimilco (UAM-X), Ciudad de México 04960, Mexico; mleyte@correo.xoc.uam.mx (M.L.-L.); ignacio.gonzalez.s@gmail.com (I.G.-S.); 6 Doctorado en Ciencias Biológicas y de la Salud, Universidad Autónoma Metropolitana (UAM), Ciudad de México 04960, Mexico; qkarenrodv@hotmail.com; 7 Maestría en Ciencias Biológicas, Universidad Nacional Autónoma de México (UNAM), Ciudad de México 04510, Mexico; ariromerov@gmail.com

**Keywords:** cancer, combretastatin A-4, cytotoxic activity, hybrid compounds, indazole

## Abstract

Cancer is the second leading cause of death, after cardiovascular diseases. Different strategies have been developed to treat cancer; however, chemotherapy with cytotoxic agents is still the most widely used treatment approach. Nevertheless, drug resistance to available chemotherapeutic agents is still a serious problem, and the development of new active compounds remains a constant need. Taking advantage of the molecular hybridization approach, in the present work we designed, synthesized, and tested the cytotoxic activity of two hybrid compounds and seven derivatives based on the structure of combretastatin A-4 and 2,3-diphenyl-2*H*-indazole. Practical modifications of reported synthetic protocols for 2-pheny-2*H*-indazole and 2,3-dipheny-2*H*-indazole derivatives under microwave irradiation were implemented. The cytotoxicity assays showed that our designed hybrid compounds possess strong activity, especially compound **5**, which resulted even better than the reference drug cisplatin against HeLa and SK-LU-1 cells (IC_50_ of 0.16 and 6.63 µM, respectively), and it had similar potency to the reference drug imatinib against K562 cells. Additionally, in silico and in vitro studies strongly suggest tubulin as the molecular target for hybrid compound **5**.

## 1. Introduction

Cancer is “a group of diseases characterized by uncontrollable growth and spread of abnormal cells, which can then invade adjoining parts of the body and spread to other organs” [1,2]. Globally, after cardiovascular diseases, cancer is the second leading cause of death [2]. Different strategies have been developed to treat cancer; however, chemotherapy with cytotoxic agents is still the most widely used treatment approach. Nevertheless, drug resistance to available chemotherapeutic agents is still a serious problem, and the development of new active compounds remains a constant need [3].

**Figure 1:**
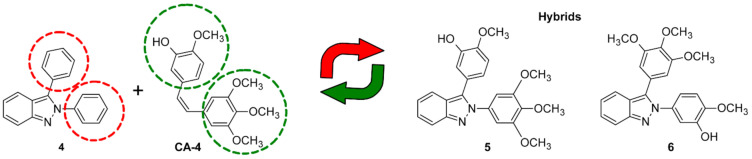
Molecular design of hybrid compounds.

The indazole nucleus is considered an important heterocyclic moiety in medicinal chemistry, since several derivatives harboring this scaffold have shown anticancer, hypoglycemic, antiprotozoal, anti-inflammatory, antimicrobial, anti-HIV, and antihypertensive activities [4,5,6,7]. Furthermore, some drugs containing the indazole nucleus have been approved as anticancer agents (e.g., pazopanib, axitinib, and niraparib) [6]. Previous studies focused on the search of antiprotozoal compounds showed that 2,3-diphenyl-2*H*-indazole (**4**) exhibits cytotoxic activity against HeLa cells (IC_50_ = 125 µM) [8]. Although the cytotoxic activity of this compound is weak, it reveals itself as a new scaffold of interest to develop new cytotoxic agents with the appropriate substituents. Hence, taking advantage of the molecular hybridization approach, in the present work we designed, synthesized, and tested the cytotoxic activity of two hybrid compounds (**5** and **6**) inspired on 2,3-diphenyl-2*H*-indazole and the natural product Combretastatin A-4 (**CA-4**, Figure 1). **CA-4**, isolated from the bushwillow tree *Combretum caffrum*, is a well-known cytotoxic stilbenoid that acts as a tubulin polymerization inhibitor by binding to the colchicine site [9]. The hybridization of both molecules, 2,3-diphenyl-2*H*-indazole and **CA-4**, was pursued to increase the cytotoxic effect by combination of two biologically active structures [8,9,10]. Additionally, the stabilization of the cis-diphenyl pattern, that can isomerize into the trans isomer in **CA-4** (less active), could be achieved by replacing the ethylene bridge in **CA-4** by the indazole moiety [9,10]. To learn more about the structural requirements of hybrid compounds to exhibit cytotoxic activity, structurally simplified derivatives (**1**–**3** and **7**–**9**) and the unsubstituted reference compound **4** are also synthesized and their cytotoxic activity tested. In addition, the effect of the best cytotoxic compound is characterized and its effect on tubulin is studied by in silico and in vitro assays. 

## 2. Results and Discussion

### 2.1. Chemistry

**Scheme 1:**
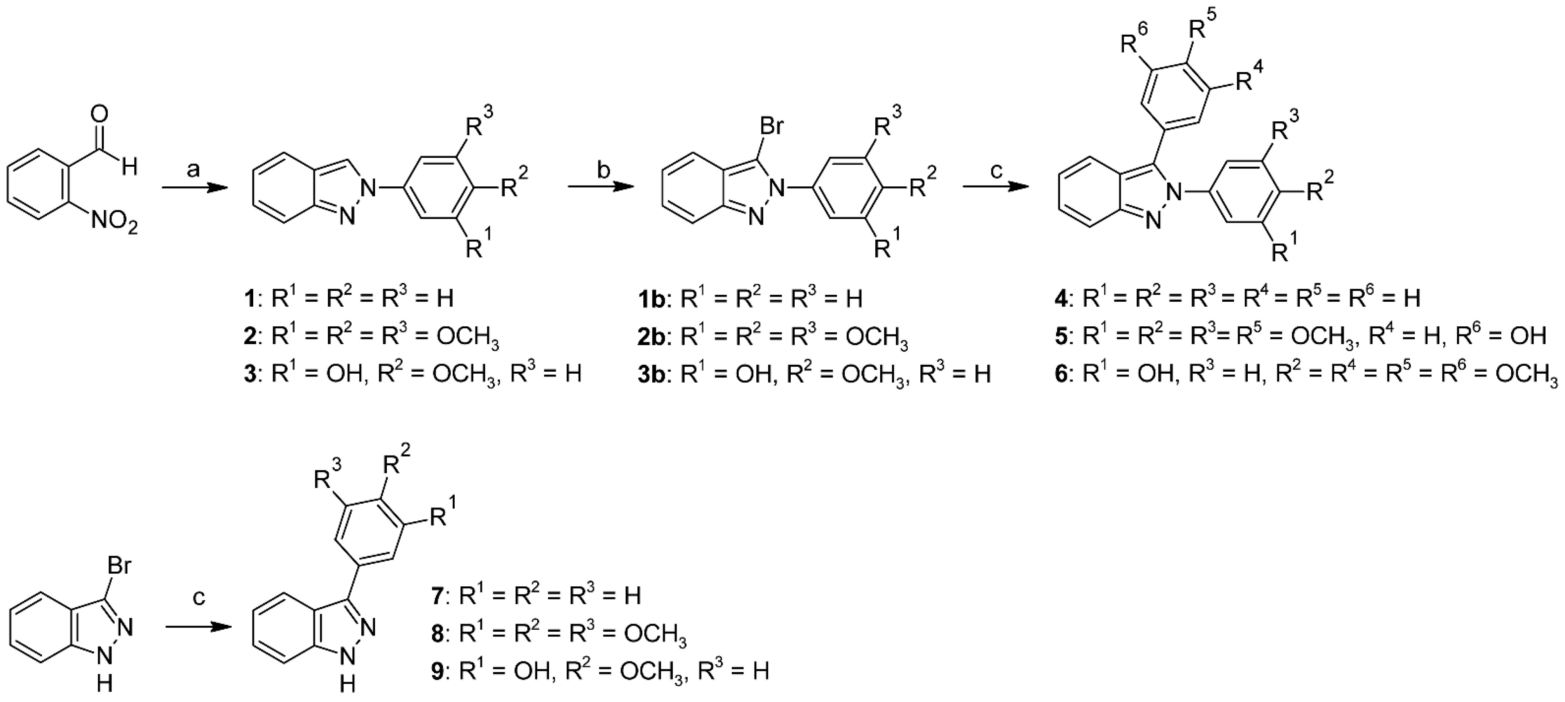
Synthesis of indazole derivatives **1**–**9**. Reagents and conditions: (a) 1. Aniline or substituted aniline, EtOH; 2. P(OEt)_3_, 150 °C; (b) Br_2_, AcOH; (c) appropriate phenylboronic acid or phenylboronic acid pinacol ester, 1-propanol, Pd(OAc)_2_, (C_6_H_5_)_3_P, Na_2_CO_3_, H_2_O.

The hybrid compounds and their derivatives (**1**–**9**) were synthesized as illustrated in Scheme 1. Compounds **1**–**3** were obtained by a slight modification of Cadogan’s method, where the commercially available 2-nitrobenzaldehyde was heated with aniline or the appropriate substituted aniline under microwave irradiation to afford the corresponding Schiff base, which was then treated with triethyl phosphite to give the 2-phenyl-2*H*-indazole derivatives **1**–**3** [11]. Compounds **1**–**3** were selectively brominated at the three-position employing a previously described method using bromine in acetic acid [12]. The 2,3-diphenyl-2*H*-indazole (**4**) and derivatives (**5** and **6**) were synthesized by a palladium-catalyzed coupling reaction, employing a slight modification under microwave irradiation of the previously reported conventional method under reflux [13]. It is worth mentioning that the microwave irradiation method gave the best yields and improved the reaction times for compounds **4**–**6** as compared to the same examples employing the original report (see Table S1 in the supporting information). Finally, employing the commercially available 3-bromo-1*H*-indazole as starting material, 3-phenyl-1*H*-indazole derivatives **7**–**9** were synthesized using this same microwave-assisted method. Compounds **5** and **9** were obtained by using the commercially available 3-acetoxy-4-methoxyphenylboronic acid pinacol ester; nonetheless, the 3-acetoxy group was hydrolyzed in situ under the basic reaction conditions to directly obtain the desired compounds (**5** and **9**). All synthesized compounds were characterized by ^1^H NMR and ^13^C NMR spectroscopy. The data on previously reported structures were consistent with literature reports (**1**, **2**, **4** and **7**). It is important to note that hybrids **5** and **6**, and derivatives **3**, **8**, and **9**, resulted in new structures, which were also characterized by mass spectrometry; particularly, the most active compound **5** was also characterized by high resolution mass spectrometry. The nuclear magnetic resonance and mass spectra of these compounds can be found in the Supporting Information (Figures S1–S29).

### 2.2. Cytotoxicity Assays

**CA-4** and several **CA-4** derivatives have been shown to have cytotoxic potency and anti-proliferative activity in a variety of human cancer cells, such as non-small cell lung cancer [14], ovarian cancer [15], and human leukemia cells [16,17], among others [18]. Therefore, in this study, three cancer cell lines—HeLa (human cervix), SK-LU-1 (human non-small cell lung), and K562 (human chronic myelogenous leukemia)—were selected. It is worth mentioning that the cell lines selected are representative of some high incidence cancer types [2]. The antiproliferative activity of all compounds (**1**–**9**) was initially tested at 50 micromolar after 48 h of exposition using the MTT assay on HeLa cells. Briefly, hybrids **5** and **6** possessed the best cytotoxic effect at 50 µM (Table S2). This result suggests that the entire 2,3-diphenyl-2*H*-indazole scaffold is important for the cytotoxic effect. The simplified structures based on 2-phenyl-2*H*-indazole (**1**–**3**) and 3-phenyl-1*H*-indazole derivatives (**7**–**9**) at the same concentration resulted in higher viability values. Thus, we decided to determine IC_50_ values for compounds **5** and **6** on HeLa, SK-LU-1, and K562 cells. Compound **4** was included as unsubstituted reference, whereas cisplatin and imatinib were included as reference drugs, Table 1. The results of IC_50_ showed the major effect for substituted hybrids (**5** and **6**) on HeLa, SK-LU-1, and K562 cells as compared with the unsubstituted compound **4**. Compound **5** displayed higher cytotoxic effect than **6** against the three cell lines: particularly, with an IC_50_ value of 0.16 µM against HeLa cells, making compound **5** 241-fold more active than **6**. Compound **5** was 116 times more active against HeLa cells and slightly more active against SK-LU-1 cells (two-fold) than the reference drug cisplatin, which is employed for cervical and lung cancer therapy [19]. Additionally, compound **5** showed against K562 cells, similar in vitro activity to imatinib, a drug used for the therapy of chronic myelogenous leukemia [20]. Cytotoxicity values for **CA-4** has been extensively reported on HeLa cells after 48 h by MTT assay. In ChEMBL database [21], 18 reference values were found with IC_50_ ranging from 0.003 to 14.830 µM, with a median value of 0.011 µM, which indicates that **CA-4** is still 15-fold more cytotoxic than compound **5** (0.16 µM). Whereas no reports were found for **CA-4** on SK-LU-1 cells, two reference values were found on K562 cells under the same assay conditions (0.0048 and 0.046 µM) [21]. Therefore, the cytotoxic activity for compound **5** is still lower as compared with previous **CA-4** reports, but it showed higher activity than cisplatin and imatinib, the reference drugs included in this study (Table 1). On the other hand, some studies suggest that antioxidants may lead to interferences with the MTT assay [22,23]; hence, the antioxidant activity of compounds **4**–**6**, was studied employing the DPPH and ABTS models. Results can be found at the supporting information (Table S3). Compounds were inactive on DPPH test, whereas very poor effect was found on ABTS test for compounds **5** and **6**, with IC_50_ values of 216 and 262 µM, respectively. Therefore, the intrinsic reductive potential of the compounds tested is not involved in the determination of IC_50_ values on MTT assays. In addition, the purity of the compounds **4**, **5**, and **6** was determined by quantitative NMR to ensure that the observed IC_50_ values were caused by the pure compounds (purity > 95%, Table S4). 

Bright-field microscopy reveals the presence of spherical cells on HeLa cultures treated with hybrid compounds **5** and **6** after 48 h (Figure S30 in supporting information) as compared with the typically observed epithelial polygonal morphology. This result and the reported effect of **CA-4** on microtubule assemblage, allowed us to consider microtubules as a possible target of the synthesized hybrids. Hence, in silico and in vitro studies were also performed.

### 2.3. Molecular Docking

**Figure 2:**
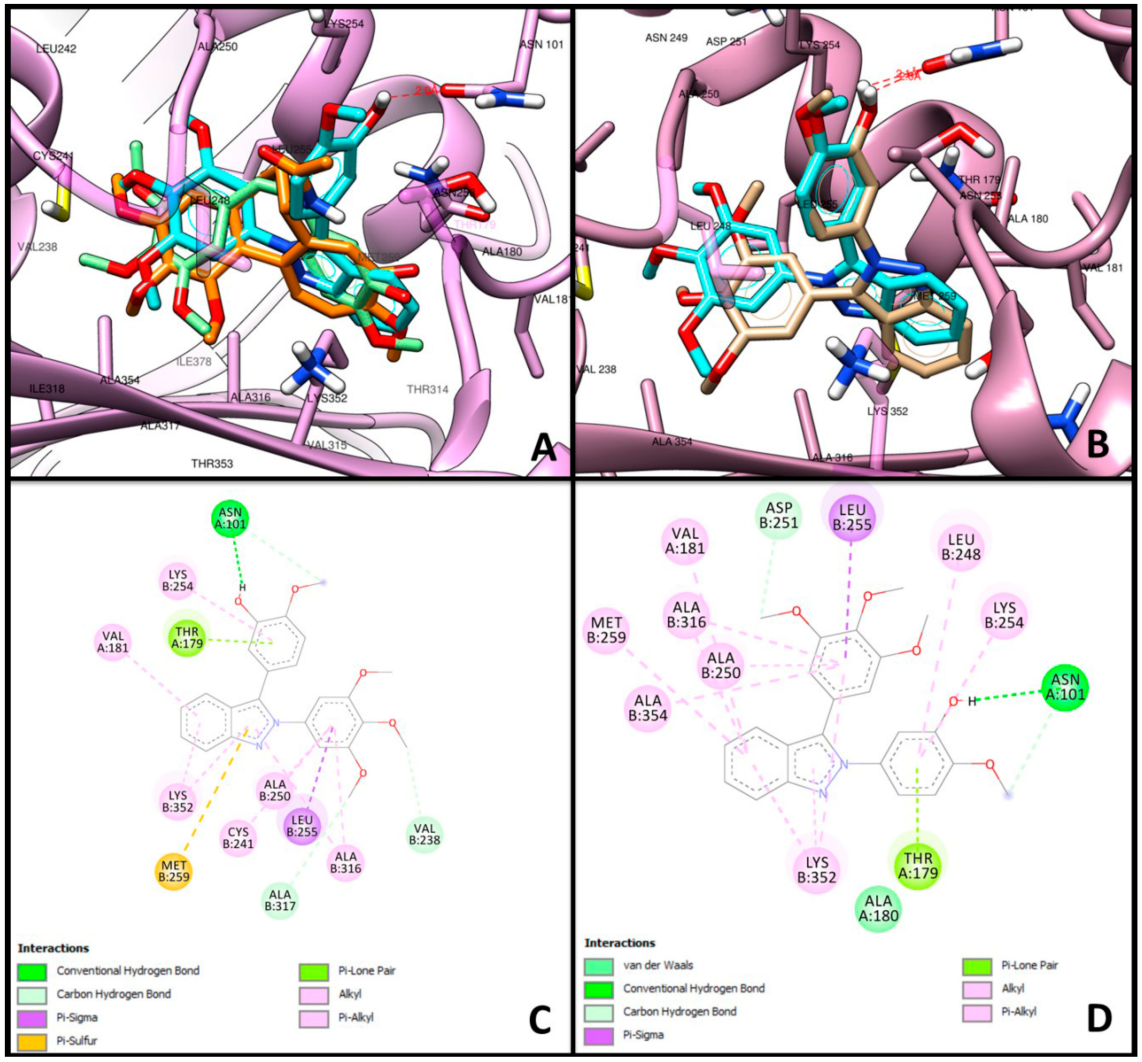
Molecular coupling of compounds **5** and **6** at the colchicine site on beta-tubulin. (**A**) Binding mode of colchicine (orange), **CA-4** (green) and compound **5** (cyan) docked into the colchicine binding site cavity (lilac) (PDB: 5LYJ). (**B**) Binding mode of compound **5** (cyan) and **6** (khaki) docked into the colchicine binding site cavity. 2D diagram of docked compounds **5** (**C**) and **6** (**D**) into the colchicine binding site cavity showing their interaction with the binding site residues.

To investigate the potential binding mode of hybrids **5** and **6** and their derivatives (**1**–**4** and **7**–**9**) to the colchicine binding site of beta-tubulin, we performed docking simulations using AutoDock 4.2 and the X-Ray structure of Tubulin-cis-**CA-4** Complex (PDB: 5LYJ) [24,25]. For comparison, **CA-4** and colchicine were also docked. The comparison of co-crystalized and docked **CA-4** structures superimposes with Root Mean Square Deviation (RMSD) of 0.835 Å, validating our method. As expected, **CA-4** and colchicine fit at the binding site with ΔG values of −7.45 and −8.24 kcal/mol, respectively. Interestingly, we found an improved docking score for indazole inhibitors **4** (−8.92 kcal/mol), **5** (−9.90 kcal/mol), and **6** (−9.60 kcal/mol) suggesting their higher affinity. It is worth to emphasize the score found for the 2-phenyl-2*H*-indazole derivatives **1**–**3** (−7.06, −6.73, and −6.49 kcal/mol respectively) and the 3-phenyl-1*H*-indazole derivatives **7**–**9** (−5.81, −6.79, and −6.50 kcal/mol respectively) suggesting a lower affinity. Moreover, the 3,4,5-trimethoxyphenyl at N2 or C3 for **5** and **6**, respectively, is oriented in same way as found in colchicine and **CA-4** (Figure 2). Furthermore, this moiety produces hydrophobic interactions with Ala250, Ala316 and π-σ interactions with Leu255. Unlike **CA-4**, the 3-hydroxy-4-methoxy substituted ring at C3 or N2 for **5** and **6**, respectively, is positioned to form an H-bond with the side chain of Asn101 (distance = 2.0 Å and 2.1 Å for compounds **5** and **6**, respectively), hydrophobic interactions with Lys254 as well as π-interactions with Thr179. Conversely, the indazole core of both analogs is oriented into a cavity and is further stabilized by hydrophobic interactions with residues Val181, Ala316, and Lys352. Surprisingly, compound **5** produced an extra π-sulfur contact with Met259. Thus, this compound fits better into the colchicine binding site of tubulin, providing slightly better binding energy than obtained for compound **6**.

### 2.4. In Vitro Tubulin Polymerization Assays

**Figure 3:**
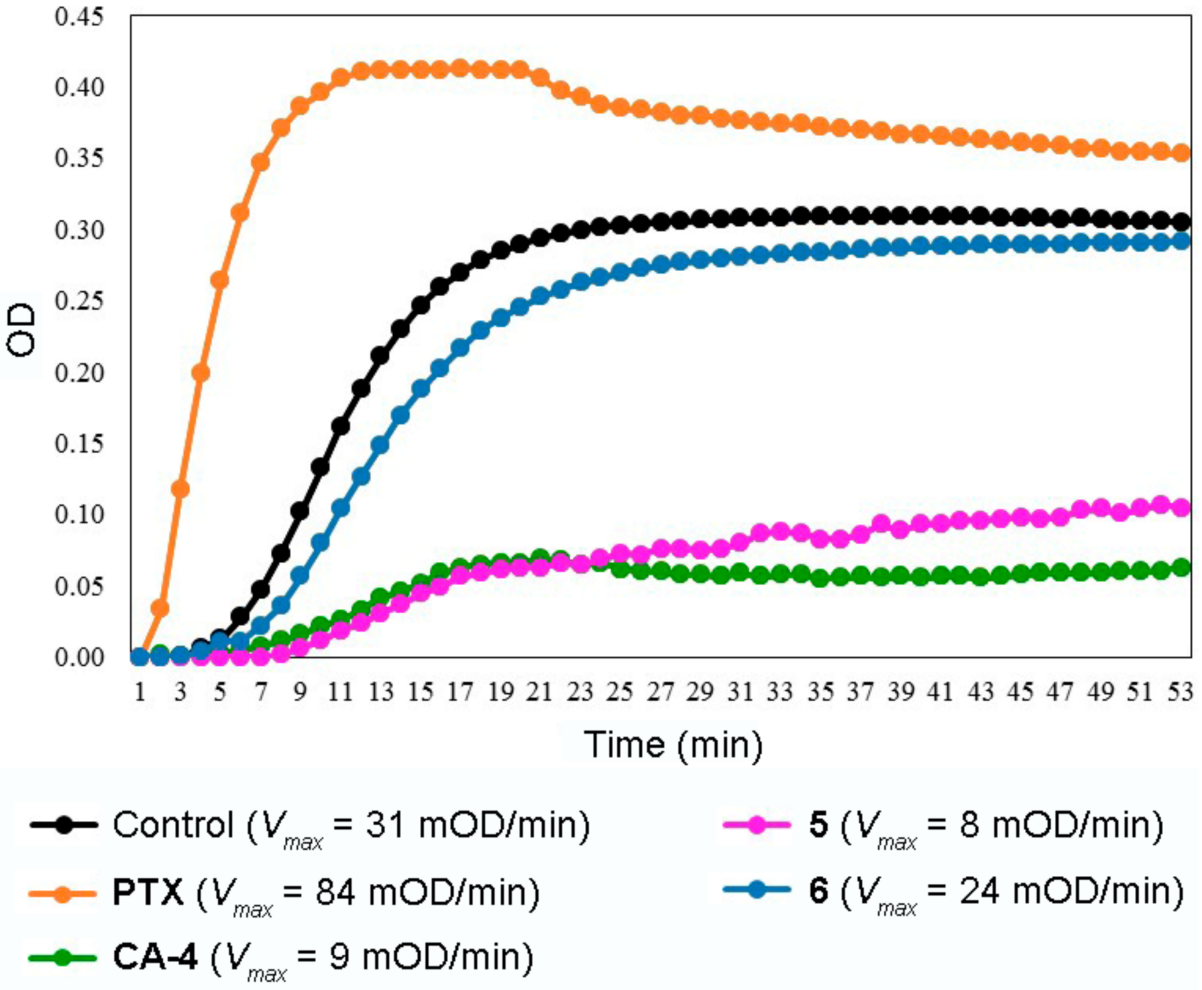
Effect of hybrid compounds **5** and **6** on tubulin polymerization. Polymerization reactions were carried out by incubation at 37 °C and followed by absorbance readings at 340 nm for 53 min at 37 °C. **CA-4** and **PTX** were used as positive controls. All compounds were used at a final concentration of 10 µM.

To learn more about the effect of compounds **5** and **6** on tubulin polymerization, we conducted a polymerization assay [26,27,28]. Figure 3 shows the drug-dependent effect on tubulin polymerization, in presence of paclitaxel (**PTX**), **CA-4** and hybrid compounds **5** and **6** or vehicle. The polymerization *V_max_* value was enhanced 2.7 times in the presence of the stabilizing agent **PTX**, with respect to vehicle (84 vs. 31 mOD/min). On the other hand, the microtubule destabilizing drug, **CA-4**, reduced 3.4 times the *V_max_* value as compared to vehicle (9 vs. 31 mOD/min). Compounds **5** and **6** decreased the polymerization *V_max_*, 3.8 and 1.3 times, respectively, in comparison to vehicle (8 and 24 vs. 31 mOD/min). These results suggest that hybrid compound **5** is a potential inhibitor of tubulin polymerization that displays an effect similar to that of **CA-4** at 10 µM.

### 2.5. Indirect Immunofluorescence (IFI)

**Figure 4:**
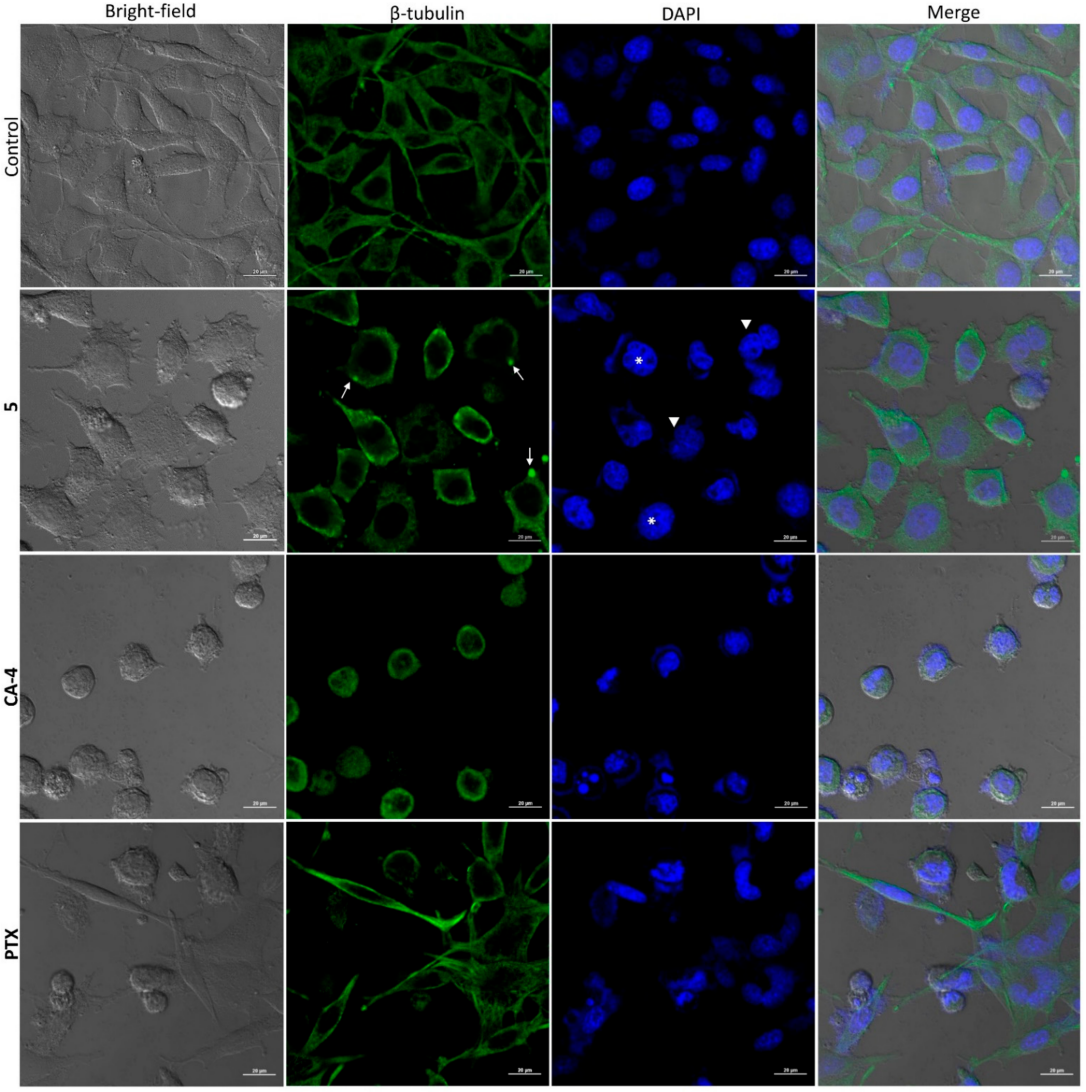
Effect of compound **5**, **PTX**, and **CA-4** on HeLa cells tubulin network analyzed by indirect immunofluorescence. Cells were treated with DMSO at 0.01% (Control), compound **5** (0.16 μM), **CA-4** (1.49 μM) and **PTX** (0.08 μM) for 24 h. Cell samples were incubated with anti-beta-tubulin antibody at 1:1000 dilution and anti-mouse IgG-FITC diluted 1:1000 as secondary antibody. The nuclear DNA was stained with 4,6-diamidino-2-phenylindole (DAPI). Beta-tubulin is stained in green, DNA in blue. First column: Bright-field images, second column: Beta-tubulin array, stained with FITC, third column: DAPI stain, fourth column: Merge. Samples were visualized by confocal microscopy. Arrow: Tubulin aggregates; Arrowhead: Multinucleation; Asterisk: Macronuclei. Scale bar: 20 μm.

The effect of compounds **5**, **PTX** and **CA-4** on HeLa cells tubulin network was examined by IFI (Figure 4). The well-organized tubulin network can be observed on untreated HeLa cells. In the case of **PTX**, a microtubule stabilizing agent, it leaded to an asymmetrical cell shape, and collapse of the tubulin network with the appearance of microtubule arrays and tubulin bundles. Compound **5** induced a loss of organized microtubule structure; tubulin staining was diffuse and disorganized with aggregated staining in parts. In addition, morphological changes such as round-shaped cells, formation of protuberances in the cytoplasm, nucleus size increase, and multinucleation were observed, all of them indicative features of mitotic catastrophe [29]. Meanwhile, treatment with **CA-4**, a destabilizing agent, resulted in small round-shaped cells with a disrupted microtubule cytoskeleton; multiple punctuate signals of beta tubulin were visible in the cytoplasm while nuclear damage characterized by multilobular deformed nuclei and micronuclei was evident.

**Figure 5:**
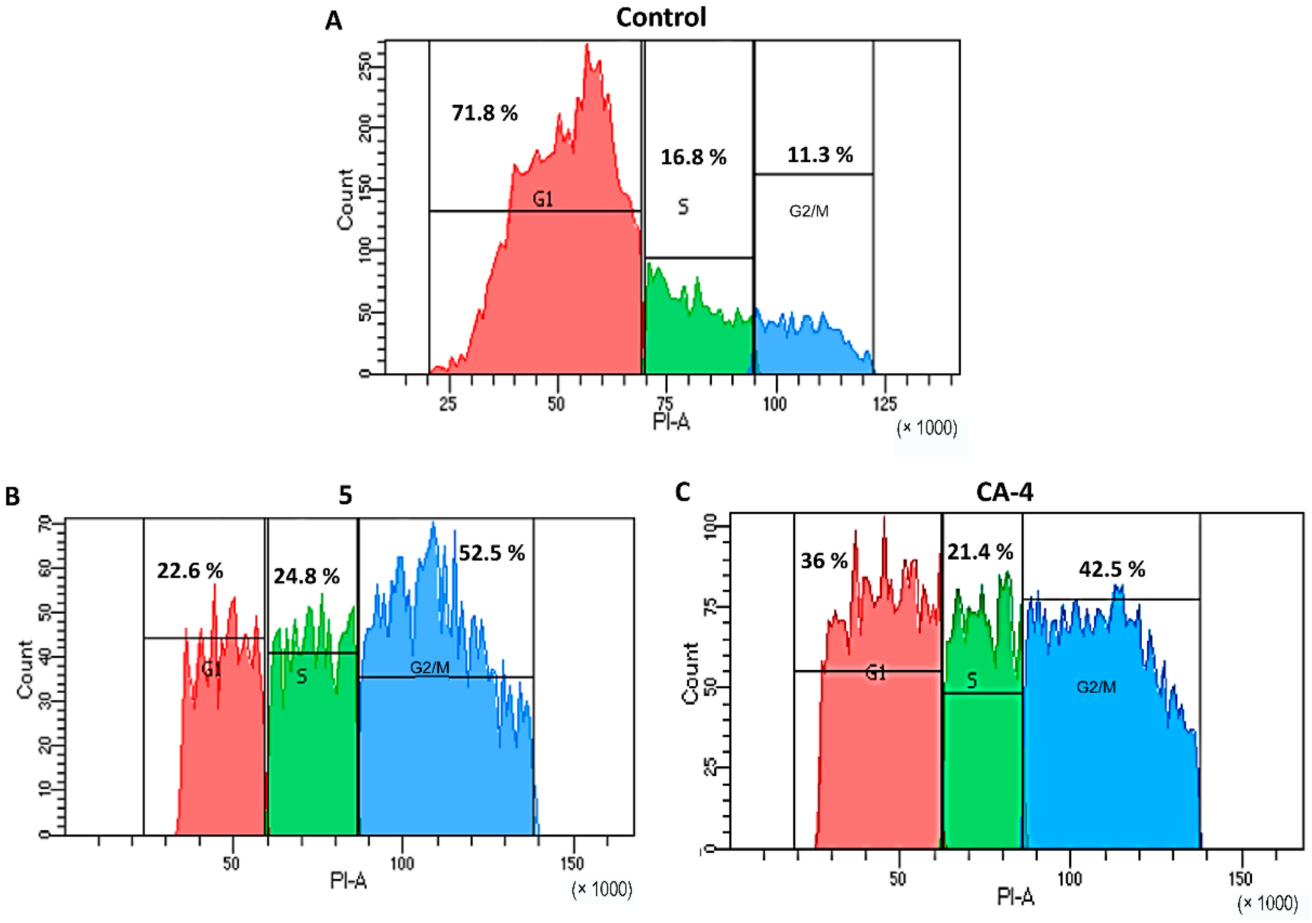
Analysis of cell cycle progression of HeLa cells by flow cytometry: (**A**) Control cells, (**B**) treated with compound **5**, (**C**) treated with **CA-4**. HeLa cells were treated with compound **5** (0.16 μM) and **CA-4** (1.49 μM) for 48 h.

It is known that the interference of microtubule dynamics by **CA-4** and some derivatives consequently disrupts mitotic progression and induce cytostasis and ultimately leads to apoptosis and cell death [30,31,32]. Therefore, the effect of compound **5** on the cell cycle of HeLa cells was analyzed by flow cytometry. Data showed that, in fact, HeLa cells were arrested in G_2_/M phase by compound **5** (52.5%), sharing this feature with **CA-4** (42.5%) (Figure 5). In addition, it was also shown that compound **5** triggers the apoptosis of HeLa cells (Figure S31 in supplementary information). Our data agrees with the mechanism of action of different microtubule depolymerizing agents such as nocodazole [33], colchicine [34], and **CA-4** and its derivatives [31,35] that induce the program cell death.

### 2.6. Effect of Compound ***5***, ***PTX***, and ***CA-4*** on Soluble and Polymerized Tubulin

**Figure 6:**
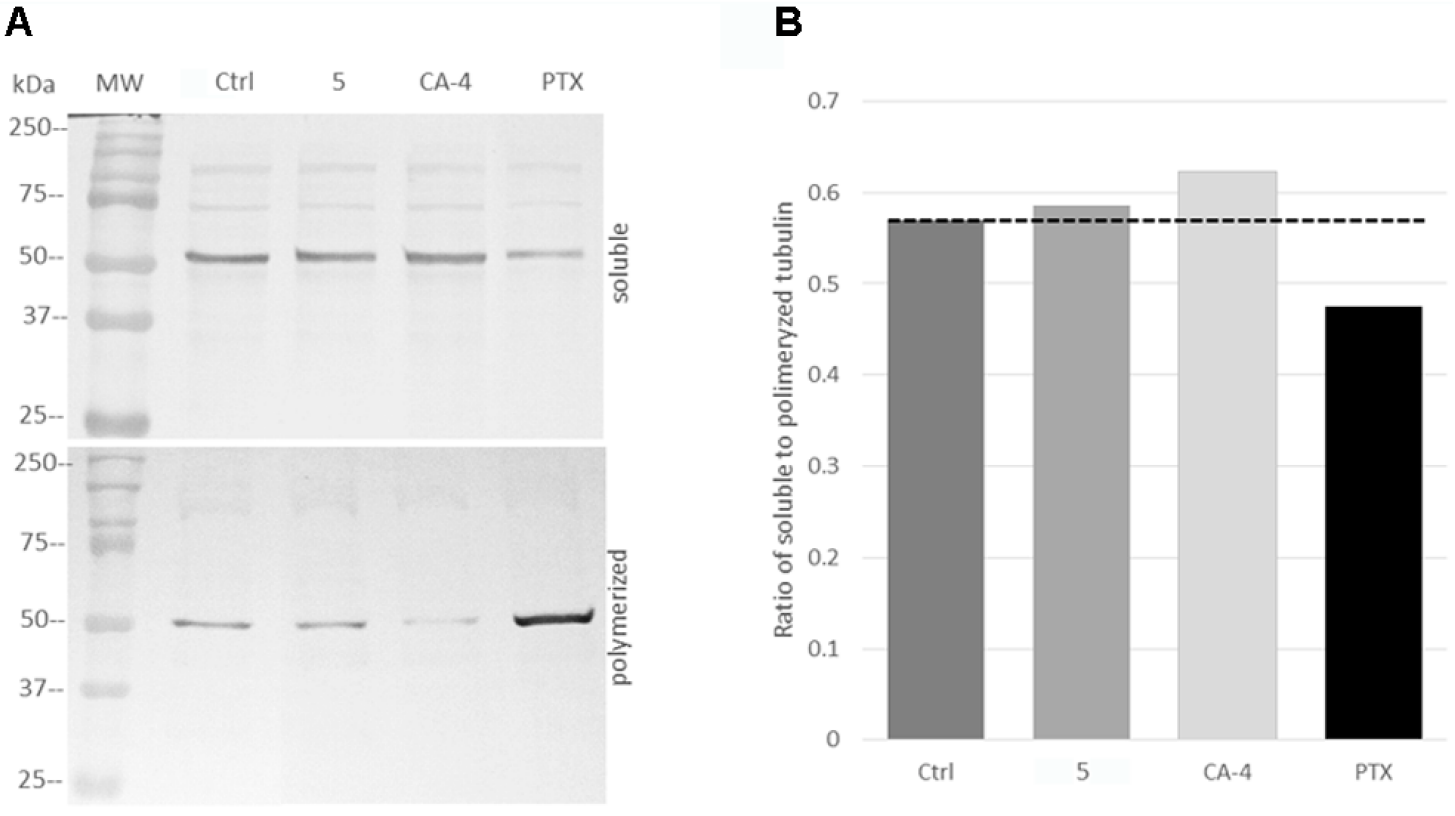
(**A**) Western blot analysis of soluble and polymerized tubulin fractions of cells without treatment (Ctrl) and cells treated with compound **5** (0.16 μM), **CA-4** (1.49 μM), and **PTX** (0.08 μM). (**B**) Ratio of soluble-to-polymerized tubulin of the data A. MW = molecular weight.

IFI assays showed that compound **5** induced alterations in the microtubule network. Then, to identify the mechanism by which compound **5** affects the stability of microtubules, soluble and polymerized tubulin protein obtained from treated HeLa cells were analyzed by Western blotting. **PTX** and **CA-4** were included as microtubule stabilizing and destabilizing agents, respectively, and DMSO as a negative control. In control and treated samples, bands of 50 kDa corresponding to beta-tubulin were detected (Figure 6A). Densitometric analysis showed that in cells treated with **CA-4** or compound **5**, the fraction of soluble tubulin increased in comparison to the control condition or **PTX** treatment. On the contrary, in cells treated with **PTX**, an increase of polymerized tubulin was evident. The ratio of soluble to polymerized tubulin fractions was determined for each compound and changes in the tubulin polymerization state following drug treatment were evidenced (Figure 6B). **PTX** showed a lower ratio of soluble-to-polymerized tubulin compared to control due to its stabilizing effect of the polymerized state of tubulin proteins. Meanwhile, **CA-4** and compound **5** displayed a higher soluble-to-polymerized tubulin ratio in comparison to control or **PTX**. This suggests that compound **5** exhibits an inhibitory activity of tubulin polymerization similar to that of **CA-4**. This inhibitory activity was indeed observed in the tubulin polymerization assay. 

## 3. Materials and Methods

### 3.1. Chemistry

All chemicals were obtained from Sigma-Aldrich (Sigma-Aldrich, Toluca, MEX, Mexico). Reactions were monitored by TLC on 0.2 mm percolated silica gel 60 F254 plates (Merck, Darmstadt, Germany) and visualized by irradiation with a UV lamp (Cole-Parmer, Vernon Hills, IL, USA). Silica gel 60 (70–230 mesh) was used for column chromatography (Macherey-Nagel, Düren, Germany). Melting points were determined in open capillary tubes with a Büchi M-565 melting point apparatus (Büchi, Flawil, Switzerland) and are uncorrected. Microwave-assisted reactions were carried out in a Monowave 300 monomodal reactor equipped with a hydraulic pressure sensing device and an infrared temperature-sensor (Anton-Paar, Graz, Austria). ^1^H NMR and ^13^C NMR were measured with an Agilent DD2 spectrometer (Agilent, Santa Clara, CA, USA), operating at 600 and 151 MHz for ^1^H and ^13^C, respectively. Chemical shifts are given in parts per million relatives to tetramethylsilane (Me_4_Si, δ = 0); *J* values are given in Hz. Splitting patterns are expressed as follow: s, singlet; d, doublet; t, triplet; q, quartet; dd, doublet of doublet; td, triplet of doublet; ddd, doublet doublet of doublet; dq, doublet of quartets; m, multiplet; bs, broad singlet. The purity of compounds 4, 5, and 6 was determined by quantitative NMR spectroscopy (qNMR) using sodium 2,2-dimethyl-2-silapentane-5-sulfonate (DSS) as internal standard (Cambridge Isotope Laboratories, Inc., Tewksbury, MA, USA). High resolution mass spectra were recorded on a Bruker ESI/APCI-TOF, MicroTOF-II-Focus spectrometer (Bruker, Billerica, MA, USA) by electrospray ionization (ESI); whereas low resolution Mass spectra were recorded on a Waters Xevo TQ-MS spectrometer (Waters, Milford, MA, USA) by electron impact (EI). 

#### 3.1.1. General Procedure for the Synthesis of 2-Phenyl-2*H*-indazole Derivatives

2-Phenyl-2*H*-indazole derivatives were synthesized employing a slight modification of the Cadogan method [11]. A 10 mL microwave vial was charged with 2-nitrobenzaldehyde (0.5 g, 3.308 mmol), aniline or the appropriate substituted aniline (3.308 mmol, 1 eq) and anhydrous ethanol (3 mL). The mixture was heated at 160 °C under microwave irradiation for 10 min. The mixture was transferred to a round bottom flask and distilled under vacuum. The evaporation residue was treated with triethyl phosphite (10 mmol) and heated at 150 °C (0.5–2 h) until the starting material was totally consumed. Then, the phosphite excess was oxidized with 20 mL of 5% hydrogen peroxide solution. The product was extracted with ethyl acetate (20 mL × 3) and the combined organic phase was finally washed with brine (20 mL) and dried with anhydrous sodium sulfate. The organic solution was refluxed for 10 min with activated carbon (200 mg), and filtered and concentrated under vacuum distillation. The evaporation residue was purified using column chromatography with silica and hexane-ethyl acetate (85:15) as a mobile phase, the combined fractions containing the product were concentrated to 10–20 mL under reduced pressure and cooled to induce crystallization. The solid was finally filtered and washed with cold hexane to give the product. 

##### 2-Phenyl-2*H*-indazole (**1**)

White solid, 56% yield, mp: 81–82 °C (lit: 81–82 °C) [11]. The spectroscopic data matched previously reported data [36]: ^1^H NMR (600 MHz, CDCl_3_) δ 8.40 (d, *J* = 0.9 Hz, 1H), 7.91–7.88 (m, 2H), 7.79 (dd, *J* = 8.8, 0.9 Hz, 1H), 7.70 (dt, *J* = 8.5, 1.0 Hz, 1H), 7.54–7.50 (m, 2H), 7.41–7.37 (m, 1H), 7.32 (ddd, *J* = 8.8, 6.6, 1.0 Hz, 1H), 7.11 (ddd, *J* = 8.4, 6.6, 0.7 Hz, 1H); ^13^C NMR (151 MHz, CDCl_3_) δ (ppm): 149.78, 140.52, 129.54, 127.88, 126.81, 122.76, 122.44, 120.99, 120.39, 120.37, 117.94.

##### 2-(3,4,5-Trimethoxyphenyl)-2*H*-indazole (**2**)

Pale yellow solid, 61% yield, mp: 94–95 (lit: 82–84 °C) [37]. The spectroscopic data matched previously reported data [37]: ^1^H NMR (600 MHz, CDCl_3_) δ 8.36 (d, *J* = 0.8 Hz, 1H), 7.78 (dt, *J* = 9.6, 0.8 Hz, 1H), 7.70 (dt, *J* = 8.3, 1.0 Hz, 1H), 7.33 (ddd, *J* = 8.8, 6.6, 1.0 Hz, 1H), 7.14–7.10 (m, 3H), 3.97 (s, 6H), 3.90 (s, 3H). ^13^C NMR (151 MHz, CDCl_3_) δ 153.78, 149.62, 137.83, 136.55, 126.82, 122.67, 122.45, 120.64, 120.26, 117.79, 98.93, 61.04, 56.39.

##### 5-(2*H*-Indazol-2-yl)-2-methoxyphenol (**3**)

White solid, 33% yield, mp: 143–144 °C. ^1^H NMR (600 MHz, CDCl_3_) δ 8.29 (d, *J* = 0.8 Hz, 1H), 7.80–7.76 (m, 1H), 7.71–7.67 (m, 1H), 7.46 (d, *J* = 2.6 Hz, 1H), 7.39 (dd, *J* = 8.7, 2.6 Hz, 1H), 7.31 (ddd, *J* = 8.7, 6.6, 1.0 Hz, 1H), 7.10 (ddd, *J* = 8.4, 6.5, 0.7 Hz, 1H), 6.94 (d, *J* = 8.6 Hz, 1H), 6.06 (s, 1H), 3.93 (s, 3H); ^13^C NMR (151 MHz, CDCl_3_) δ 149.53, 146.46, 146.33, 134.60, 126.60, 122.64, 122.25, 120.47, 120.27, 117.77, 112.72, 110.92, 108.17, 56.21; MS (HR-ESI) for C_14_H_13_N_2_O_2_ [M + H]+, calcd: *m*/*z* 241.0972, found: *m*/*z* 241.0970.

#### 3.1.2. General Procedure for the Synthesis of 3-Bromo-2-phenyl-2*H*-indazole Derivatives

2-Phenyl-2*H*-indazole (2 mmol), or the appropriate substituted derivative (2–3), was dissolved in acetic acid (10 mL) at room temperature. The formed solution was treated, drop to drop gradually during a period of 1 h at room temperature, with a second solution of bromine 2.1 mmol in 10 mL of acetic acid. The resulting solution was stirred overnight at room temperature. The mixture was treated with 2 mL of water and then quenched under 100 mL of iced water. The solid formed was filtered under vacuum and dried. The product was used crude for to the next reaction [12].

##### 3-Bromo-2-phenyl-2*H*-indazole (**1b**)

White solid, 86% yield, a sample was recrystallized from ethanol/water to give mp: 77–79 (lit: 75.5–77 °C) [12]. ^1^H NMR (600 MHz, CDCl_3_) δ 7.74 (dt, *J* = 8.8, 0.8 Hz, 1H), 7.70–7.66 (m, 2H), 7.59 (dt, *J* = 8.5, 1.0 Hz, 1H), 7.57–7.50 (m, 3H), 7.37 (ddd, *J* = 8.7, 6.6, 1.1 Hz, 1H), 7.18 (ddd, *J* = 8.4, 6.6, 0.7 Hz, 1H); ^13^C NMR (151 MHz, CDCl_3_) δ 149.20, 139.26, 129.23, 129.04, 127.58, 126.17, 122.97, 122.89, 119.68, 118.19, 106.13.

##### 3-Bromo-2-(3,4,5-trimethoxyphenyl)-2*H*-indazole (**2b**)

White solid, 91% yield, mp: 115–117. ^1^H NMR (600 MHz, CDCl_3_) δ 7.73 (dt, *J* = 8.7, 0.8 Hz, 1H), 7.58 (dt, *J* = 8.4, 1.0 Hz, 1H), 7.37 (ddd, *J* = 8.8, 6.6, 1.1 Hz, 1H), 7.19 (ddd, *J* = 8.4, 6.6, 0.7 Hz, 1H), 6.89 (s, 2H), 3.93 (s, 3H), 3.92 (s, 6H); ^13^C NMR (151 MHz, CDCl_3_) δ 153.24, 149.02, 138.70, 134.73, 127.64, 123.02, 122.80, 119.61, 118.06, 106.22, 103.93, 61.01, 56.35.

##### 3-Bromo-2-(3,4,5-trimethoxyphenyl)-2*H*-indazole (**3b**) 

White solid, 98% yield, mp: 118–121. ^1^H NMR (600 MHz, CDCl_3_) δ 7.72 (d, *J* = 8.8 Hz, 1H), 7.57 (dt, *J* = 8.4, 0.9 Hz, 1H), 7.35 (ddd, *J* = 8.7, 6.6, 1.0 Hz, 1H), 7.23 (d, *J* = 2.5 Hz, 1H), 7.18–7.13 (m, 2H), 6.97 (d, *J* = 8.6 Hz, 1H), 6.08 (s, 1H), 3.97 (s, 3H); ^13^C NMR (151 MHz, CDCl_3_) δ 148.90, 147.34, 145.76, 132.66, 127.46, 122.81, 122.64, 119.61, 118.03, 112.99, 110.26, 106.46, 56.16.

#### 3.1.3. General Procedure for Palladium-Catalyzed Coupling Reactions

A 10 mL microwave vial was charged with the properly substituted 3-bromo-2-phenyl-2*H*-indazole or 3-bromo-1*H*-indazole (0.6 mmol), the appropriate phenylboronic acid or phenylboronic acid pinacol ester (0.63 mmol), palladium (II) acetate (0.006 mmol, 1%), triphenylphosphine (0.018 mmol, 3%), sodium carbonate (1.2 mmol), water (0.6 mL), and *n*-propanol (3 mL). The mixture was heated at 150 °C under microwave irradiation for 20 min. The reaction was poured into 15 mL of water and extracted thrice with 15 mL of ethyl acetate. The organic phase was washed with brine (15 mL), dried with anhydrous sodium sulfate, and concentrated under vacuum. The evaporation residue was purified by column chromatography using hexane-ethyl acetate, 90:10 for compound **4** and 75:25 for compounds **5** and **6**.

##### 2,3-Diphenyl-2*H*-indazole (**4**)

White solid, 72% yield; mp: 107–108 °C (lit: 102–103 °C) [38]. The spectroscopic data matched previously reported data [8,38]: ^1^H NMR (600 MHz, CDCl_3_) δ 7.82–7.79 (m, 1H), 7.73–7.70 (m, 1H), 7.45–7.42 (m, 2H), 7.41–7.34 (m, 9H), 7.14 (ddd, *J* = 8.4, 6.6, 0.8 Hz, 1H); ^13^C NMR (151 MHz, CDCl_3_) δ 148.99, 140.24, 135.41, 129.91, 129.69, 128.97, 128.76, 128.30, 128.25, 126.98, 126.02, 122.50, 121.74, 120.52, 117.76; Purity (qNMR, % *w*/*w*) 98.01 ± 2.10.

##### 2-Methoxy-5-(2-(3,4,5-trimethoxyphenyl)-2*H*-indazol-3-yl)phenol (**5**)

White solid, 84% yield; mp: 160–162 °C; ^1^H NMR (600 MHz, CDCl_3_) δ 7.77 (dt, *J* = 8.8, 0.9 Hz, 1H), 7.69 (dt, *J* = 8.5, 1.0 Hz, 1H), 7.35 (ddd, *J* = 8.7, 6.6, 0.9 Hz, 1H), 7.12 (ddd, *J* = 8.5, 6.6, 0.8 Hz, 1H), 7.03 (d, *J* = 2.1 Hz, 1H), 6.88 (d, *J* = 8.4 Hz, 1H), 6.84 (dd, *J* = 8.3, 2.0 Hz, 1H), 6.68 (s, 2H), 5.84 (s, 1H), 3.92 (s, 3H), 3.87 (s, 3H), 3.72 (s, 6H); ^13^C NMR (151 MHz, CDCl_3_) δ 153.09, 148.68, 146.72, 145.78, 137.82, 135.82, 135.23, 127.00, 123.07, 122.24, 121.89, 121.60, 120.58, 117.46, 115.69, 110.78, 103.61, 60.98, 56.13, 55.98; MS (HR-ESI) for C_23_H_23_N_2_O_5_ [M + H]+, calcd: *m*/*z* 407.1601, found: *m*/*z* 407.1595; Purity (qNMR, % *w*/*w*) 98.46 ± 1.7.

##### 2-Methoxy-5-(3-(3,4,5-trimethoxyphenyl)-2*H*-indazol-2-yl)phenol (**6**)

White solid, 68% yield, mp: 192–194 °C; ^1^H NMR (600 MHz, CDCl_3_) δ 7.78 (dt, *J* = 8.8, 0.9 Hz, 1H), 7.74 (dt, *J* = 8.5, 1.0 Hz, 1H), 7.36 (ddd, *J* = 8.7, 6.6, 1.0 Hz, 1H), 7.15 (ddd, *J* = 8.4, 6.5, 0.7 Hz, 1H), 7.10 (d, *J* = 2.4 Hz, 1H), 6.90 (dd, *J* = 8.6, 2.5 Hz, 1H), 6.83 (d, *J* = 8.6 Hz, 1H), 6.58 (s, 2H), 5.97 (s, 1H), 3.92 (s, 3H), 3.90 (s, 3H), 3.73 (s, 6H); ^13^C NMR (151 MHz, CDCl_3_) δ 153.34, 148.65, 146.71, 145.87, 138.11, 135.36, 133.90, 126.86, 125.15, 122.41, 121.25, 120.39, 117.93, 117.76, 112.95, 110.28, 106.95, 60.98, 56.20, 56.14; MS (EI) *m*/*z* 407.25 ([M + H]+, 100%); Purity (qNMR, % *w*/*w*) 96.65 ± 1.43.

##### 3-Phenyl-1*H*-indazole (**7**) 

Light yellow solid, 80% yield, mp: 105–107 °C (lit: 106‒107) [39]. The spectroscopic data matched previously reported data [40]: ^1^H NMR (600 MHz, CDCl_3_) δ 11.44 (s, 1H), 8.05–7.99 (m, 3H), 7.56–7.51 (m, 2H), 7.47–7.42 (m, 1H), 7.37–7.33 (m, 1H), 7.29–7.25 (m, 1H), 7.23–7.19 (m, 1H); ^13^C NMR (151 MHz, CDCl_3_) δ 145.74, 141.68, 133.56, 128.93, 128.17, 127.72, 126.78, 121.34, 121.09, 120.96, 110.22.

##### 3-(3,4,5-Trimethoxyphenyl)-1*H*-indazole (**8**) 

Yellow oil, 94% yield; ^1^H NMR (600 MHz, CDCl_3_) δ 11.04 (bs, 1H), 8.01 (dt, *J* = 8.2, 0.9 Hz, 1H), 7.43–7.38 (m, 2H), 7.26–7.23 (m, 1H), 7.22 (s, 2H), 3.94 (2s, *J* = 0.5 Hz, 9H); ^13^C NMR (151 MHz, CDCl_3_) δ 153.67, 145.73, 141.74, 138.27, 129.12, 126.95, 121.49, 120.97, 120.86, 110.23, 104.87, 61.02, 56.26; MS (EI) *m*/*z* 285.20 ([M + H]+, 100%).

##### 5-(1*H*-Indazol-3-yl)-2-methoxyphenol (**9**) 

White solid, 85% yield, mp: 190–194 °C; ^1^H NMR (600 MHz, DMSO-*d*_6_) δ 13.06 (s, 1H), 9.18 (s, 1H), 7.99 (d, *J* = 8.2 Hz, 1H), 7.56 (d, *J* = 8.4 Hz, 1H), 7.46 (d, *J* = 2.1 Hz, 1H), 7.41–7.36 (m, 2H), 7.18 (t, *J* = 7.7 Hz, 1H), 7.05 (d, *J* = 8.3 Hz, 1H), 3.83 (s, 3H); ^13^C NMR (151 MHz, DMSO-*d*_6_) δ 147.44, 146.67, 143.12, 141.42, 126.64, 125.83, 120.57, 120.53, 119.87, 117.70, 113.91, 112.45, 110.37, 55.56; MS (EI) *m*/*z* 241.14 ([M + H]+, 100%).

### 3.2. Cytotoxicity Assays in Human Cells

HeLa (derived from human epithelial cervical cancer, ATCC 93021013), SK-LU-1 (derived from human non-small lung adenocarcinoma ATCC 93120835), and K562 (derived from a patient in the blast crisis stage of chronic myelogenous leukemia ATCC 93112521) cancer cell lines were chosen to test the cytotoxicity of all compounds. Cancer cell lines were kindly provided by Dr. Marco A. Cerbón, Laboratory of Molecular Endocrinology, Facultad de Química UNAM. 

HeLa, SK-LU-1, and K562 cell lines were grown in DMEM (BioWest, Riverside, MO, USA) supplemented with 10% FBS (BioWest, Riverside, MO, USA) and maintained in standard culture conditions (37 °C, 95% humidity, and 5% CO_2_). Cells were grown to a density of 80% and then were harvested using sterile PBS/EDTA (pH 7.4) before starting every experiment. Cells were seeded in 96-well plates (7 × 10^3^ cells/well in 200 µL of DMEM) and maintained 24 h in standard conditions. Afterwards, cells were exposed during 48 h to tested compounds dissolved in DMSO (J.T. Baker, Phillipsburg, NJ) at different concentrations and diluted in 50 µL of DMEM, to reach 250 µL in the well. After treatment, cellular viability was analyzed by MTT assay, the absorbance of formazan was determined using a microplate reader Epoch 2 (BioTek, Winooski, VT, USA) at 540 nm and the viability for each concentration was related to the vehicle (100%). The IC_50_ was calculated from dose-response curve by non-linear fit using the Software OriginPro 7.0 (RockWare, Golden, CO, USA) [8,41,42].

### 3.3. Molecular Docking 

Docking studies were performed using AutoDock 4.2, whereas the graphical interface AutoDockTools 1.7.1 suite was used to prepare and analyze the docking simulations [24]. The X-Ray structure of tubulin-cis-**CA-4** complex was retrieved from Protein Data Bank (http://www.rcsb.org/ (accessed on 2 February 2019)) entry 5LYJ [25,43,44]. First, chains A and B were selected and the solvent molecules as well as **CA-4** were removed. The pdb structure was submitted for minimization using the YASARA web server (YASARA, Vienna, Austria) [45]. The minimized protein was then exported to AutoDockTools interface 1.7.1. Hydrogen atoms were added and Gasteiger-Marsilli charges were assigned to the atoms into the protein as well. The chemical structures of **CA-4**, colchicine and indazole were retrieved from ZINC database [46]. Ligands were built by systematic modifications of indazole and then energy-minimized by semiempirical PM6 method using Gaussian 09 software [47]. Docking simulations were performed using a grid box of size 50 Å × 50 Å × 50 Å with a spacing of 0.345 Å focused on the binding region of **CA-4** (coordinates: x = 17.85, y = 65.75 and z = 42.59). The search was carried out with the Lamarckian Genetic Algorithm. A total of 50 GA runs with a maximal number of 25,000,000 evaluations, a mutation rate of 0.02 and an initial population of 150 conformers were covered. Docking results were clustered using a root mean square (RMS) tolerance = 1. Finally, each ligand with the best cluster size and the lowest binding free energy was further selected for the analysis. The docking protocol was validated by comparison of docked **CA-4** and the co-crystalized **CA-4**. The ligand interactions were analyzed using AutoDockTools 1.7.1 interface along with BIOVIA Discovery Studio Visualizer 2016 [48]. Figures and 2D diagrams were produced with UCSF Chimera program [49], and BIOVIA Discovery Studio Visualizer 2016. 

### 3.4. Tubulin Polymerization Assays

The in vitro tubulin polymerization assays were performed using the kit manufactured by Cytoskeleton, Inc., catalog number BK006P (Cytoskeleton, Denver, CO, USA). Assays were carried out following the instructions provided by the manufacturer [26,27,28]. Briefly, compounds **5**, **6**, and references (**PTX** and **CA-4**) were dissolved in DMSO and tested at 10 µM. The tubulin polymerization assay was conducted at 37 °C, recording the change in absorbance every minute at 340 nm after test compound was added. 

### 3.5. Indirect Immunofluorescence Assays

HeLa cells (4 × 10^5^) were seeded on coverslips in 30 mm petri dishes and incubated in DMEM with 10% of fetal bovine serum (FBS) for 24 h with compound **5** (0.16 μM). DMSO (0.01%) was included as negative control and **PTX** (0.08 μM) and **CA-4** (1.49 μM) as positive controls. After removal of the supernatant, the cells were fixed with 4% of paraformaldehyde in phosphate-buffered saline 1× (PBS) pH 7.4 for 1 h at 4 °C and permeabilized with 0.5% Triton X-100-SDS for 10 min. Nonspecific binding was blocked with 3% bovine serum albumin (BSA) in PBS for 1 h at 37 °C. Afterwards, treated cells were incubated with a monoclonal antibody anti-beta-tubulin (Sigma-Aldrich, T0198) diluted 1:1000 in PBS for 1 h at room temperature and washed three times with PBS; subsequently incubation with secondary anti-mouse-FITC antibody (Millipore, AP308F) diluted 1:500 and 4′,6′-diamidino-2-phenylindole (DAPI) at 0.5 µg/mL was carried out for 1 h at room temperature in the dark. The cover slips were washed three times with PBS and mounted on glass slides with Vectashield (Vector Laboratories). Images were acquired on a Nikon Ti Eclipse inverted confocal microscope equipped with an A1 imaging system. Imaging was performed using a 20× (dry, NA = 0.8) objective lens. Excitation was performed sequentially using the laser lines provided by the manufacturer (403 nm—DAPI, 488 nm—FITC, brightfield) and the appropriate emission filters.

### 3.6. Cell Cycle Analysis

Cell cycle progression was monitored by flow cytometry using propidium iodide (PI, Sigma, St. Louis, MO, USA). For this, HeLa cells were treated for 48 h with compound **5** (0.16 μM) and **CA-4** (1.49 μM). Afterwards, cells were collected by centrifugation, washed with PBS, and fixed with ice-cold 75% ethanol. The fixed cells were harvested by centrifugation at 1400 rpm for 5 min and resuspended in 500 μL of PBS, and treated with RNase A (100 μg/mL, 30 min, 37 °C). After incubation, the cells were stained with PI at 10 μg/mL at 37 °C for 30 min. 

### 3.7. Western Blot Analysis of Soluble and Polymerized Tubulin in HeLa cells

HeLa cells (1.65 × 10^6^ cells) cultured in DMEM medium complemented with FBS (10%) were seeded in 60 mm petri dishes and treated with compound **5** at 0.16 µM for 24 h at 37 °C. **PTX** (0.08 µM) and **CA-4** (1.49 µM) were included as positive controls and DMSO (0.01%) as negative control. Soluble and polymerized tubulin were obtained as previously recommended [50]. To collect soluble fraction, cells were permeabilized with 300 µL lysis buffer (80 mM Pipes-KOH (pH 6.8), 1 mM MgCl_2_, 1mM EGTA, 0.2% Triton X-100, 10% glycerol, 0.1% protease inhibitor cocktail (Sigma-Aldrich)) for 3 min at 30 °C. Afterwards, the soluble tubulin contained in the supernatant was gently removed. Polymerized tubulin (insoluble fraction) was obtained in the pellet with 200 µL of Laemmli’s buffer (180 mM, Tris-HCl (pH 6.8), 6% SDS, 15% glycerol, 7.5% β-mercaptoethanol and 0.01% of bromophenol blue) which was heated for 3 min at 95 °C. Protein concentration was determined by 2-D Quant kit (GE Healthcare). Fractions containing soluble and polymerized tubulin were run on SDS-12% polyacrylamide gel for 1 h 30 min at 100 V and transferred to a nitrocellulose membrane (Bio-Rad, Hercules, CA, USA). The membrane was blocked with 3% BSA in PBS overnight at 4 °C and further incubated with an anti-beta-tubulin monoclonal antibody (Sigma-Aldrich, T0198) at 1:1000 dilution for 1 h 30 min at 37 °C. After this, the membrane was washed 3× with PBS and incubated with goat anti-mouse IgG- horseradish peroxidase conjugated as secondary antibody (Millipore, AP308P) (1:1000 dilution) for 1 h 30 min at 37 °C (Merck Millipore, Burlington, MA, USA). Bands were revealed with 4-chloro-1-naphtol. Images were captured with a photodocumenter (UVITEC, Cambridge, UK) and intensity of each band was analyzed with Quantity One 4.6 software (Bio-Rad, Hercules, CA, USA).

### 3.8. Antioxidant Evaluation

#### 3.8.1. DPPH Assay

In a 96-well plate, 200 µL of an ethanolic solution of DPPH (0.208 mM) and 100 µL of the compound solution at different concentrations (10, 50, 100, 200, and 300 µM) were added. Ethanol was used as a negative control, while Trolox was used as positive control. The plates were incubated for 30 min in dark. Finally, the DPPH discoloration was measured by absorbance in a microplate reader Epoch 2 (BioTek, Winooski, VT, USA) at 515 nm. The IC_50_ values were calculated from the dose–response curve by non-linear fit [51].

#### 3.8.2. ABTS Assay

The determinations were carried out employing a slight modification of the previously reported method [52]. Briefly, stock solutions of ABTS at 7 mM and potassium persulfate at 2.45 mM were mixed in equal proportions. The resulted solution was stored for 12 h at room temperature in dark. Then, in a 96-well plate, 20 µL of the compound solution (at concentrations of 10, 50, 100, 200 and 300 µM) and 180 µL the reactive solution were added. Ethanol was used as a negative control, while Trolox was used as positive control. Plates were incubated for 10 min in dark. Then, the absorbance was measured in a microplate reader Epoch 2 (BioTek, Winooski, VT, USA) at 734 nm. The IC_50_ values were calculated from the dose–response curve by non-linear fit [51].

## 4. Conclusions

Two hybrid compounds of **CA-4** and 2,3-diphenyl-2*H*-indazole (**5** and **6**) and seven related derivatives were synthesized with microwave assisted chemistry showing short reaction times and good yields. The seven derivative compounds were designed and synthesized to gain knowledge about the importance of different substituents on the cytotoxic activity. Biological evaluation revealed that hybrid compounds **5** and **6** have strong cytotoxic activity against HeLa, SK-LU-1, and K562 cancer cell lines. The 3,4,5-trimethoxyphenyl and 4-methoxy-3-hydroxyphenyl groups at positions 2 and 3 of the indazole nucleus were essential to the observed cytotoxic effect, at least on the three cell lines tested. Compound **5** possess the best cytotoxic effect on HeLa, SK-LU-1, and K562 cell lines as compared with **4** and **6**. Additionally, its cytotoxic effect is greater than the reference drug cisplatin against HeLa and SK-LU-1 cells and similar in potency than the reference drug imatinib against K562 cells. Molecular docking studies and in vitro tubulin polymerization assay strongly suggest tubulin as a molecular target for compound **5**; however, an additional action mechanism for compound **5** can be also involved. Taken altogether, data demonstrated that compound **5** is a potent agent that induces G_2_/M arrest via disrupting the microtubule network that deserves further research and, importantly, it provides information for exploring new **CA-4** analogues.

## Figures and Tables

**Table 1:** Cytotoxicity (IC_50_ [µM] ± SE) for compounds **5** and **6** and reference compounds **4**, cisplatin, and imatinib on different cancer cell lines.

Compound	HeLa	SK-LU-1	K562
**4**	125.8 ± 14.8 ^1^	96.43 ± 8.65	> 120
**5**	0.16 ± 0.01	6.63 ± 0.52	1.68 ± 0.27
**6**	38.6 ± 7.51	25.21 ± 9.06	18.59 ± 1.56
Cisplatin	18.5 ± 3.0	12.0 ± 2.5	-
Imatinib	-	-	2.37 ± 0.12

^1^ Reference [8].

## Data Availability

Data is contained within the article and supplementary material.

## Supplementary Materials

The following are available online at https://www.mdpi.com/article/10.3390/ph14080815/s1, Table S1: Palladium-catalyzed arylation of 3-bromophenylindazoles and microwave assisted modification, Table S2: Cellular viability ± SE on HeLa cell line upon treatment with compounds **1**–**9** at 50 µM, Table S3: Antioxidant activity (IC_50_ [µM]) of compounds **4**–**6** employing DPPH and ABTS assays, Table S4: Purity of compounds **4**, **5**, and **6** by qNMR used an internal standard (%purity, mean ± standard deviation), Figures S1–S29: ^1^H NMR; ^13^C NMR, and MS spectra for synthesized compounds, Figure S30: Morphological changes of HeLa cells after 48 h of treatment, Figure S31: Quantitative apoptosis assay of HeLa using Annexin V-FITC/Ghost-Red.

## References

[1] National Cancer Institute What Is Cancer?. https://training.seer.cancer.gov/disease/cancer/review.html.

[2] WHO (World Health Organization). http://www.who.int/cancer/en/.

[3] Mansoori B., Mohammadi A., Davudian S., Shirjang S., Baradaran B. (2017). The Different Mechanisms of Cancer Drug Resistance: A Brief Review. Adv. Pharm. Bull..

[4] Thangadurai A., Minu M., Wakode S., Agrawal S., Narasimhan B. (2011). Indazole: A medicinally important heterocyclic moiety. Med. Chem. Res..

[5] Gaikwad D.D., Chapolikar A.D., Devkate C.G., Warad K.D., Tayade A.P., Pawar R.P., Domb A.J. (2015). Synthesis of indazole motifs and their medicinal importance: An overview. Eur. J. Med. Chem..

[6] Dong J., Zhang Q., Wang Z., Huang G., Li S. (2018). Recent Advances in the Development of Indazole-based Anticancer Agents. ChemMedChem.

[7] Zhang S.-G., Liang C.-G., Zhang W.-H. (2018). Recent Advances in Indazole-Containing Derivatives: Synthesis and Biological Perspectives. Molecules.

[8] Pérez-Villanueva J., Yépez-Mulia L., González-Sánchez I., Palacios-Espinosa J.F., Soria-Arteche O., Sainz-Espuñes T.D.R., Cerbón M.A., Rodríguez-Villar K., Rodríguez-Vicente A.K., Cortés-Gines M. (2017). Synthesis and Biological Evaluation of 2H-Indazole Derivatives: Towards Antimicrobial and Anti-Inflammatory Dual Agents. Molecules.

[9] Lu Y., Chen J., Xiao M., Li W., Miller D.D. (2012). An Overview of Tubulin Inhibitors That Interact with the Colchicine Binding Site. Pharm. Res..

[10] Tron G.C., Pirali T., Sorba G., Pagliai F., Busacca A.S., Genazzani A. (2006). Medicinal Chemistry of Combretastatin A4: Present and Future Directions. J. Med. Chem..

[11] Cadogan J.I.G., Mackie R.K. (1968). 2-Phenylindazole. Org. Synth..

[12] Waalwijk P.S., Cohen-Fernandes P., Habraken C.L. (1984). Indazole studies. 3. The bromination of 2-phenyl-2H-indazole. Formation and structure determination of mono-, di-, and tribromo-2-phenyl-2H-indazoles. J. Org. Chem..

[13] Huff B.E., Koenig T.M., Mitchell D., Staszak M.A. (2004). Synthesis of unsymmetrical biaryls using a modified Suzuki cross-coupling: 4-Biphenylcarboxaldehyde. ([1,1′-Biphenyl]-4-carboxaldehyde). Org. Synth..

[14] Wehbe H., Kearney C.M., Pinney K.G. (2005). Combretastatin A-4 resistance in H460 human lung carcinoma demonstrates distinctive alterations in beta-tubulin isotype expression. Anticancer. Res..

[15] Duan Y.-T., Man R.-J., Tang D.-J., Yao Y.-F., Tao X.-X., Yu C., Liang X.-Y., Makawana J.A., Zou M.-J., Wang Z.-C. (2016). Design, Synthesis and Antitumor Activity of Novel link-bridge and B-Ring Modified Combretastatin A-4 (CA-4) Analogues as Potent Antitubulin Agents. Sci. Rep..

[16] Ma M., Sun L., Lou H., Ji M. (2013). Synthesis and biological evaluation of Combretastatin A-4 derivatives containing a 3’-O-substituted carbonic ether moiety as potential antitumor agents. Chem. Central J..

[17] Greene L.M., Nathwani S.M., Bright S.A., Fayne D., Croke A., Gagliardi M., McElligott A., O’Connor L., Carr M., Keely N.O. (2010). The Vascular Targeting Agent Combretastatin-A4 and a Novel cis-Restricted β-Lactam Analogue, CA-432, Induce Apoptosis in Human Chronic Myeloid Leukemia Cells and Ex Vivo Patient Samples Including Those Displaying Multidrug Resistance. J. Pharmacol. Exp. Ther..

[18] Seddigi Z.S., Malik M.S., Saraswati A.P., Ahmed S.A., Babalghith A.O., Lamfon H.A., Kamal A. (2017). Recent advances in combretastatin based derivatives and prodrugs as antimitotic agents. MedChemComm.

[19] Dasari S., Tchounwou P.B. (2014). Cisplatin in cancer therapy: Molecular mechanisms of action. Eur. J. Pharmacol..

[20] Moen M.D., McKeage K., Plosker G.L., Siddiqui M.A. (2007). Imatinib: A review of its use in chronic myeloid leukaemia. Drugs.

[21] Gaulton A., Bellis L., Bento A.P.S.F.F., Chambers J., Davies M., Hersey A., Light Y., McGlinchey S., Michalovich D., Al-Lazikani B. (2011). ChEMBL: A large-scale bioactivity database for drug discovery. Nucleic Acids Res..

[22] Bruggisser R., von Daeniken K., Jundt G., Schaffner W., Tullberg-Reinert H. (2002). Interference of Plant Extracts, Phytoestrogens and Antioxidants with the MTT Tetrazolium Assay. Planta Med..

[23] Wang P., Henning S.M., Heber D. (2010). Limitations of MTT and MTS-Based Assays for Measurement of Antiproliferative Activity of Green Tea Polyphenols. PLoS ONE.

[24] Morris G., Huey R., Lindstrom W., Sanner M.F., Belew R.K., Goodsell D.S., Olson A.J. (2009). AutoDock4 and AutoDockTools4: Automated docking with selective receptor flexibility. J. Comput. Chem..

[25] Gaspari R., Prota A.E., Bargsten K., Cavalli A., Steinmetz M.O. (2017). Structural Basis of cis- and trans-Combretastatin Binding to Tubulin. Chem.

[26] Cytoskeleton Inc Tubulin Polymerization Assay Using >99% Pure Tubulin, od Based—Porcine (BK006P). Manual: Tubulin Polymerization Assay Kit. https://www.cytoskeleton.com/bk006p.

[27] Shelanski M.L., Gaskin F., Cantor C.R. (1973). Microtubule Assembly in the Absence of Added Nucleotides. Proc. Natl. Acad. Sci. USA.

[28] Lee J.C., Timasheff S.N. (1977). In Vitro Reconstitution of Calf Brain Microtubules: Effects of Solution Variables. Biochemistry.

[29] Romagnoli R., Baraldi P.G., Brancale A., Ricci A., Hamel E., Bortolozzi R., Basso G., Viola G. (2011). Convergent synthesis and biological evaluation of 2-amino-4-(3′,4′,5′-trimethoxyphenyl)-5-aryl thiazoles as microtubule targeting agents. J. Med. Chem..

[30] Liang W., Lai Y., Zhu M., Huang S., Feng W., Gu X. (2016). Combretastatin A4 Regulates Proliferation, Migration, Invasion, and Apoptosis of Thyroid Cancer Cells via PI3K/Akt Signaling Pathway. Med Sci. Monit..

[31] Zhu H., Zhang J., Xue N., Hu Y., Yang B., He Q. (2010). Novel combretastatin A-4 derivative XN0502 induces cell cycle arrest and apoptosis in A549 cells. Investig. N. Drugs.

[32] Kanthou C., Greco O., Stratford A., Cook I., Knight R., Benzakour O., Tozer G. (2004). The Tubulin-Binding Agent Combretastatin A-4-Phosphate Arrests Endothelial Cells in Mitosis and Induces Mitotic Cell Death. Am. J. Pathol..

[33] Zacharaki P., Stephanou G., Demopoulos N.A. (2012). Comparison of the aneugenic properties of nocodazole, paclitaxel and griseofulvinin vitro. Centrosome defects and alterations in protein expression profiles. J. Appl. Toxicol..

[34] Sun Y., Lin X., Chang H. (2016). Proliferation inhibition and apoptosis of breast cancer MCF-7 cells under the influence of colchicine. J. BUON Off. J. Balk. Union Oncol..

[35] Ibrahim T.S., Hawwas M.M., Malebari A.M., Taher E.S., Omar A.M., Neamatallah T., Abdel-Samii Z.K., Safo M.K., Elshaier Y.A.M.M. (2021). Discovery of novel quinoline-based analogues of combretastatin A-4 as tubulin polymerisation inhibitors with apoptosis inducing activity and potent anticancer effect. J. Enzym. Inhib. Med. Chem..

[36] Fang Y., Wu C., LaRock R.C., Shi F. (2011). Synthesis of 2H-Indazoles by the [3 + 2] Dipolar Cycloaddition of Sydnones with Arynes. J. Org. Chem..

[37] Vidyacharan S., Sagar A., Chaitra N.C., Sharada D.S. (2014). A facile synthesis of 2H-indazoles under neat conditions and further transformation into aza-γ-carboline alkaloid analogues in a tandem one-pot fashion. RSC Adv..

[38] Ohnmacht S.A., Culshaw A.J., Greaney M.F. (2009). Direct Arylations of 2H-Indazoles On Water. Org. Lett..

[39] Croce P.D., La Rosa C. (1984). A Convenient synthesis of indazoles. Synthesis.

[40] Jin T., Yamamoto Y. (2007). An Efficient, Facile, and General Synthesis of 1H-Indazoles by 1,3-Dipolar Cycloaddition of Arynes with Diazomethane Derivatives. Angew. Chem. Int. Ed..

[41] Quintero A., Pelcastre A., Solano J.D. (1999). Antitumoral activity of new pyrimidine derivatives of sesquiterpene lactones. J. Pharm. Sci..

[42] Loza-Mejía M.A., Olvera-Vázquez S., Maldonado-Hernández K., Guadarrama-Salgado T., González-Sánchez I., Rodríguez-Hernández F., Solano J.D., Rodríguez-Sotres R., Lira-Rocha A. (2009). Synthesis, cytotoxic activity, DNA topoisomerase-II inhibition, molecular modeling and structure–activity relationship of 9-anilinothiazolo[5,4-b]quinoline derivatives. Bioorg. Med. Chem..

[43] Berman H., Henrick K., Nakamura H. (2003). Announcing the worldwide Protein Data Bank. Nat. Struct. Mol. Biol..

[44] Berman H.M., Westbrook J.D., Feng Z., Gilliland G., Bhat T.N., Weissig H., Shindyalov I.N., Bourne P.E. (2000). The Protein Data Bank. Nucleic Acids Res..

[45] Krieger E., Joo K., Lee J., Lee J., Raman S., Thompson J., Tyka M., Baker D., Karplus K. (2009). Improving physical realism, stereochemistry, and side-chain accuracy in homology modeling: Four approaches that performed well in CASP8. Proteins Struct. Funct. Bioinform..

[46] Irwin J., Shoichet B.K. (2004). ZINC—A Free Database of Commercially Available Compounds for Virtual Screening. J. Chem. Inf. Model..

[47] Frisch M.J.T.G.W., Schlegel H.B., Scuseria G.E., Robb M.A., Cheeseman J.R., Scalmani G., Barone V., Mennucci B., Petersson G.A., Nakatsuji H. (2009). Gaussian 09.

[48] (2016). Dassault Systèmes BIOVIA Discovery Studio.

[49] Pettersen E.F., Goddard T.D., Huang C.C., Couch G.S., Greenblatt D.M., Meng E.C., Ferrin T.E. (2004). UCSF Chimera—A visualization system for exploratory research and analysis. J. Comput. Chem..

[50] Kamal A., Shaik A.B., Polepalli S., Reddy V.S., Kumar G.B., Gupta S., Krishna K.V.S.R., Nagabhushana A., Mishra R.K., Jain N. (2014). Pyrazole–Oxadiazole conjugates: Synthesis, antiproliferative activity and inhibition of tubulin polymerization. Org. Biomol. Chem..

[51] Palacios-Espinosa J.F., Arroyo-García O., García-Valencia G., Linares E., Bye R., Romero I. (2014). Evidence of the anti-Helicobacter pylori, gastroprotective and anti-inflammatory activities of Cuphea aequipetala infusion. J. Ethnopharmacol..

[52] Thaipong K., Boonprakob U., Crosby K., Cisneros-Zevallos L., Byrne D.H. (2006). Comparison of ABTS, DPPH, FRAP, and ORAC assays for estimating antioxidant activity from guava fruit extracts. J. Food Compos. Anal..

