# Supplementary material for: Synthesis and Cytotoxic Activity of Combretastatin A-4 and 2,3-Diphenyl-2H-indazole Hybrids
Source: Pharmaceuticals (Basel). 2021 Aug 19;14(8):815. doi: 10.3390/ph14080815 (PMC8401203; doi:10.3390/ph14080815)
Supplement: Supplementary file 1 [file pharmaceuticals-14-00815-s001.zip › pharmaceuticals-1236818-supplementary.pdf]

pharmaceuticals

MDPI

Supporting Information

Synthesis and Cytotoxic Activity of Combretastatin A-4 and 2,3-Diphenyl-2*H*-indazole Hybrids

Jaime Pérez-Villanueva [1,*], Félix Matadamas-Martínez [1,2], Lilián Yépez-Mulia [3,*], Vadim Pérez-Koldenkova [4], Martha Leyte-Lugo [5], Karen Rodríguez-Villar [6], Francisco Cortés-Benítez [1], Ana Perla Macías-Jiménez [1], Juan Francisco Palacios-Espinosa [1], Ignacio González-Sánchez [5], Ariana Romero-Velásquez [7], Olivia Soria-Arteche [1]

[1] Departamento de Sistemas Biológicos, División de Ciencias Biológicas y de la Salud, Universidad Autónoma Metropolitana-Xochimilco (UAM-X), Ciudad de México 04960, Mexico; felixmatadamas@yahoo.com.mx (F.M.-M.); jcortesb@correo.xoc.uam.mx (F.C.-B.); anmacp61@gmail.com (A.P.M.-J.); jpalacios@correo.xoc.uam.mx (J.F.P.-E.); soriao@correo.xoc.uam.mx (O.S.-A.).

[2] Maestría y Doctorado en Ciencias Farmacéuticas, División de Ciencias Biológicas y de la Salud, Universidad Autónoma Metropolitana-Xochimilco (UAM-X), Ciudad de México 04960, Mexico; felixmatadamas@yahoo.com.mx (F.M.-M.).

[3] Unidad de Investigación Médica en Enfermedades Infecciosas y Parasitarias, UMAE Hospital de Pediatría, Centro Médico Siglo XXI, Instituto Mexicano del Seguro Social, Ciudad de México 06720, Mexico; lilianyepez@yahoo.com (L.Y.-M.)

[4] Laboratorio Nacional de Microscopía Avanzada, Centro Médico Nacional Siglo XXI, Instituto Mexicano del Seguro Social, Ciudad de México 06720, Mexico; vadim.perez@imss.gob.mx (V.P.-K.).

[5] Catedrático CONACYT comisionado a Departamento de Sistemas Biológicos, División de Ciencias Biológicas y de la Salud, Universidad Autónoma Metropolitana-Xochimilco (UAM-X), Ciudad de México 04960, Mexico; mailto:mleyte@correo.xoc.uam.mx (M.L.-L.); ignacio.gonzalez.s@gmail.com (I.G.-Z.).

[6] Doctorado en Ciencias Biológicas y de la Salud, Universidad Autónoma Metropolitana (UAM), Ciudad de México 04960, Mexico; qkarenrodv@hotmail.com (K.R.-V)

[7] Maestría en Ciencias Biológicas, Universidad Nacional Autónoma de México (UNAM), Ciudad de México 04510, Mexico; ariromerov@gmail.com (A.R.-V.)

* Correspondence: jpvillanueva@correo.xoc.uam.mx (J.P.-V.); Tel.: +52-5-54-83-72-59; Fax: +52-5-55-94-79-29; lilianyepez@yahoo.com (L.Y.-M.).

Table of Contents:

Contents	Page
**Table S1**. Palladium-catalyzed arylation of 3-bromophenylindazoles and microwave assisted modification	S4
**Table S2**. Cellular viability ± SE on HeLa cell line upon treatment with compounds **1–9** at 50 μM	S5
**Table S3**. Antioxidant activity (IC_{50} μM) of compounds **4–6** employing DPPH and ABTS assays.	S6
**Table S4.** Purity of compounds **4**, **5**, and **6** by qNMR used an internal standard (%purity, mean± standard deviation).	S7
**Figure S1**. ^{1}H NMR (600 MHz, $CDCl_3$) for 2-phenyl-2*H*-indazole (**1**).	S8
**Figure S2**. ^{13}C NMR (151 MHz, $CDCl_3$) for 2-phenyl-2*H*-indazole (**1**).	S9
**Figure S3**. ^{1}H NMR (600 MHz, $CDCl_3$) for 2-(3,4,5-trimethoxyphenyl)-2*H*-indazole (**2**).	S10
**Figure S4**. ^{13}C NMR (151 MHz, $CDCl_3$) for 2-(3,4,5-trimethoxyphenyl)-2*H*-indazole (**2**).	S11
**Figure S5**. ^{1}H NMR (600 MHz, $CDCl_3$) for 5-(2*H*-indazol-2-yl)-2-methoxyphenol (**3**).	S12
**Figure S6**. ^{13}C NMR (151 MHz, $CDCl_3$) for 5-(2*H*-indazol-2-yl)-2-methoxyphenol (**3**).	S13
**Figure S7**. MS (HR-ESI) for 5-(2*H*-indazol-2-yl)-2-methoxyphenol (**3**).	S14
**Figure S8**. ^{1}H NMR (600 MHz, $CDCl_3$) for 3-bromo-2-phenyl-2*H*-indazole (**1b**).	S15
**Figure S9**. ^{13}C NMR (151 MHz, $CDCl_3$) for 3-bromo-2-phenyl-2*H*-indazole (**1b**).	S16
**Figure S10**. ^{1}H NMR (600 MHz, $CDCl_3$) for 3-bromo-2-(3,4,5-trimethoxyphenyl)-2*H*-indazole (**2b**).	S17
**Figure S11**. ^{13}C NMR (151 MHz, $CDCl_3$) for 3-bromo-2-(3,4,5-trimethoxyphenyl)-2*H*-indazole (**2b**).	S18
**Figure S12**. ^{1}H NMR (600 MHz, $CDCl_3$) for 5-(3-bromo-2*H*-indazol-2-yl)-2-methoxyphenol (**3b**).	S19
**Figure S13**. ^{13}C NMR (151 MHz, $CDCl_3$) for 5-(3-bromo-2*H*-indazol-2-yl)-2-methoxyphenol (**3b**).	S20
**Figure S14**. ^{1}H NMR (600 MHz, $CDCl_3$) for 2,3-diphenyl-2*H*-indazole (**4**).	S21
**Figure S15**. ^{13}C NMR (151 MHz, $CDCl_3$) for 2,3-diphenyl-2*H*-indazole (**4**).	S22
**Figure S16**. ^{1}H NMR (600 MHz, $CDCl_3$) for 2-methoxy-5-(2-(3,4,5-trimethoxyphenyl)-2*H*-indazol-3-yl)phenol (**5**).	S23
**Figure S17**. ^{13}C NMR (151 MHz, $CDCl_3$) for 2-methoxy-5-(2-(3,4,5-trimethoxyphenyl)-2*H*-indazol-3-yl)phenol (**5**).	S24
**Figure S18**. MS (HR-ESI) for 2-methoxy-5-(2-(3,4,5-trimethoxyphenyl)-2*H*-indazol-3-yl)phenol (**5**).	S25
**Figure S19**. ^{1}H NMR (600 MHz, $CDCl_3$) for 2-methoxy-5-(3-(3,4,5-trimethoxyphenyl)-2*H*-indazol-2-yl)phenol (**6**).	S26
**Figure S20**. ^{13}C NMR (151 MHz, $CDCl_3$) for 2-methoxy-5-(3-(3,4,5-trimethoxyphenyl)-2*H*-indazol-2-yl)phenol (**6**).	S27
**Figure S21**. MS (EI) for 2-methoxy-5-(3-(3,4,5-trimethoxyphenyl)-2*H*-indazol-2-yl)phenol (**6**).	S28

Figure S22. ^{1}H NMR (600 MHz, $CDCl_3$) for 3-phenyl-1*H*-indazole (**7**). S29

Figure S23. ^{13}C NMR (151 MHz, $CDCl_3$) for 3-phenyl-1*H*-indazole (**7**). S30

Figure S24. ^{1}H NMR (600 MHz, $CDCl_3$) for 3-(3,4,5-trimethoxyphenyl)-1*H*-indazole (**8**). S31

Figure S25. ^{13}C NMR (151 MHz, $CDCl_3$) for 3-(3,4,5-trimethoxyphenyl)-1*H*-indazole (**8**). S32

Figure S26. MS (EI) for 3-(3,4,5-trimethoxyphenyl)-1*H*-indazole (**8**). S33

Figure S27. ^{1}H NMR (600 MHz, DMSO-d_6) for 5-(1*H*-indazol-3-yl)-2-methoxyphenol (**9**). S34

Figure S28. ^{13}C NMR (151 MHz, DMSO-d_6) for 5-(1*H*-indazol-3-yl)-2-methoxyphenol (**9**). S35

Figure S29. MS (EI) for 5-(1*H*-indazol-3-yl)-2-methoxyphenol (**9**). S36

Figure S30. Morphological changes of HeLa cells after 48 h of treatment. A) vehicle (DMSO); B) Compound **5** (10 μM); C) Compound **6** (10 μM). S37

Figure S31. Quantitative apoptosis assay of HeLa using Annexin V-FITC/Ghost-Red. Biparametric representation obtained from the flow cytometry assay of control HeLa cells (Control) or treated with compound **5** (0.16 μM) at 48 h. Q4 (viable cells), the apoptosis ratios (early plus late apoptotic cells, Q3 + Q2 quadrants) and Q1 (necrotic cells).

Table S1. Palladium-catalyzed arylation of 3-bromophenylindazoles and microwave assisted modification.

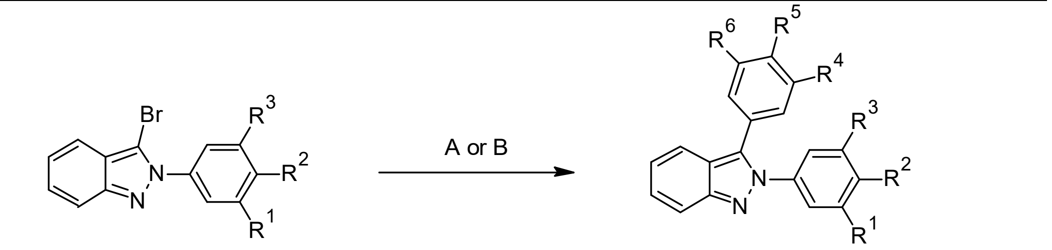

1b: $R^1 = R^2 = R^3 = H$
2b: $R^1 = R^2 = R^3 = OCH_3$
3b: $R^1 = OH$, $R^2 = OCH_3$, $R^3 = H$

4: $R^1 = R^2 = R^3 = R^4 = R^5 = R^6 = H$
5: $R^1 = R^2 = R^3 = OCH_3$, $R^4 = H$, R^5 = OC
6: $R^1 = OH$, $R^2 = OCH_3$, $R^3 = H$, $R^4 = R^5$

Compound	% Yield (Method A)[a]	% Yield (Method B)[b]
**4**	78	84
**5**	44	68
**6**	44	72

[a] Method A: Appropriate phenylboronic acid or phenylboronic acid pinacol ester (0.63 mmol, 1.05 eq), 1-propanol (3 mL), $Pd(OAc)_2$ (0.3%), $(C_6H_5)_3P$ (0.9%), Na_2CO_3 (0.72 mmol, 1.2 eq), H_2O (0.6 mL), reflux 1–3 h.

[b] Method B: Appropriate phenylboronic acid or phenylboronic acid pinacol ester (0.63 mmol, 1.05 eq), 1-propanol (3 mL), $Pd(OAc)_2$ (1%), $(C_6H_5)_3P$ (3%), Na_2CO_3 (0.72 mmol, 1.2 eq), H_2O (0.6 mL), Microwaves, 150 °C, 20 min.

Table S2. Cellular viability ± SE on HeLa cell line upon treatment with compounds 1–9 at 50 μM.

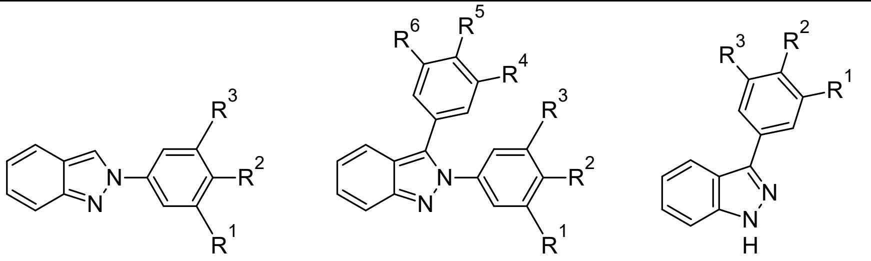

1–**3** **4**–**6** **7**–**9**

Compound	R¹	R²	R³	R⁴	R⁵	R⁶	HeLa
**1**	H	H	H	-	-	-	130.91 ± 10.17
**2**	OCH_3	OCH_3	OCH_3	-	-	-	79.38 ± 11.35
**3**	OH	OCH_3	H	-	-	-	89.36 ± 19.38
4	H	H	H	H	H	H	89.88 ± 2.38
5	OCH_3	OCH_3	OCH_3	H	OCH_3	OH	37.12 ± 4.56
6	OH	OCH_3	H	OCH_3	OCH_3	OCH_3	41.41 ± 1.09
7	H	H	H	-	-	-	111.25 ± 2.25
8	OCH_3	OCH_3	OCH_3	-	-	-	73.95 ± 2.14
9	OH	OCH_3	H	-	-	-	70.11 ± 4.70

Table S3. Antioxidant activity (IC_{50} [μM]) of compounds **4–6** employing DPPH and ABTS assays.

**Compound**	**DPPH**	**ABTS**
4	Inactive	Inactive
5	Inactive	215.7
6	Inactive	262.09
**Trolox**	21.57	71.91

Table S4. Purity of compounds **4**, **5**, and **6** by qNMR used an internal standard (% purity, mean ± standard deviation).

Compound	Signal	(S-CH_2-) %	(-CH_2-) %	(-CH_2-Si) %	% purity ± SD[a]
**4**	H7 + H4	101.03	97.38	99.32	98.01 ± 2.10
	H5	98.52	94.96	96.86	
**5**	H7	99.42	96.21	99.31	98.46 ± 1.75
	H4	98.79	95.59	98.68	
	H6	100.51	97.26	100.4	
**6**	H7	97.00	95.05	96.17	96.65 ± 1.43
	H4	96.63	94.69	95.81	
	H6	99.11	97.11	98.26	
**Std**	ArH (4H)	98.65	99.04	98.94	98.88 ± 0.20

[a] The purity of compounds was determined by qNMR using 2,2-dimethyl-2-silapentane-5-sulfonate sodium salt (DSS, 97%, Sigma-Aldrich) as an internal standard. The 4-bromobenzonitrile (99%, Sigma-Aldrich) was used for the validation of the method (**Std**). As solvent deuterated dimethyl sulfoxide-d6 (DMSO-*d6*, > 99.96%, CIL) was used. Samples were prepared as a mixture of compounds tested and DSS was dissolved in DMSO-*d6*. The measurements were carried out with an Agilent DD2 spectrometer operating at 600 MHz. In general, the experiments were acquired employing the parameters: 90 ° pulse, an acquisition time of 7 s, relaxation delay of 60 s, digital resolution of 0.8 Hz, a spectral window of 15 ppm, and a total of 64 scans. Phase and baseline corrections were done automatically using the software MestReNova v.6.0.2. The signal integration was done in automatic mode. For determination of purity were used separated signals of indazole ring in the aromatic region and three signals of DSS (CH_2) in the aliphatic region. The percentage of purity was calculated by Equation:.

$$P_{Sample} = \frac{I_{Sample}}{I_{Std}} \times \frac{N_{Std}}{N_{Sample}} \times \frac{M_{Sample}}{M_{Std}} \times \frac{m_{Std}}{m_{Sample}} \times P_{Std}$$

where I is the integrated area, N is the number of spins, M is the molar mass, m is the gravimetric weight, and P is the purity in % *w*/*w*.

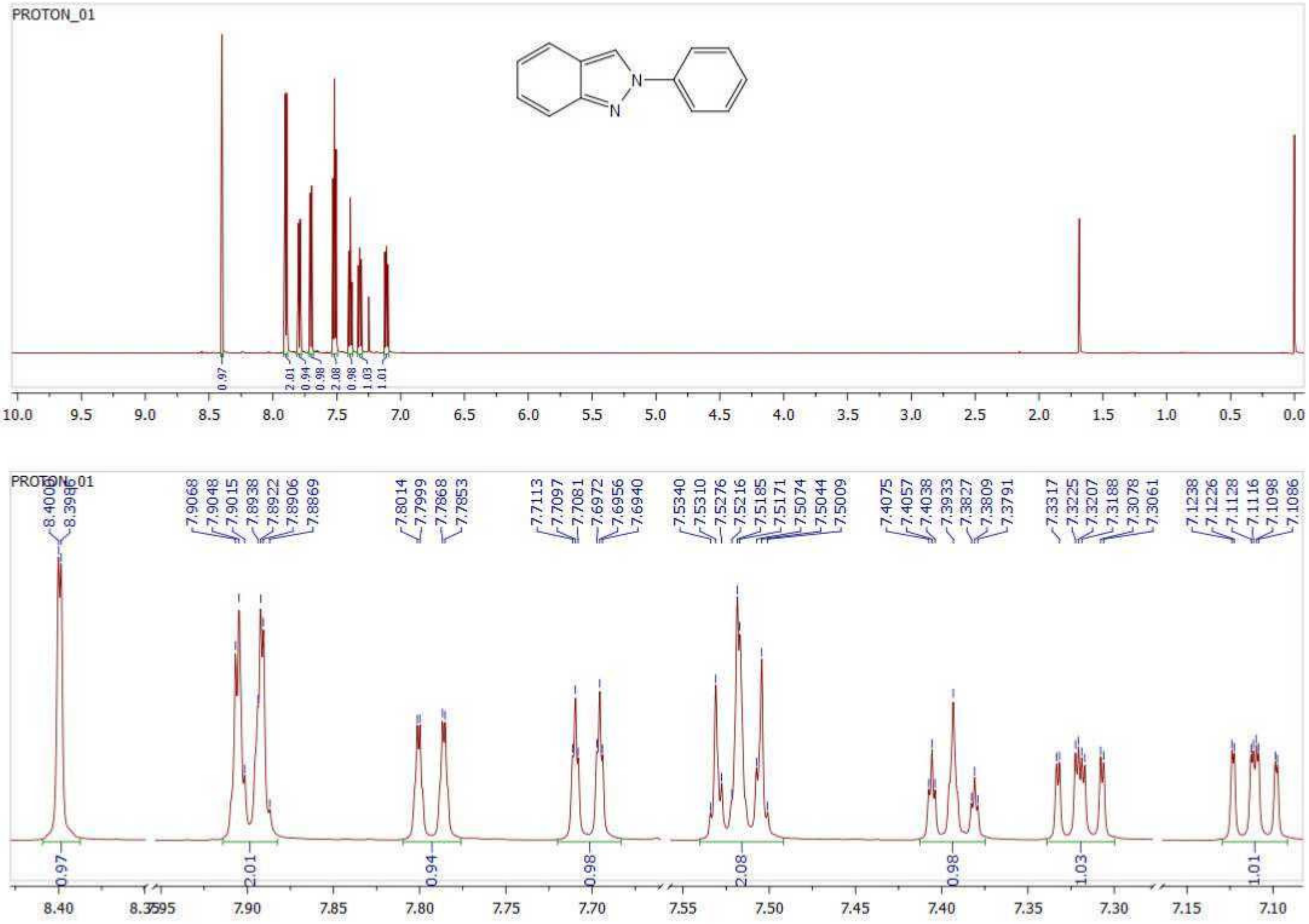

Figure S1. ^{1}H NMR (600 MHz, $CDCl_3$) for 2-phenyl-2*H*-indazole (**1**).

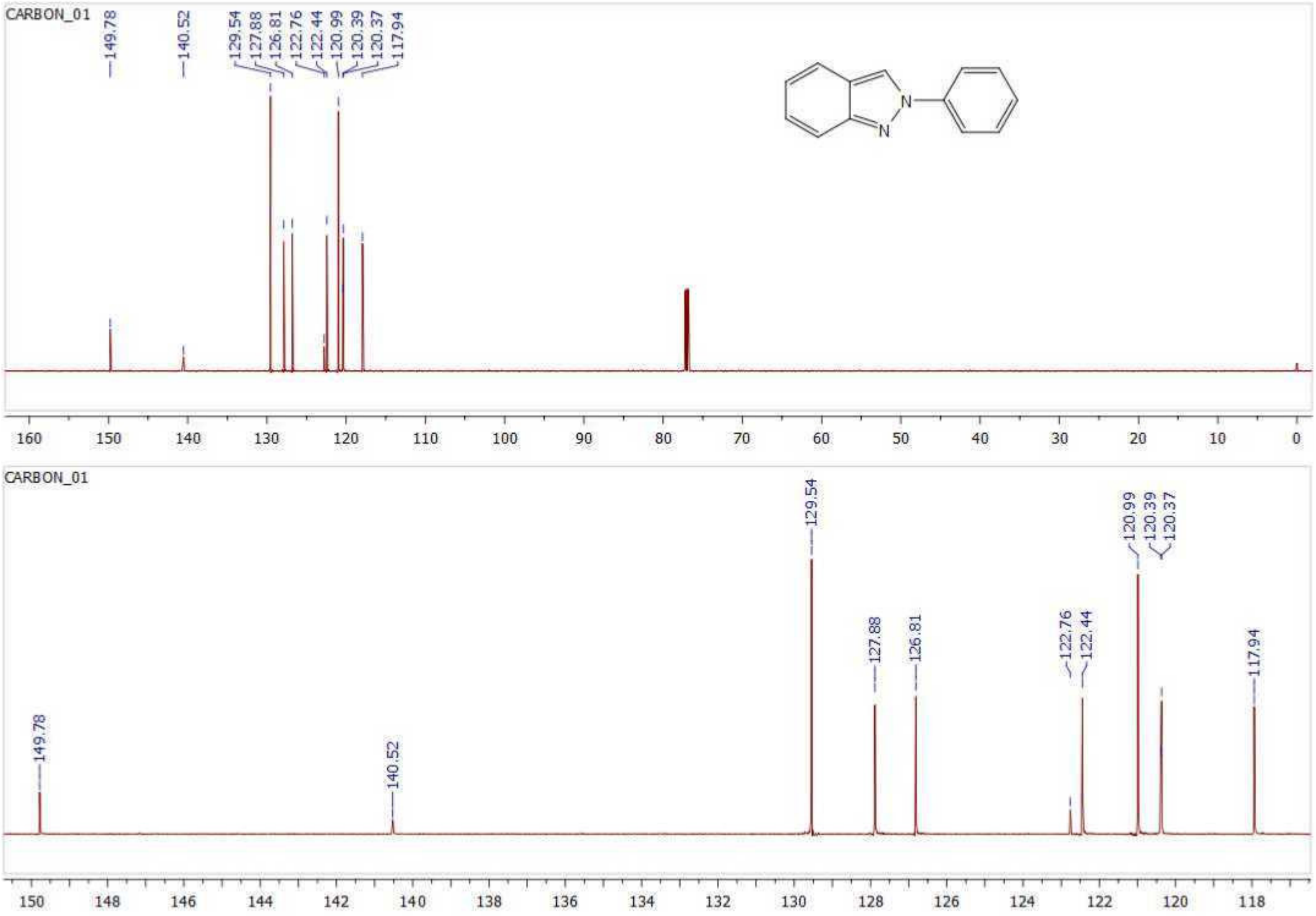

Figure S2. ^{13}C NMR (151 MHz, $CDCl_3$) for 2-phenyl-2*H*-indazole (**1**).

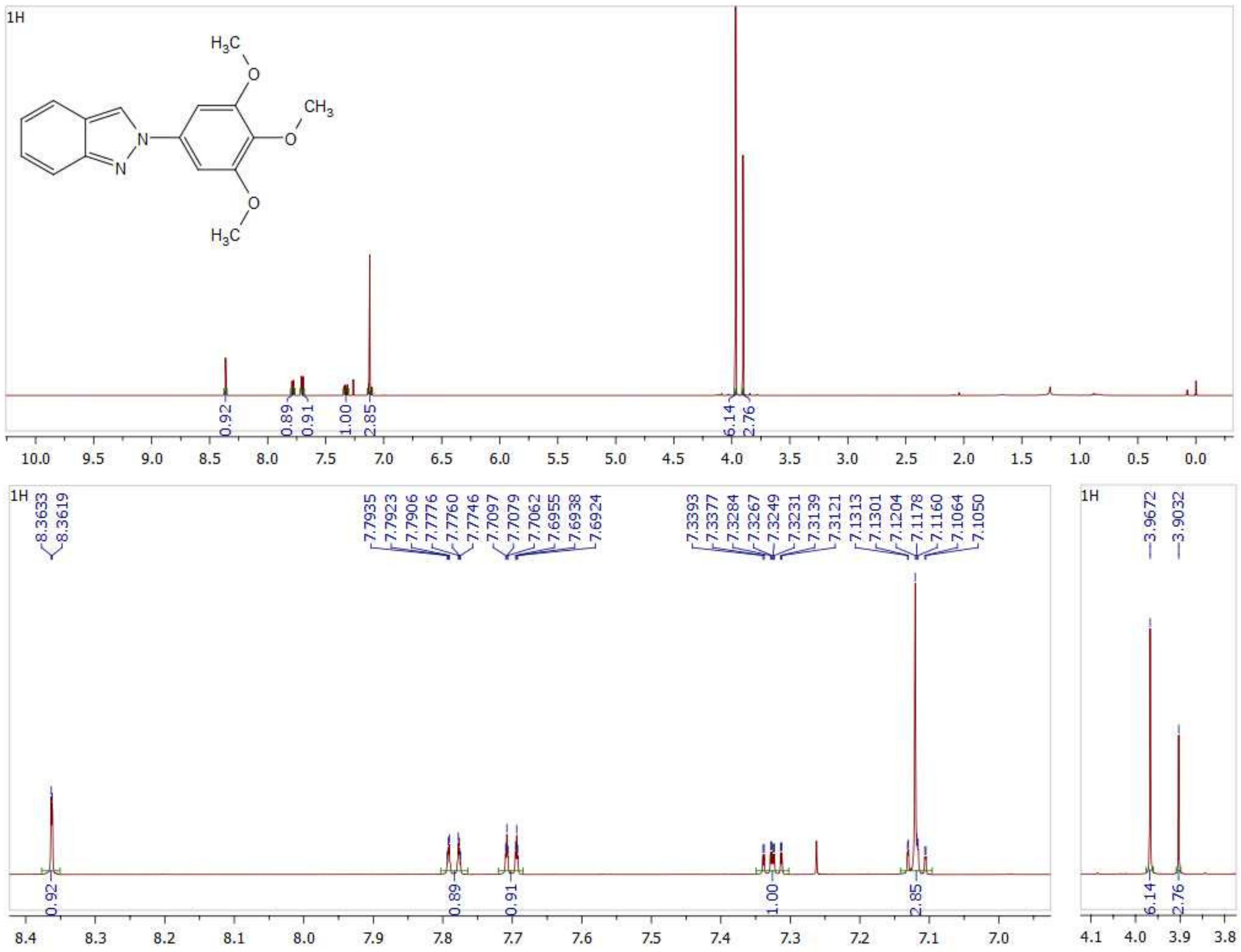

Figure S3. ^{1}H NMR (600 MHz, $CDCl_3$) for 2-(3,4,5-trimethoxyphenyl)-2*H*-indazole (**2**).

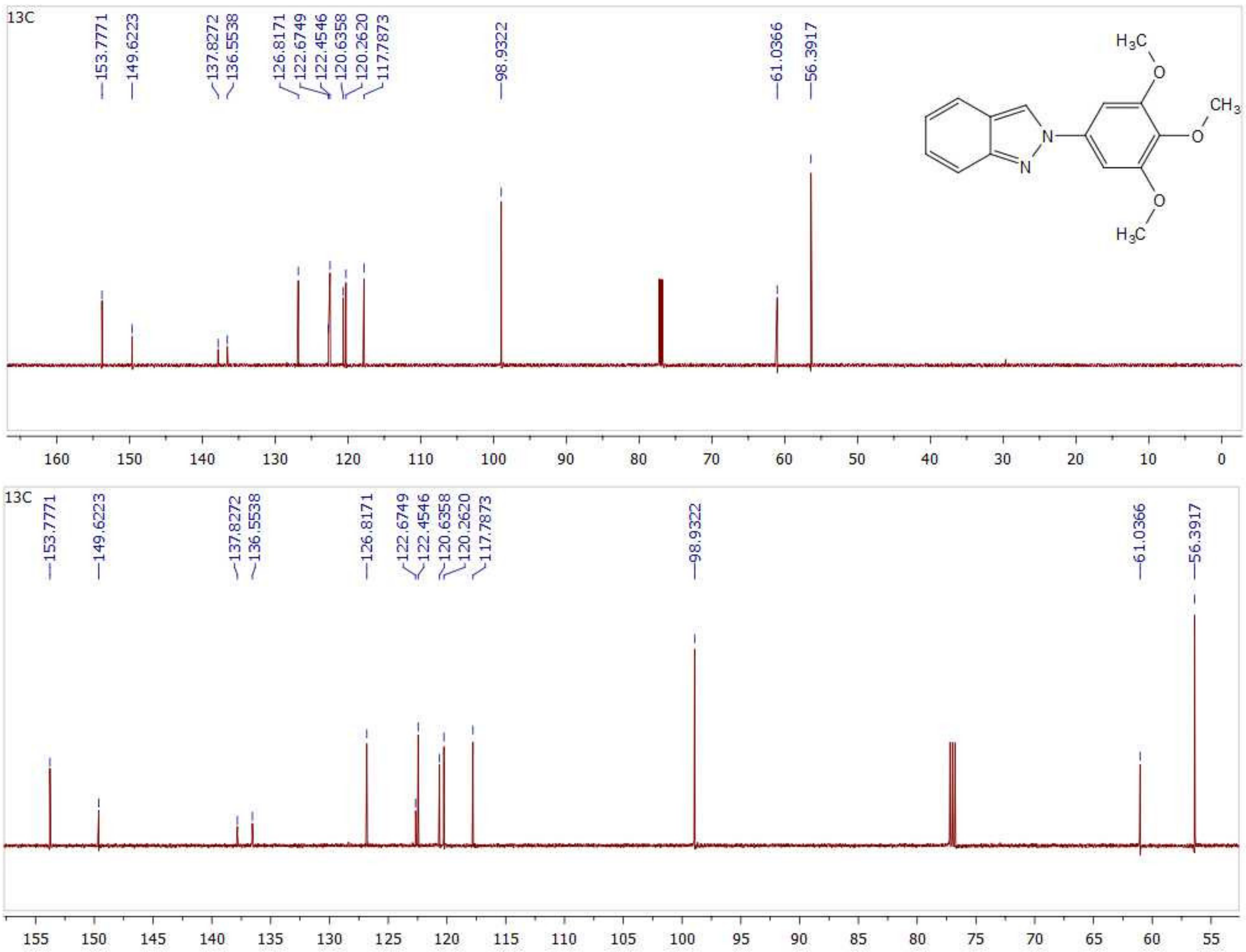

Figure S4. ^{13}C NMR (151 MHz, $CDCl_3$) for 2-(3,4,5-trimethoxyphenyl)-2*H*-indazole (**2**).

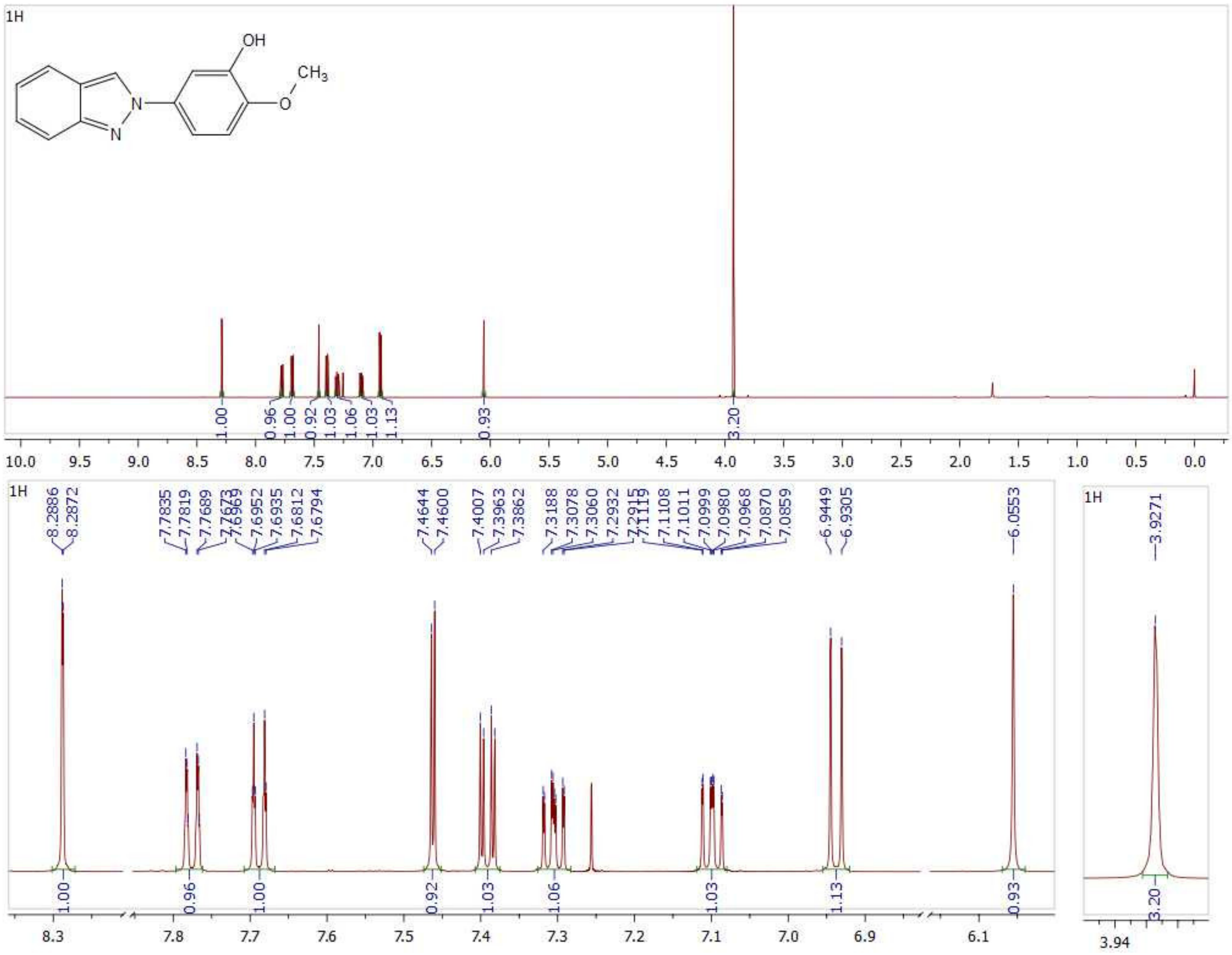

Figure S5. ^{1}H NMR (600 MHz, $CDCl_3$) for 5-(2*H*-indazol-2-yl)-2-methoxyphenol (**3**).

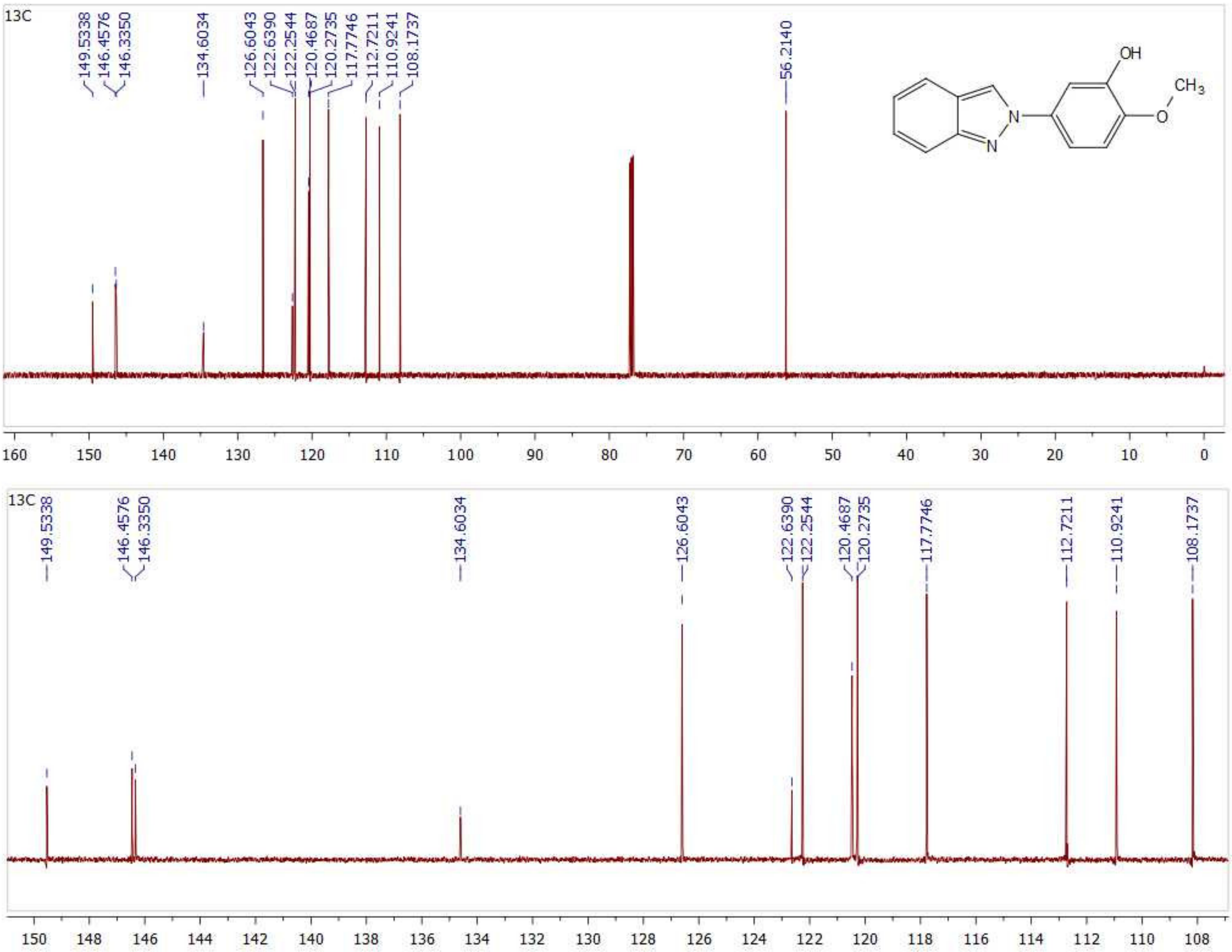

Figure S6. ^{13}C NMR (151 MHz, $CDCl_3$) for 5-(2*H*-indazol-2-yl)-2-methoxyphenol (**3**).

Acquisition Parameter

Source Type	ESI	Ion Polarity	Positive	Set Nebulizer	3.0 Bar
Focus	Active	Set Capillary	4500 V	Set Dry Heater	210 °C
Scan Begin	50 m/z	Set End Plate Offset	-500 V	Set Dry Gas	10.0 l/min
Scan End	1000 m/z	Set Charging Voltage	0 V	Set Divert Valve	Source
		Set Corona	0 nA	Set APCI Heater	0 °C

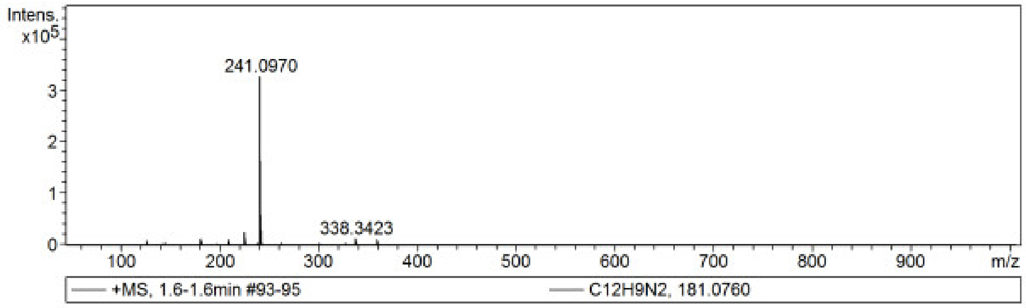

#	m/z	Res.	S/N	I	I %	FWHM
1	84.9573	4729	39.9	1124	0.3	0.0180
2	127.0243	4898	182.9	5899	1.8	0.0259
3	128.0246	7309	41.0	1332	0.4	0.0175
4	129.0420	3708	40.1	1313	0.4	0.0348
5	143.0009	6106	71.8	2472	0.8	0.0234
6	145.0333	5186	138.2	4802	1.5	0.0280
7	169.0750	4660	31.3	1222	0.4	0.0363
8	170.0802	5588	39.4	1544	0.5	0.0304
9	181.0753	5766	281.1	11552	3.5	0.0314
10	182.0752	4402	59.4	2446	0.8	0.0414
11	197.0718	4429	27.7	1190	0.4	0.0445
12	198.0791	5270	64.7	2792	0.9	0.0376
13	209.0723	5419	230.2	10114	3.1	0.0386
14	210.0768	5260	77.8	3430	1.1	0.0399
15	226.0744	5372	576.2	26461	8.1	0.0421
16	227.0780	4931	82.3	3787	1.2	0.0460
17	239.1618	5692	49.1	2338	0.7	0.0420
18	241.0970	6124	6829.7	326061	100.0	0.0394
19	241.4577	3030	27.5	1313	0.4	0.0797
20	242.1004	5641	1213.5	58035	17.8	0.0429
21	243.1044	5030	117.2	5609	1.7	0.0483
22	263.0803	5769	68.7	3366	1.0	0.0456
23	301.1407	4860	35.6	1762	0.5	0.0620
24	328.1443	6083	69.4	3462	1.1	0.0539
25	338.3423	5328	248.1	12292	3.8	0.0635
26	339.3441	5201	57.8	2859	0.9	0.0652
27	360.3227	5389	247.7	11967	3.7	0.0669
28	361.3268	5406	63.8	3080	0.9	0.0668
29	368.4263	4701	28.1	1344	0.4	0.0784
30	479.1714	5219	32.0	1235	0.4	0.0918

Figure S7. MS (HR-ESI) for 5-(2*H*-indazol-2-yl)-2-methoxyphenol (**3**).

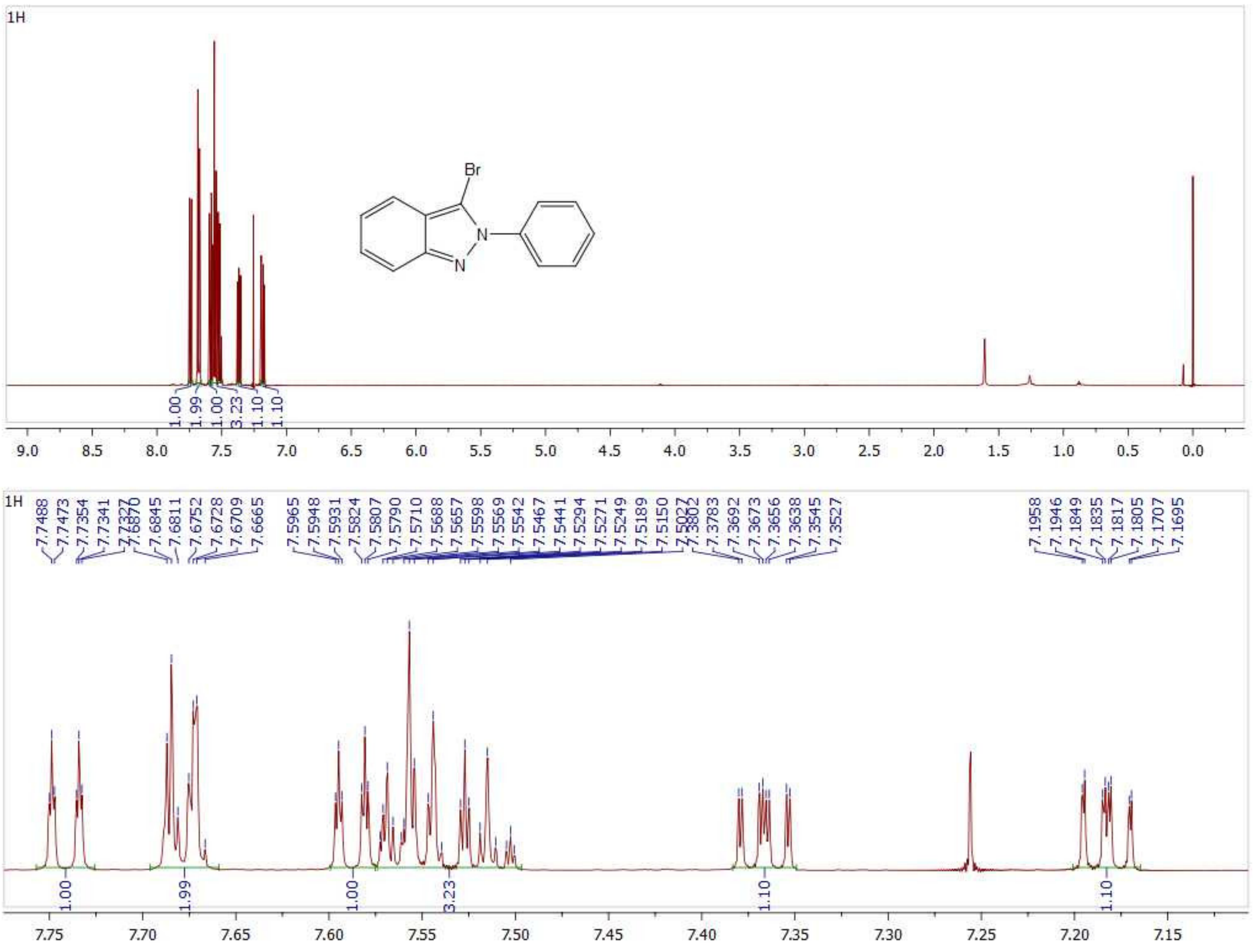

Figure S8. ^{1}H NMR (600 MHz, $CDCl_3$) for 3-bromo-2-phenyl-2*H*-indazole (**1b**).

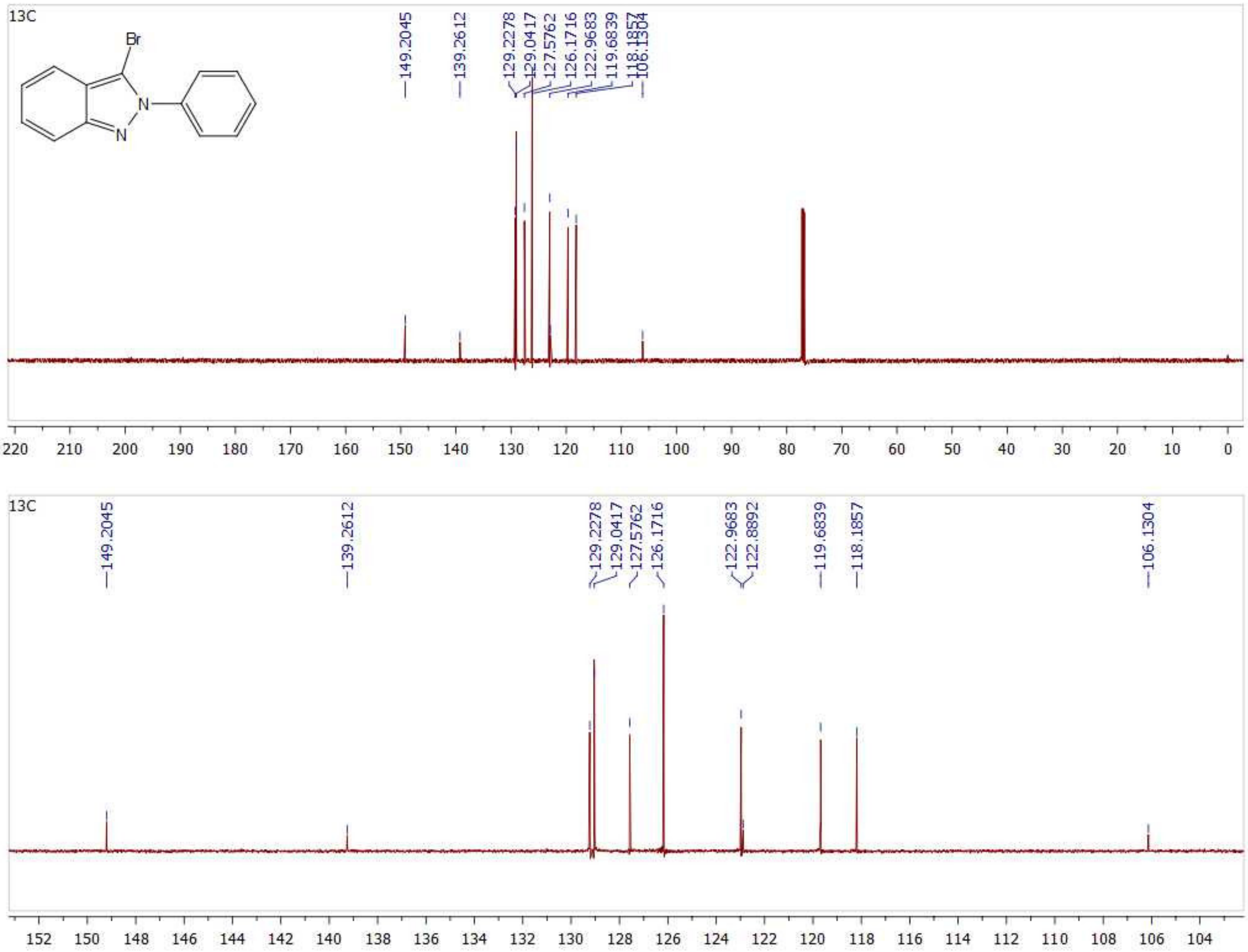

Figure S9. ^{13}C NMR (151 MHz, $CDCl_3$) for 3-bromo-2-phenyl-2*H*-indazole (**1b**).

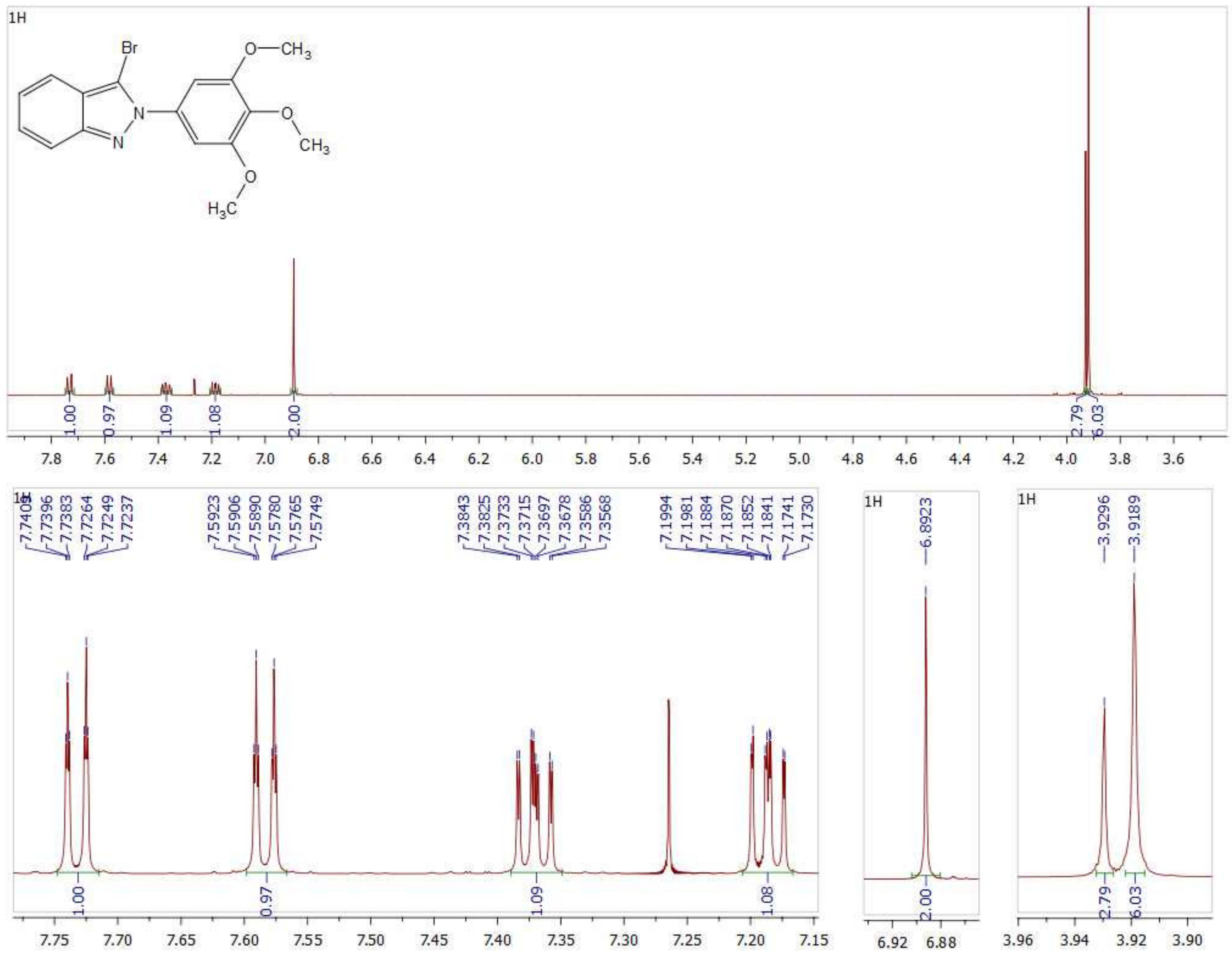

Figure S10. ^{1}H NMR (600 MHz, $CDCl_3$) for 3-bromo-2-(3,4,5-trimethoxyphenyl)-2*H*-indazole (**2b**).

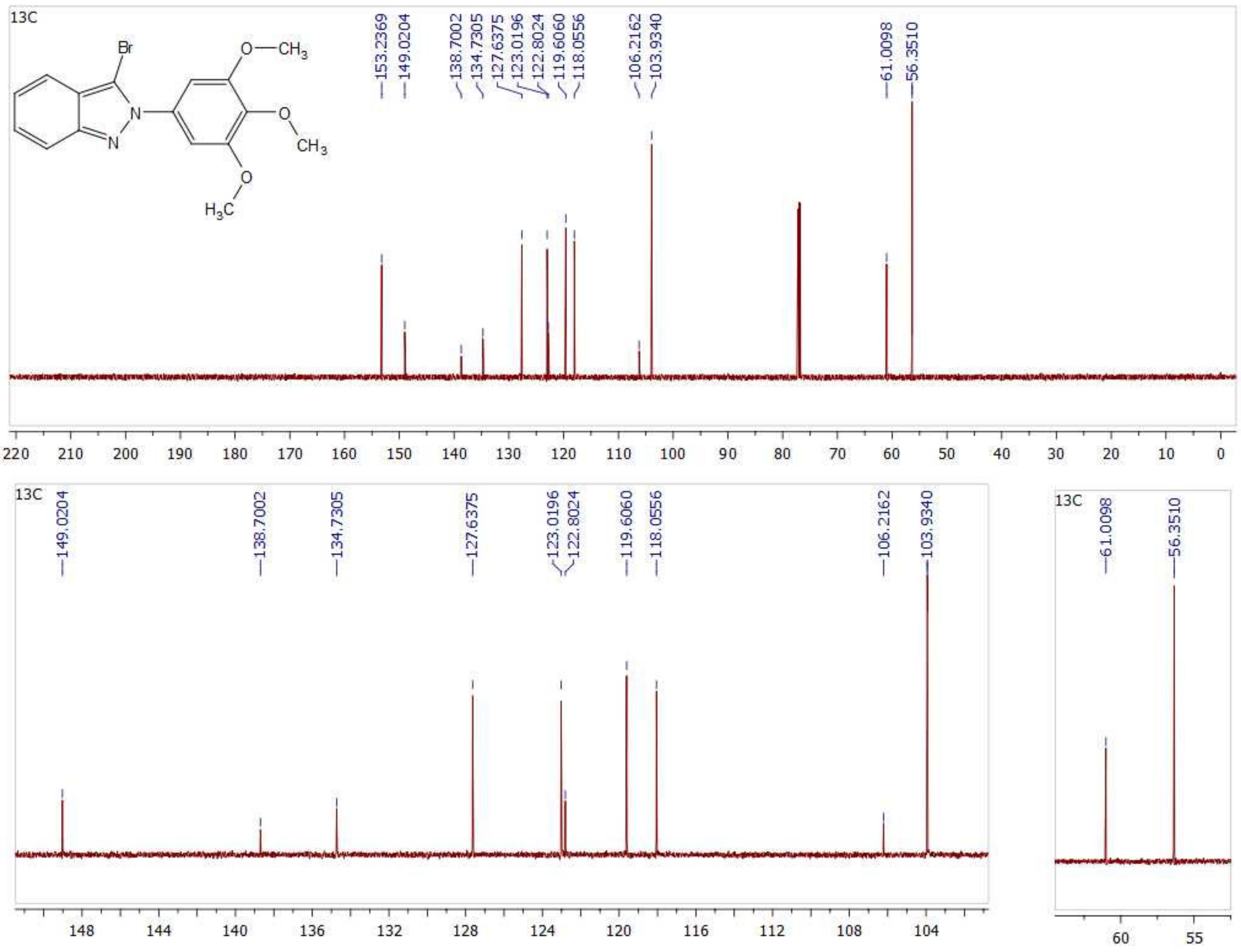

Figure S11. ^{13}C NMR (151 MHz, $CDCl_3$) for 3-bromo-2-(3,4,5-trimethoxyphenyl)-2*H*-indazole (**2b**).

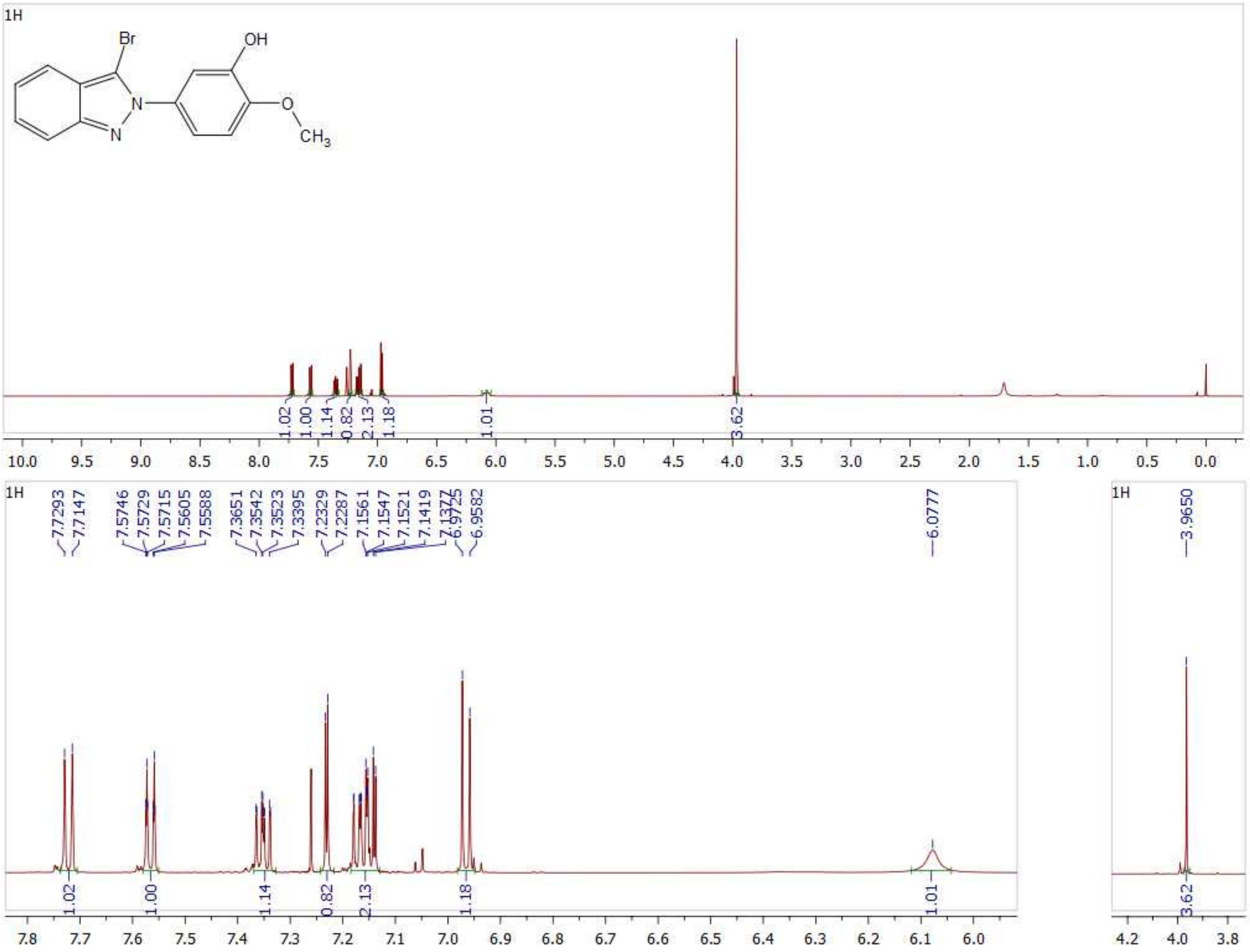

Figure S12. ^1H NMR (600 MHz, $CDCl_3$) for 5-(3-bromo-2*H*-indazol-2-yl)-2-methoxyphenol (**3b**).

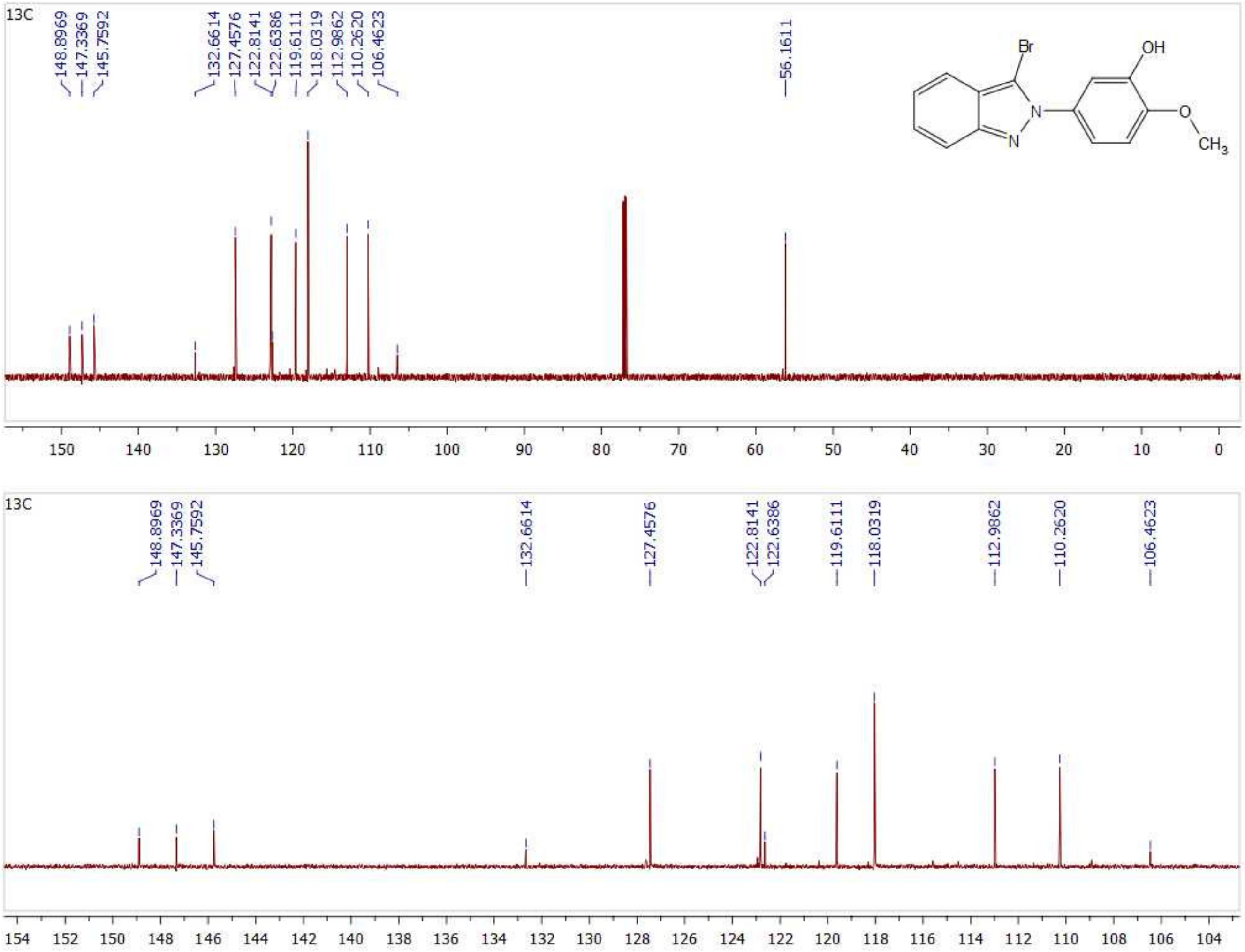

Figure S13. ^{13}C NMR (151 MHz, $CDCl_3$) for 5-(3-bromo-2*H*-indazol-2-yl)-2-methoxyphenol (**3b**).

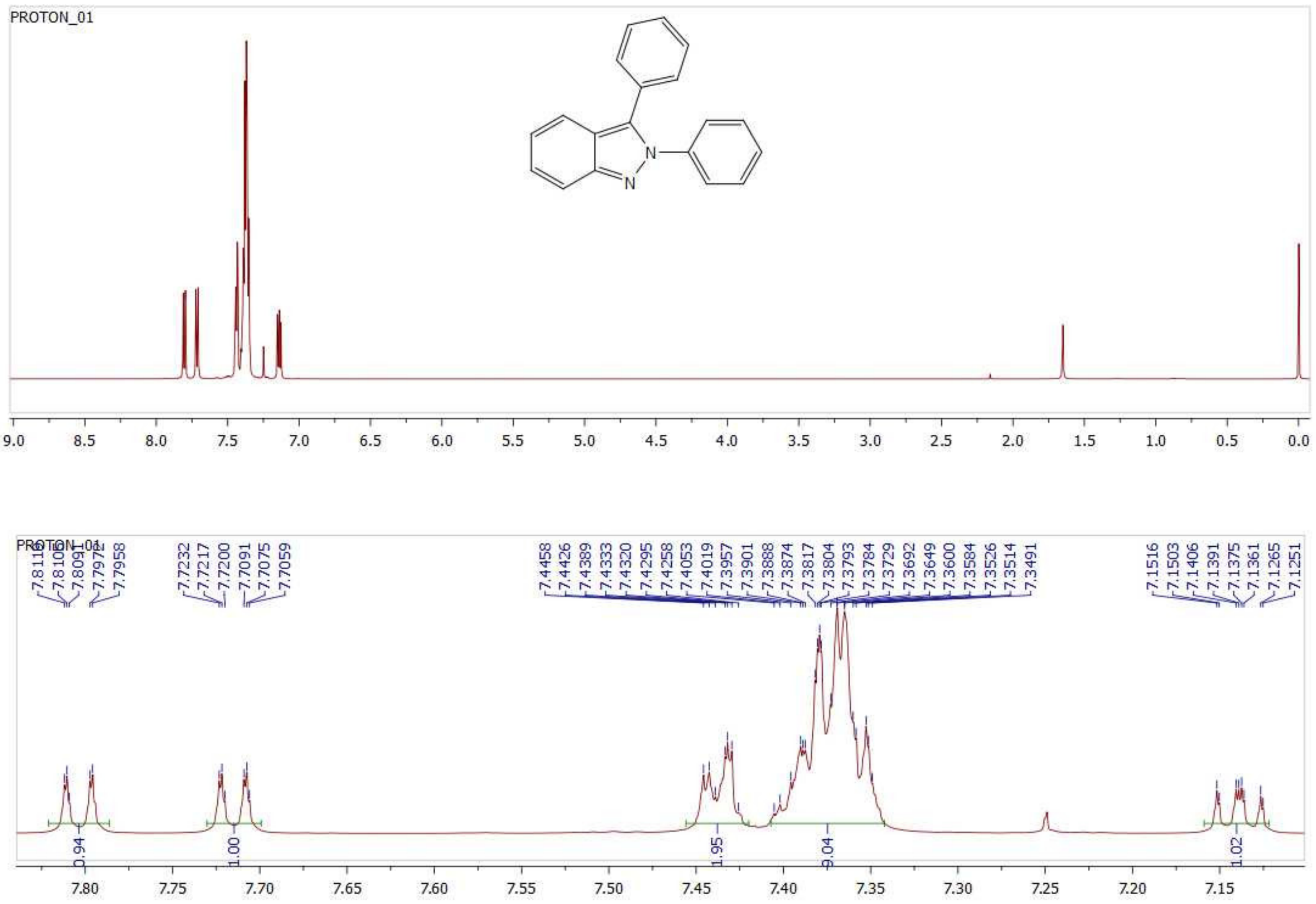

Figure S14. ^{1}H NMR (600 MHz, $CDCl_3$) for 2,3-diphenyl-2*H*-indazole (**4**).

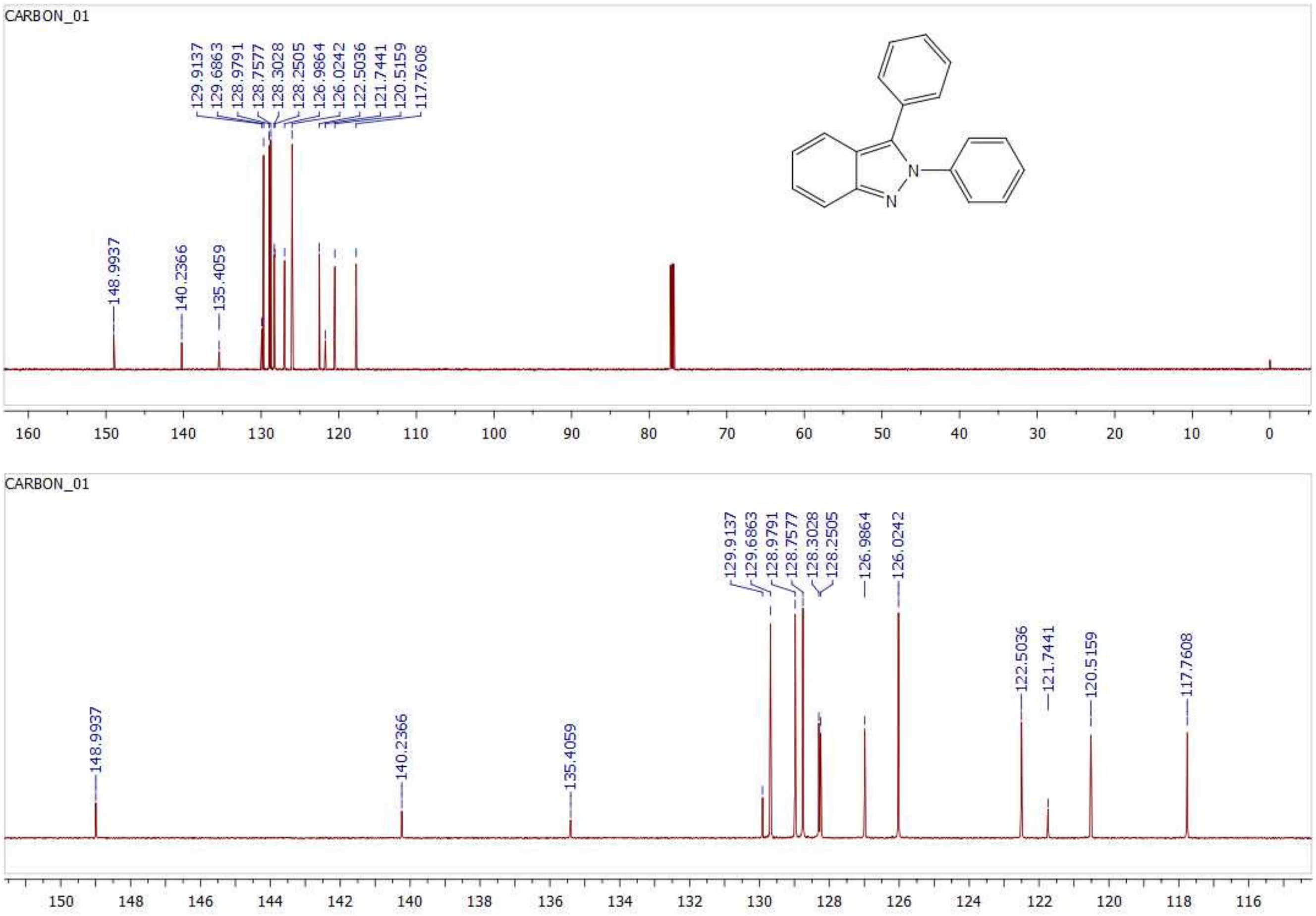

Figure S15. ^{13}C NMR (151 MHz, $CDCl_3$) for 2,3-diphenyl-2*H*-indazole (**4**).

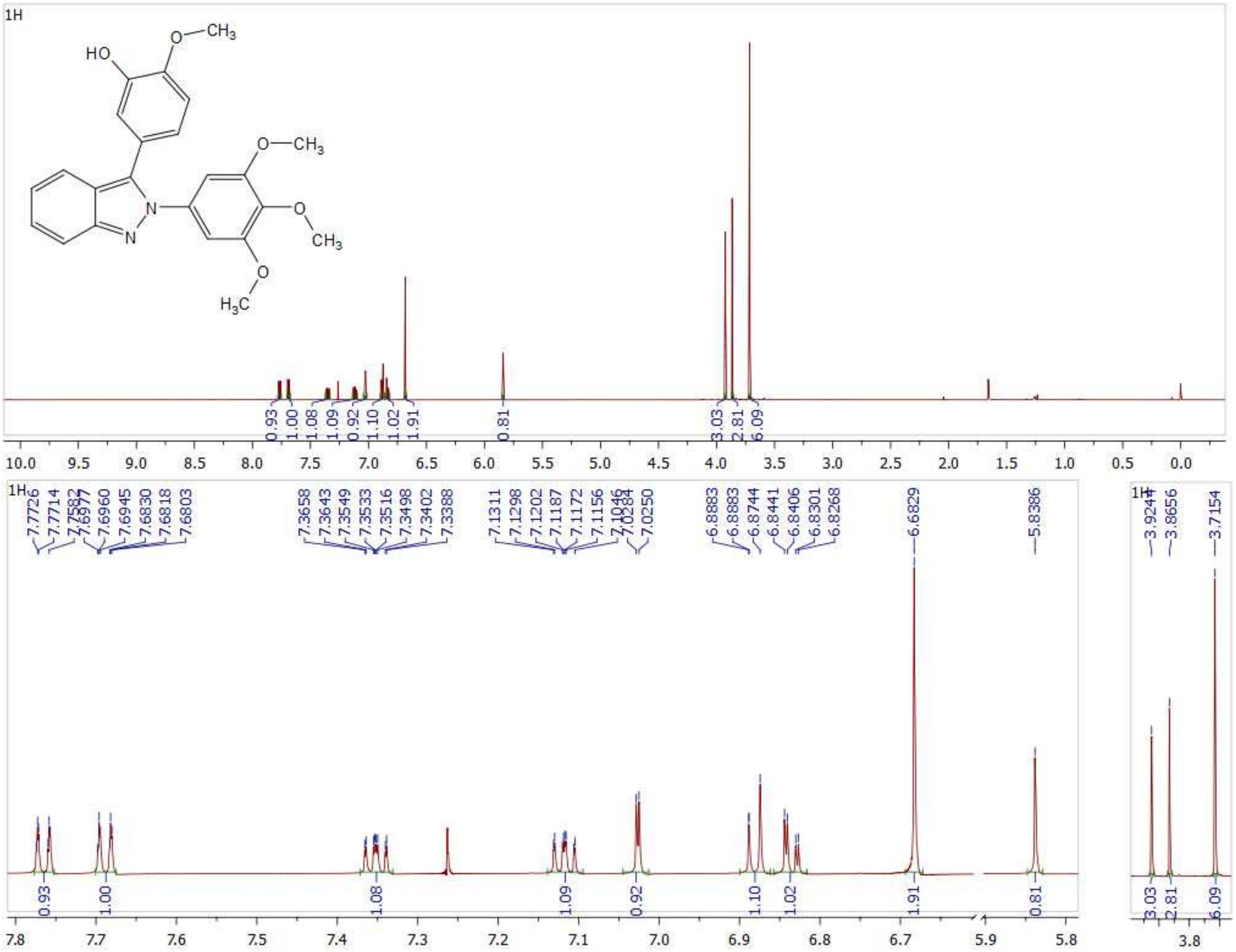

Figure S16. ^{1}H NMR (600 MHz, $CDCl_3$) for 2-methoxy-5-(2-(3,4,5-trimethoxyphenyl)-2*H*-indazol-3-yl)phenol (**5**).

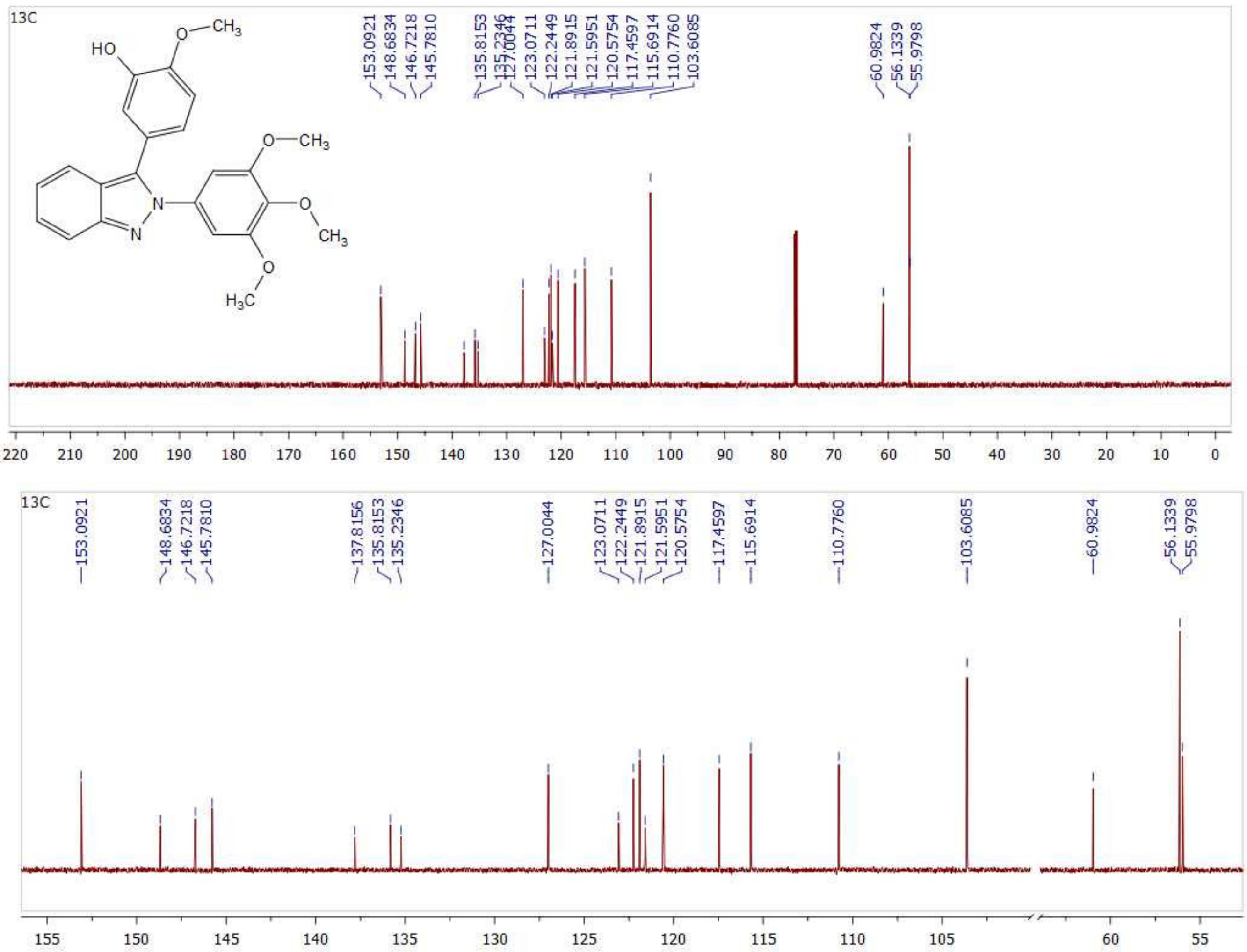

Figure S17. ^{13}C NMR (151 MHz, $CDCl_3$) for 2-methoxy-5-(2-(3,4,5-trimethoxyphenyl)-2*H*-indazol-3-yl)phenol (**5**).

Acquisition Parameter

Source Type	ESI	Ion Polarity	Positive	Set Nebulizer	3.0 Bar
Focus	Active	Set Capillary	4500 V	Set Dry Heater	210 °C
Scan Begin	50 m/z	Set End Plate Offset	-500 V	Set Dry Gas	10.0 l/min
Scan End	1000 m/z	Set Charging Voltage	0 V	Set Divert Valve	Source
		Set Corona	0 nA	Set APCI Heater	0 °C

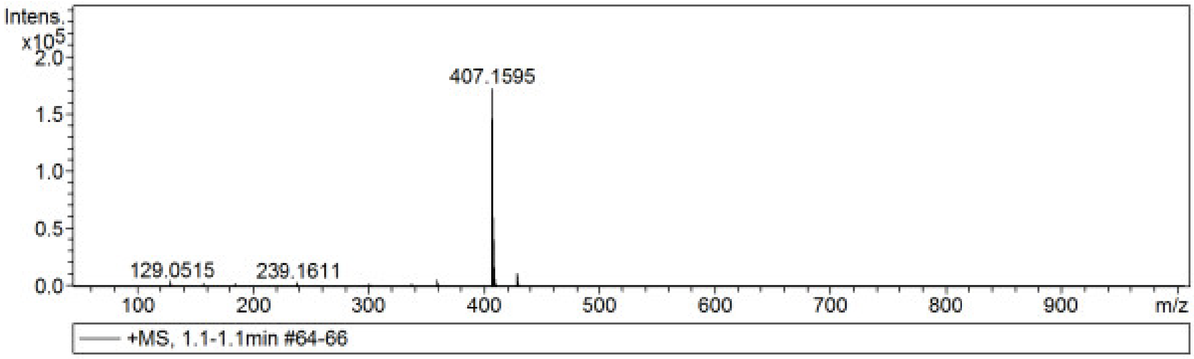

#	m/z	Res.	S/N	I	I %	FWHM
1	129.0515	5073	129.4	3099	1.8	0.0254
2	155.0532	4673	13.3	331	0.2	0.0332
3	157.0659	5359	113.6	2828	1.6	0.0293
4	185.1139	4823	102.9	2651	1.5	0.0384
5	213.1454	5863	25.1	671	0.4	0.0364
6	227.1257	4830	12.0	327	0.2	0.0470
7	239.1611	5785	95.7	2667	1.6	0.0413
8	250.1787	5570	16.8	476	0.3	0.0449
9	301.1428	5275	54.8	1704	1.0	0.0571
10	302.1458	9757	10.9	339	0.2	0.0310
11	338.3436	6810	25.5	846	0.5	0.0497
12	360.3249	5786	120.2	4039	2.4	0.0623
13	361.3269	5182	30.4	1021	0.6	0.0697
14	405.0017	4781	10.2	356	0.2	0.0847
15	405.1376	3192	9.3	323	0.2	0.1269
16	407.1595	6079	4937.0	171637	100.0	0.0670
17	407.6048	3202	25.3	880	0.5	0.1273
18	407.7180	3558	27.2	945	0.6	0.1146
19	407.8351	3716	28.7	999	0.6	0.1098
20	408.1630	5621	1353.9	47119	27.5	0.0726
21	408.7816	4406	11.8	410	0.2	0.0928
22	409.1664	5799	242.7	8451	4.9	0.0706
23	410.1670	5297	26.3	917	0.5	0.0774
24	429.1413	5326	327.5	11406	6.6	0.0806
25	430.1448	5353	81.8	2849	1.7	0.0804
26	431.1475	5984	15.9	554	0.3	0.0720
27	449.1973	6927	14.7	506	0.3	0.0649
28	453.7874	4980	9.1	310	0.2	0.0911
29	563.2341	6368	13.1	401	0.2	0.0884
30	645.3045	6808	12.2	330	0.2	0.0948

Figure S18. MS (HR-ESI) for 2-methoxy-5-(2-(3,4,5-trimethoxyphenyl)-2*H*-indazol-3-yl)phenol (**5**).

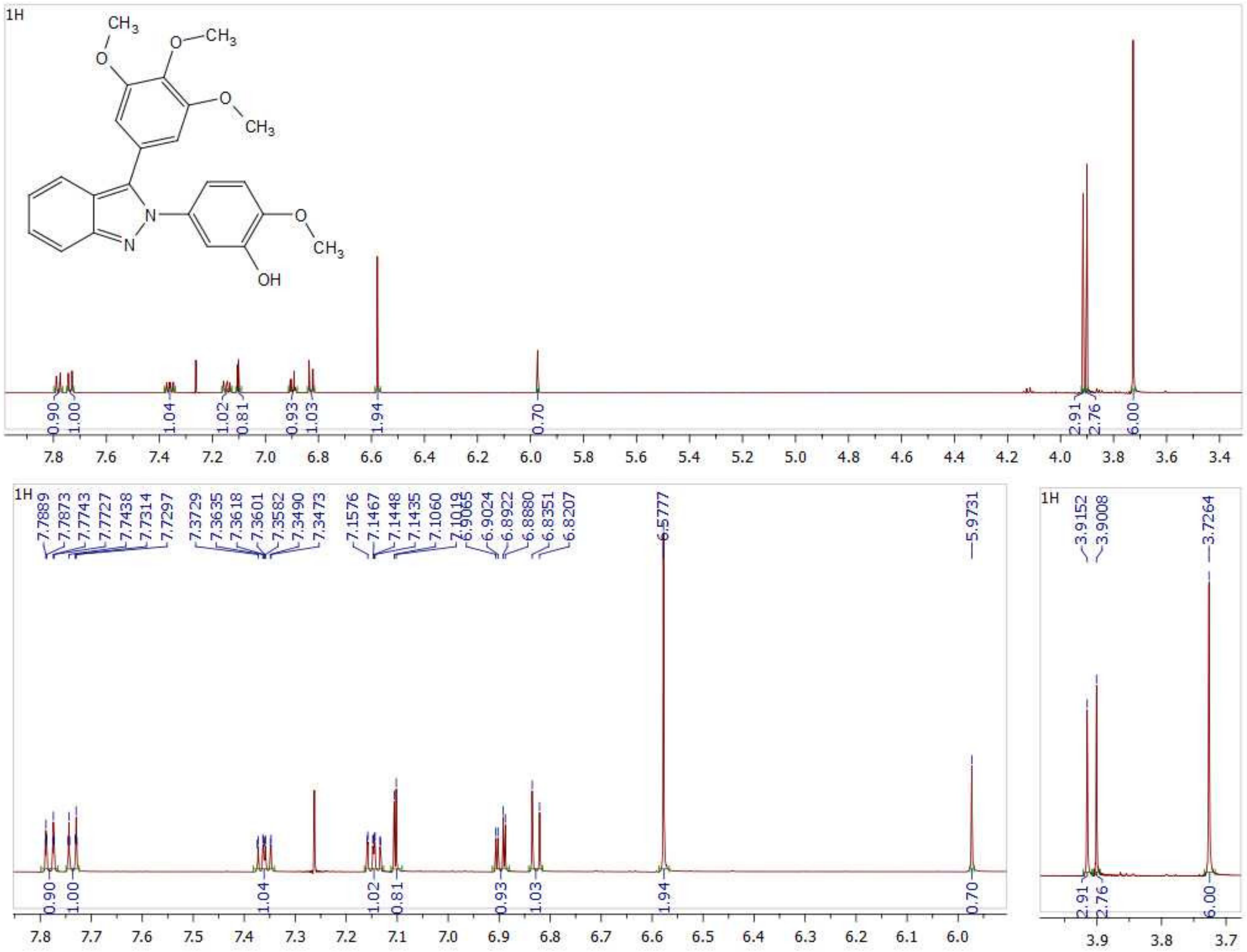

Figure S19. ^{1}H NMR (600 MHz, $CDCl_3$) for 2-methoxy-5-(3-(3,4,5-trimethoxyphenyl)-2*H*-indazol-2-yl)phenol (**6**).

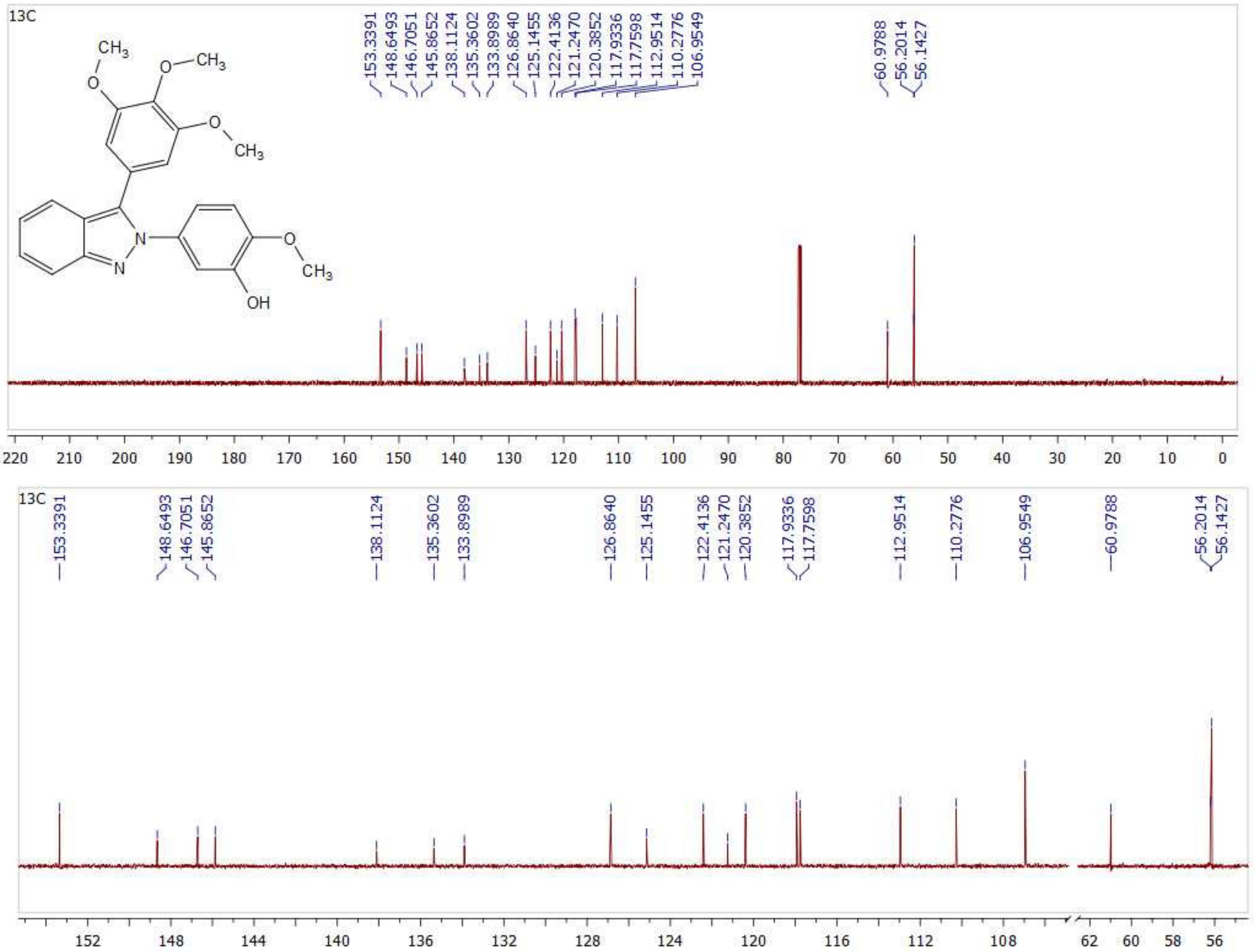

Figure S20. ^{13}C NMR (151 MHz, $CDCl_3$) for 2-methoxy-5-(3-(3,4,5-trimethoxyphenyl)-2*H*-indazol-2-yl)phenol (**6**).

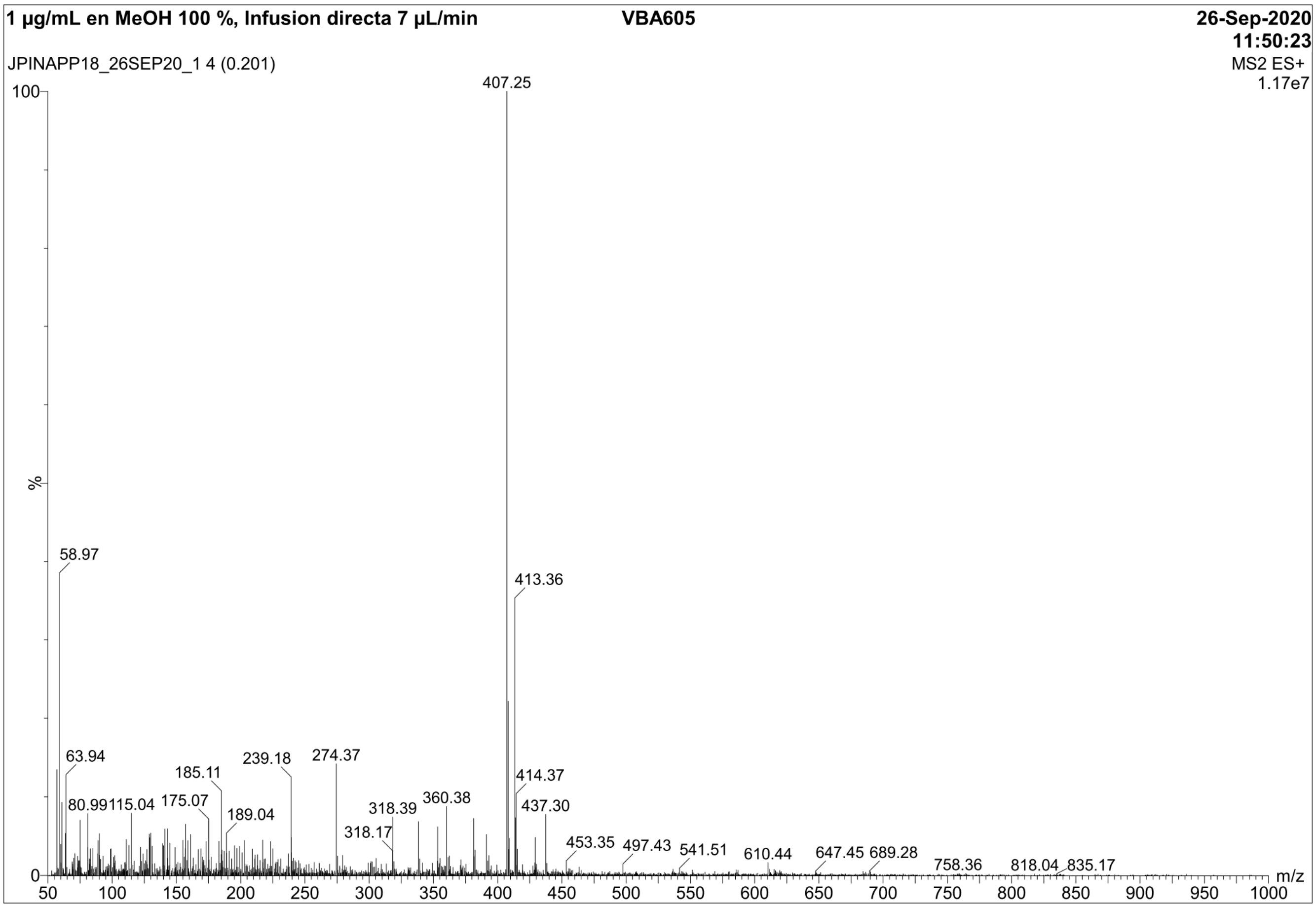

Figure S21. MS (EI) for 2-methoxy-5-(3-(3,4,5-trimethoxyphenyl)-2*H*-indazol-2-yl)phenol (**6**).

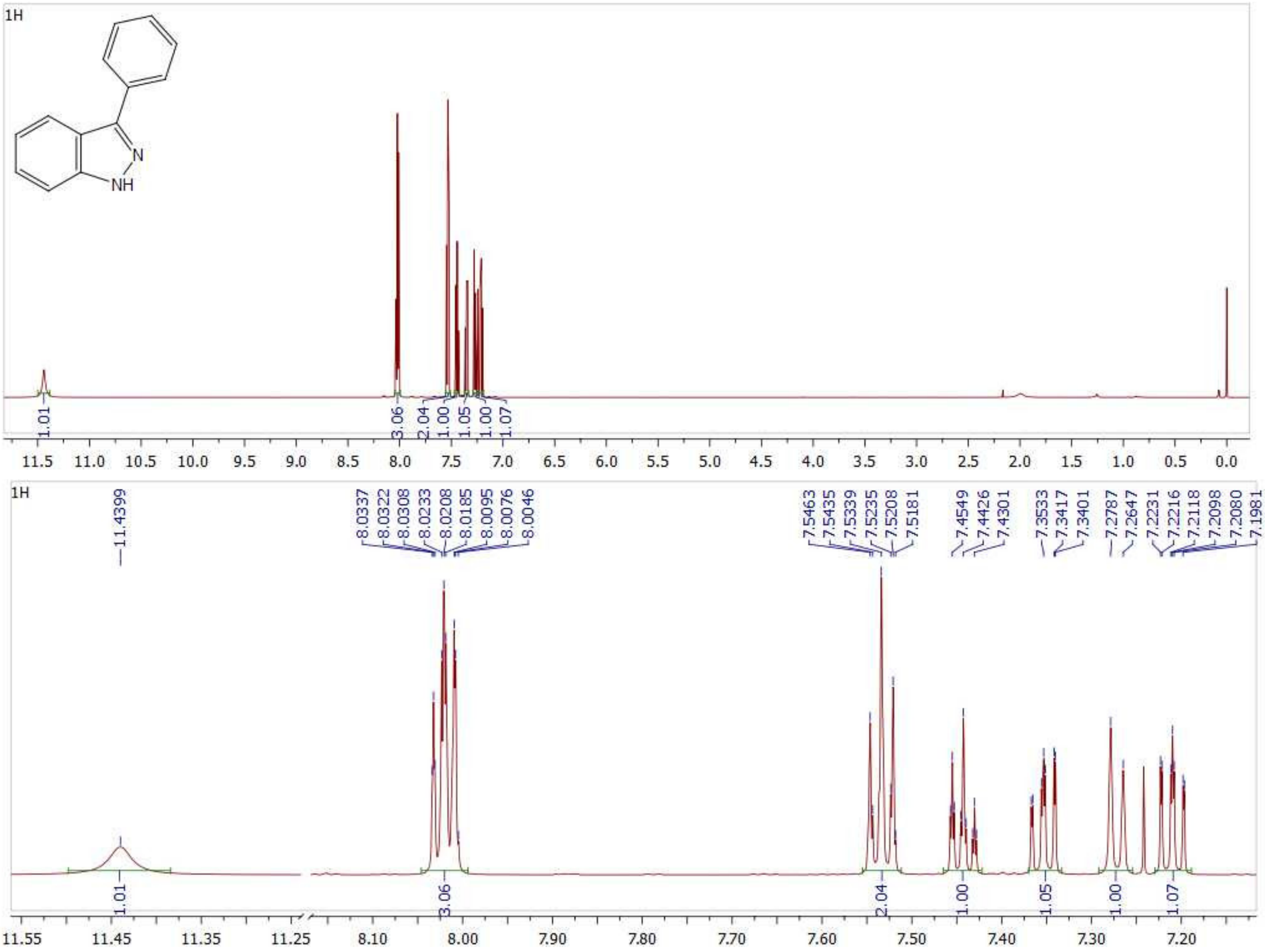

Figure S22. ^{1}H NMR (600 MHz, $CDCl_3$) for 3-phenyl-1*H*-indazole (**7**).

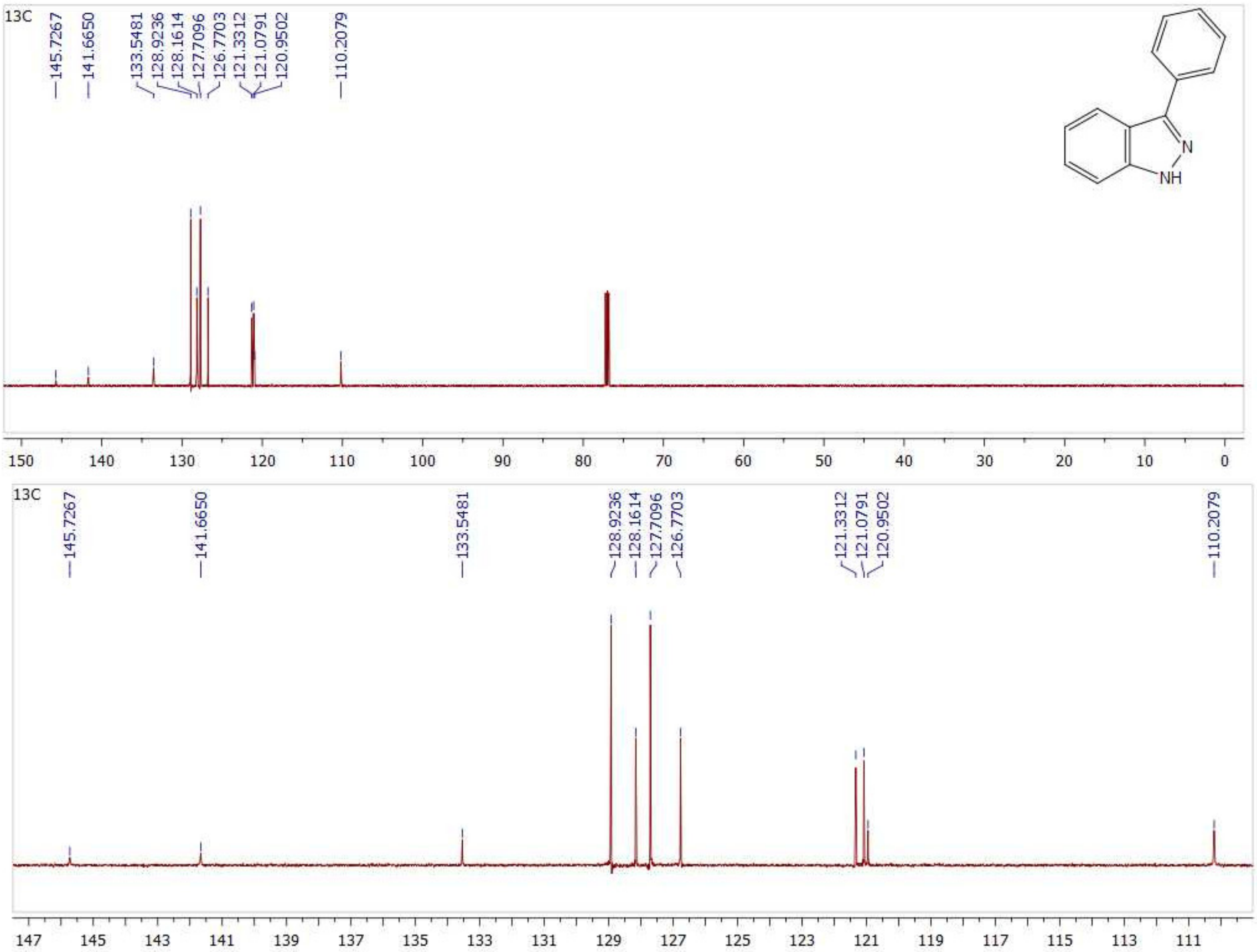

Figure S23. ^{13}C NMR (151 MHz, $CDCl_3$) for 3-phenyl-1*H*-indazole (**7**).

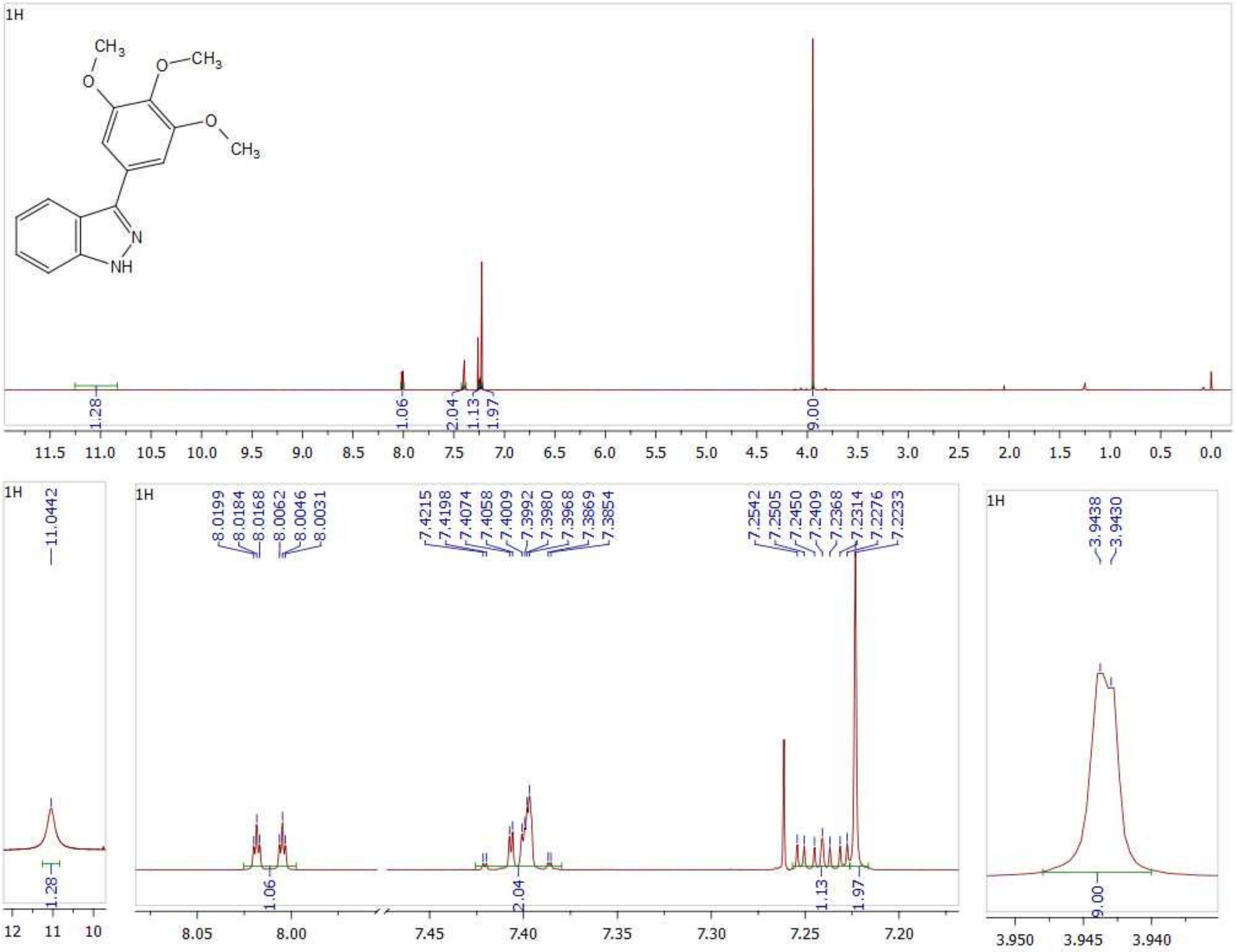

Figure S24. ^{1}H NMR (600 MHz, $CDCl_3$) for 3-(3,4,5-trimethoxyphenyl)-1*H*-indazole (**8**).

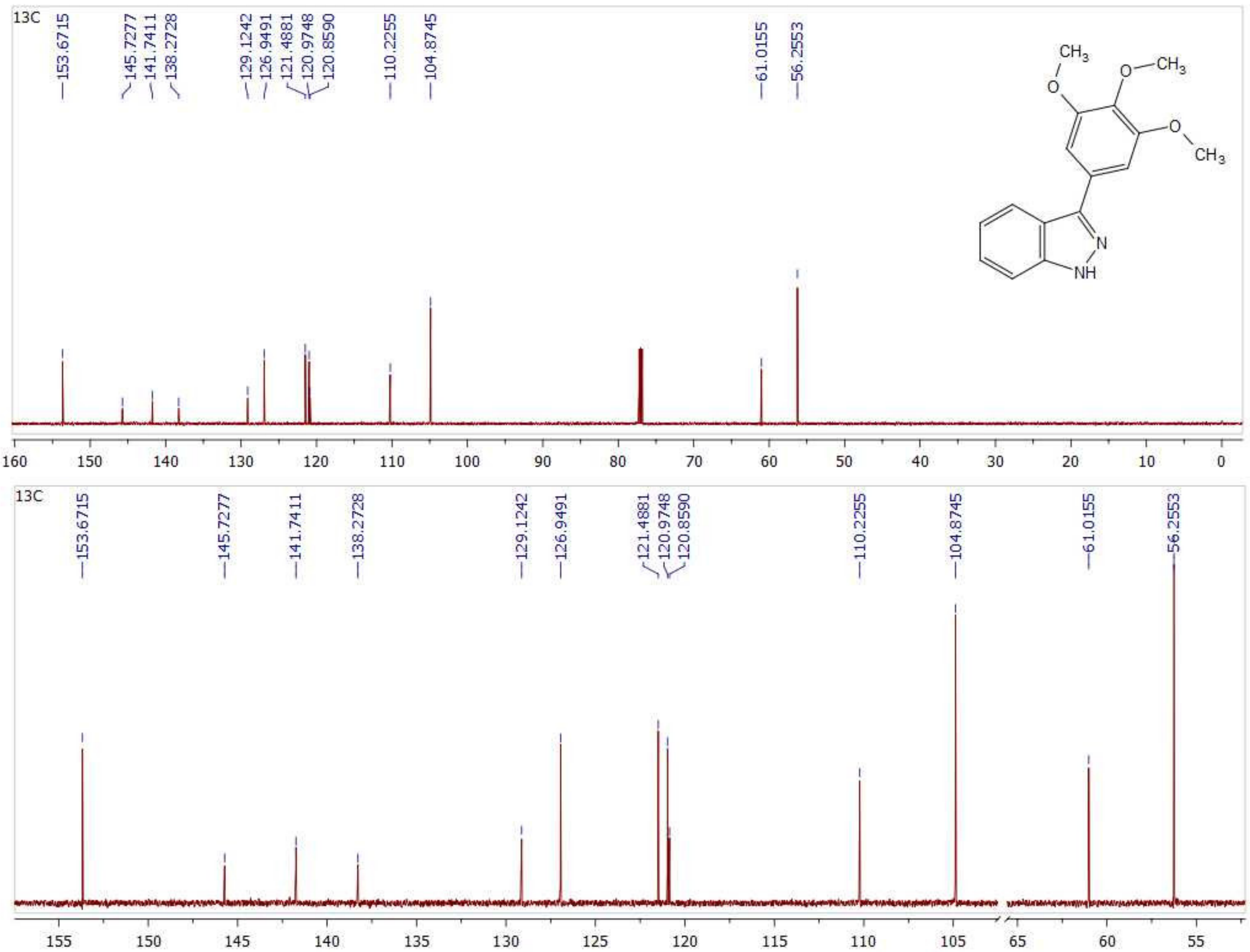

Figure S25. ^{13}C NMR (151 MHz, $CDCl_3$) for 3-(3,4,5-trimethoxyphenyl)-1*H*-indazole (**8**).

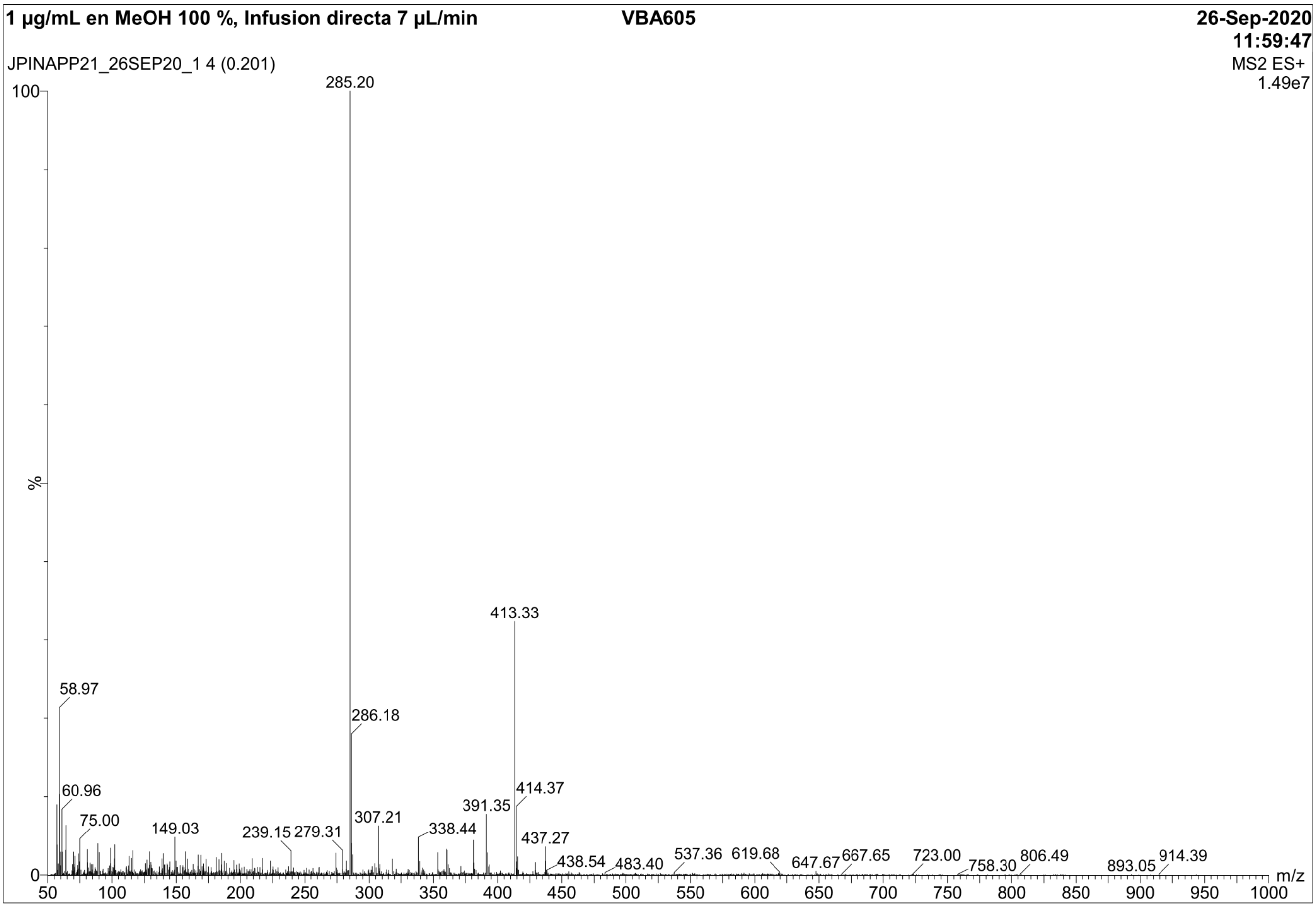

Figure S26. MS (EI) for 3-(3,4,5-trimethoxyphenyl)-1*H*-indazole (**8**).

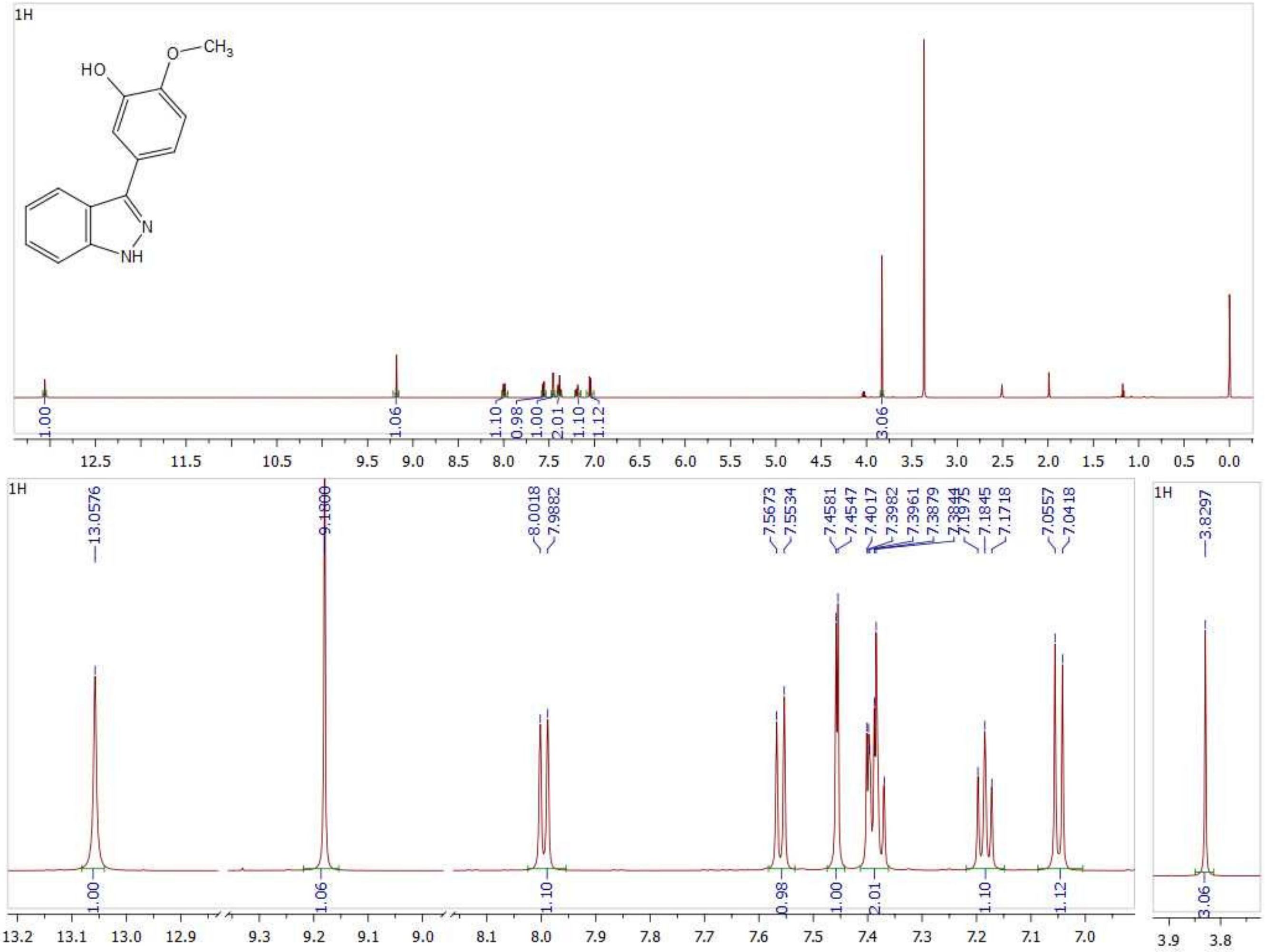

Figure S27. ^{1}H NMR (600 MHz, DMSO-d_6) for 5-(1*H*-indazol-3-yl)-2-methoxyphenol (**9**).

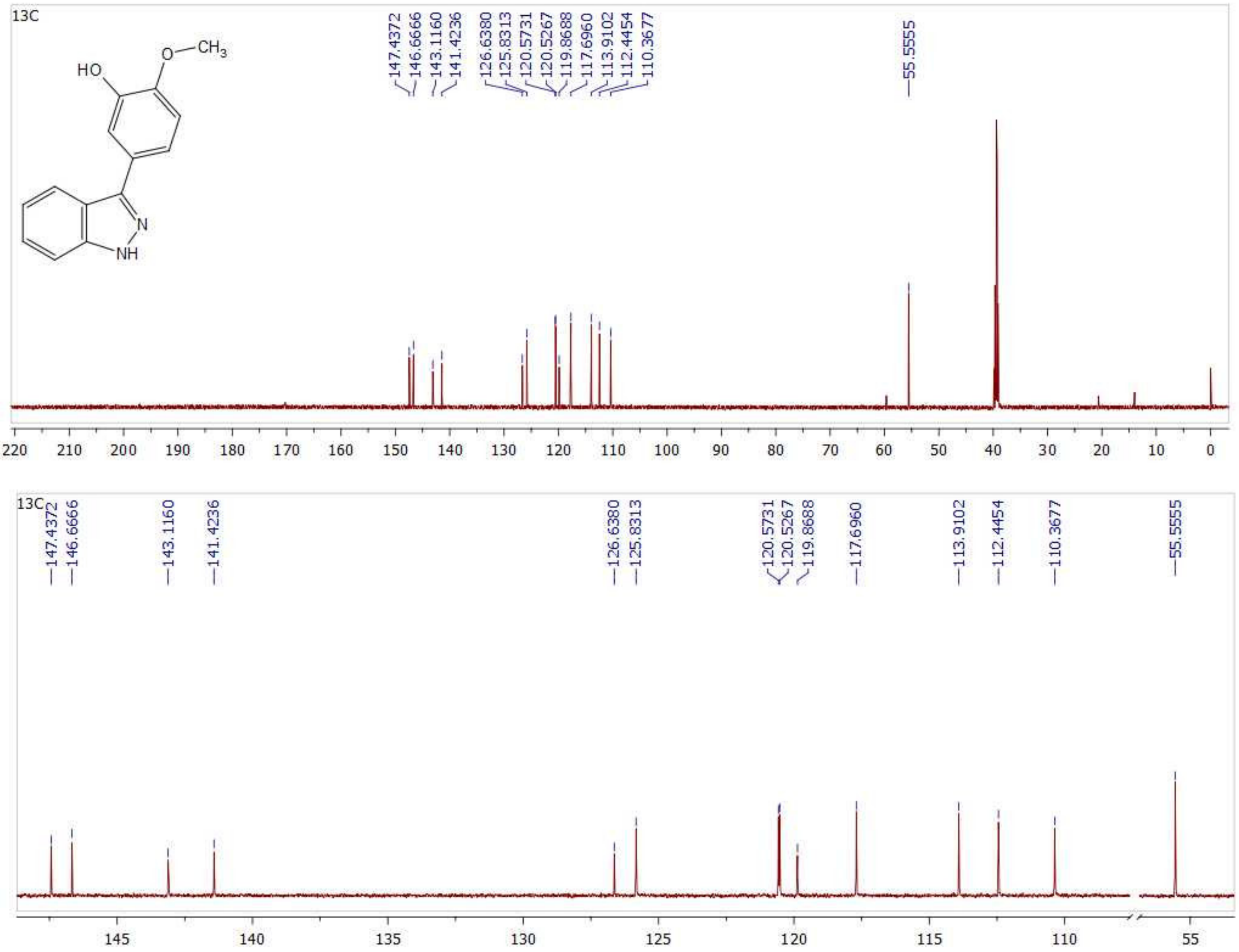

Figure S28. ^{13}C NMR (151 MHz, DMSO-d_6) for 5-(1*H*-indazol-3-yl)-2-methoxyphenol (**9**).

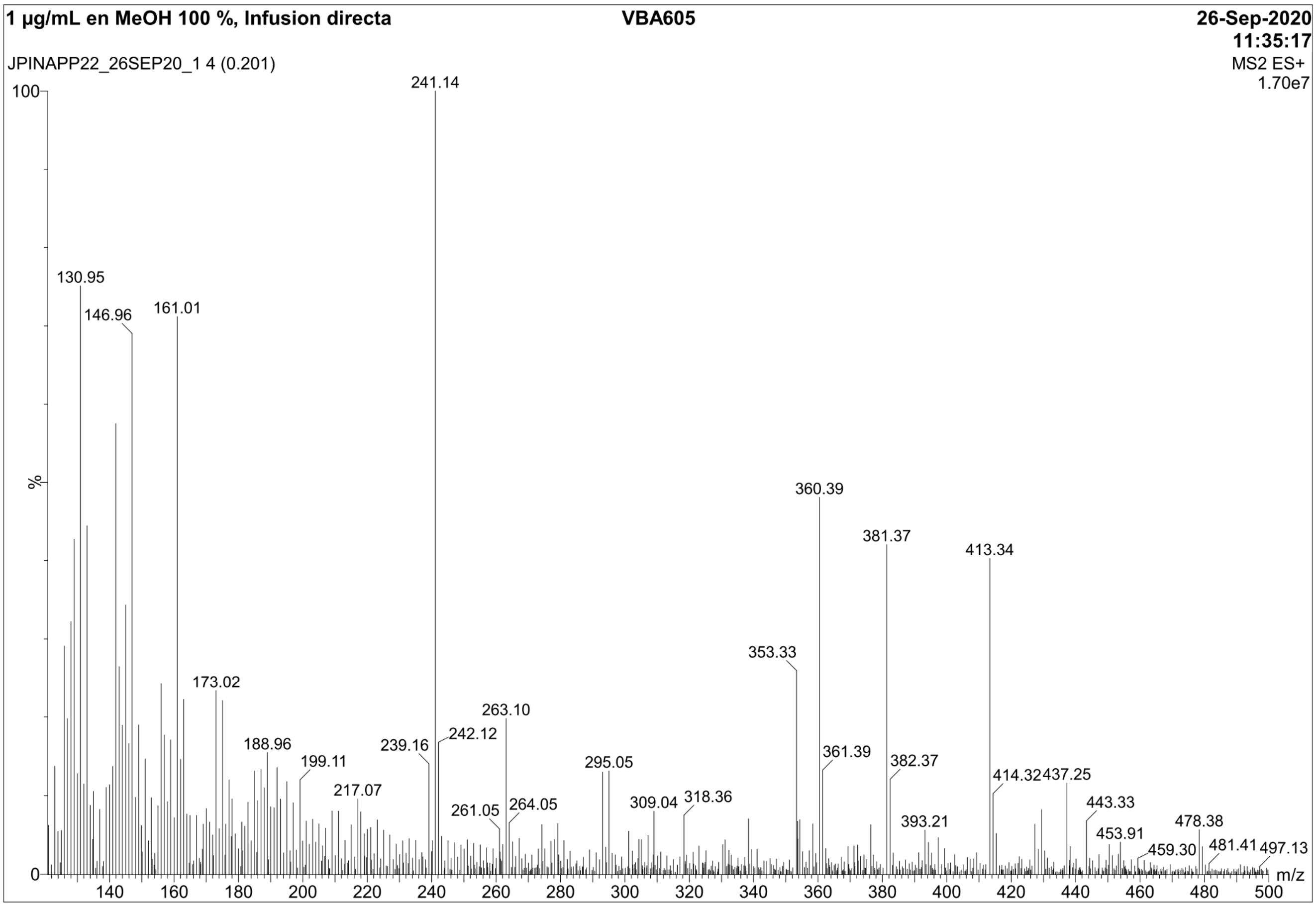

Figure S29. MS (EI) for 5-(1*H*-indazol-3-yl)-2-methoxyphenol (**9**).

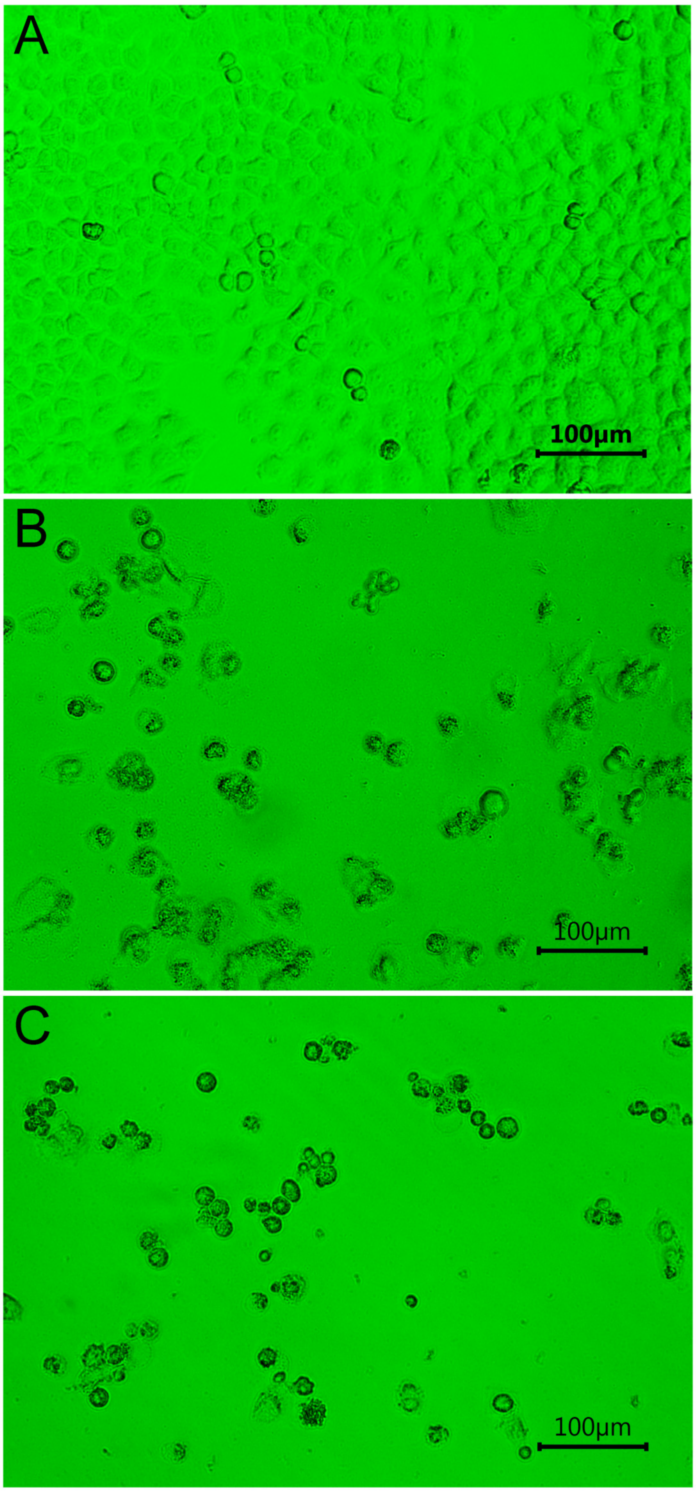

Figure S30. Morphological changes of HeLa cells after 48 h of treatment. **A**) vehicle (DMSO); **B**) Compound **5** (10 μM); **C**) Compound **6** (10 μM).

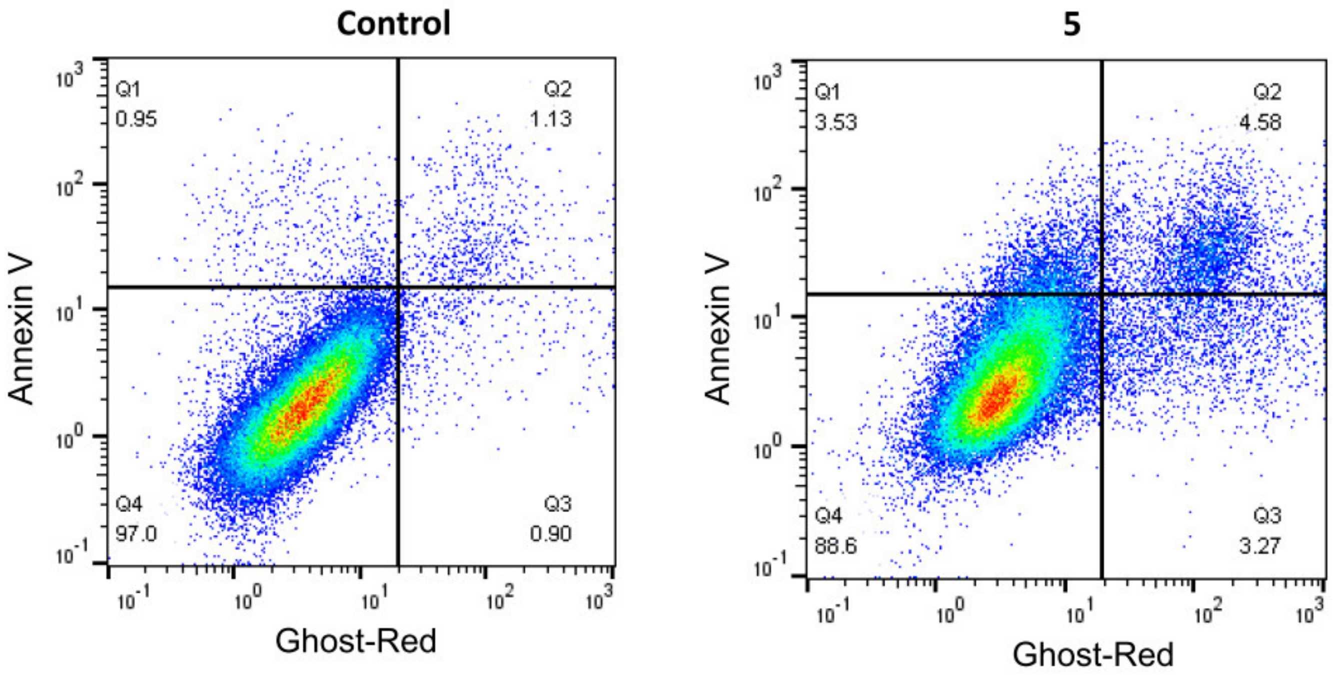

Figure S31. Quantitative apoptosis assay of HeLa using Annexin V-FITC/Ghost-Red. Biparametric representation obtained from the flow cytometry assay of control HeLa cells (Control) or treated with compound **5** (0.16 µM) at 48 h. Q4 (viable cells), the apoptosis ratios (early plus late apoptotic cells, Q3 + Q2 quadrants) and Q1 (necrotic cells).
